# ﻿On seven undescribed leaf insect species revealed within the recent “Tree of Leaves” (Phasmatodea, Phylliidae)

**DOI:** 10.3897/zookeys.1173.104413

**Published:** 2023-08-03

**Authors:** Royce T. Cumming, Stéphane Le Tirant, Jackson B. Linde, Megan E. Solan, Evelyn Marie Foley, Norman Enrico C. Eulin, Ramon Lavado, Michael F. Whiting, Sven Bradler, Sarah Bank

**Affiliations:** 1 Montreal Insectarium, 4581 rue Sherbrooke est, Montréal, H1X 2B2, Québec, Canada Montreal Insectarium Montréal Canada; 2 Richard Gilder Graduate School, American Museum of Natural History, New York, NY 10024, USA American Museum of Natural History New York United States of America; 3 Biology, Graduate Center, City University of New York, NY, USA City University of New York New York United States of America; 4 Department of Biology and M. L. Bean Museum, Brigham Young University, Provo, UT, USA Brigham Young University Provo United States of America; 5 Department of Environmental Science, Baylor University, Waco, TX, USA Baylor University Waco United States of America; 6 Westville, New Jersey, USA Unaffiliated Westville United States of America; 7 Saint Michael Academy-Catarman, Northern Samar, 6400 Philippines Saint Michael Academy-Catarman Northern Samar Philippines; 8 Department of Animal Evolution and Biodiversity, Johann- Friedrich-Blumenbach Institute of Zoology and Anthropology, University of Göttingen, Untere Karspüle 2, 37073, Göttingen, Germany University of Göttingen Göttingen Germany

**Keywords:** India, Indonesia, Kalimantan, Phasmida, Philippines, Seychelles, Vietnam, walking leaf

## Abstract

With the recent advance in molecular phylogenetics focused on the leaf insects (Phasmatodea, Phylliidae), gaps in knowledge are beginning to be filled. Yet, shortcomings are also being highlighted, for instance, the unveiling of numerous undescribed phylliid species. Here, some of these taxa are described, including *Phylliumiyadaon***sp. nov.** from Mindoro Island, Philippines; *Phylliumsamarense***sp. nov.** from Samar Island, Philippines; *Phylliumortizi***sp. nov.** from Mindanao Island, Philippines; *Pulchriphylliumheracles***sp. nov.** from Vietnam; *Pulchriphylliumdelislei***sp. nov.** from South Kalimantan, Indonesia; and *Pulchriphylliumbhaskarai***sp. nov.** from Java, Indonesia. Several additional specimens of these species together with a seventh species described herein, *Pulchriphylliumanangu***sp. nov.** from southwestern India, were incorporated into a newly constructed phylogenetic tree. Additionally, two taxa that were originally described as species, but in recent decades have been treated as subspecies, are elevated back to species status to reflect their unique morphology and geographic isolation, creating the following new combinations: *Pulchriphylliumscythe* (Gray, 1843) **stat. rev., comb. nov.** from Bangladesh and northeastern India, and *Pulchriphylliumcrurifolium* (Audinet-Serville, 1838) **stat. rev., comb. nov.** from the Seychelles islands. Lectotype specimens are also designated for *Pulchriphylliumscythe* (Gray, 1843) **stat. rev., comb. nov.** and *Pulchriphylliumcrurifolium* (Audinet-Serville, 1838) **stat. rev., comb. nov.** from original type material.

## ﻿Introduction

Stick and leaf insects (Phasmatodea; colloquially “phasmids”) are a clade that has evolved remarkable morphological adaptations for botanical camouflage and mimicry. Presently Phasmatodea includes more than 3,000 known species distributed across most regions of the world, with dozens of species new to science described annually (Bradler and Buckley 2018; Brock et al. 2022). While most phasmids resemble sticks, bark, and twigs, rarer instances of mimicry such as mosses, lichen, and leaves do exist, allowing phasmids to blend seamlessly into their arboreal habitat (Brock and Büscher 2022). Additionally helpful for their guise, these insects are typically nocturnal, with little to no movement during the day when visually oriented predators are most active, and even at night phasmid movements are often slow and deliberate, mimicking the swaying of leaves or branches (Bedford 1978; Bian et al. 2016).

**Figure 1.**
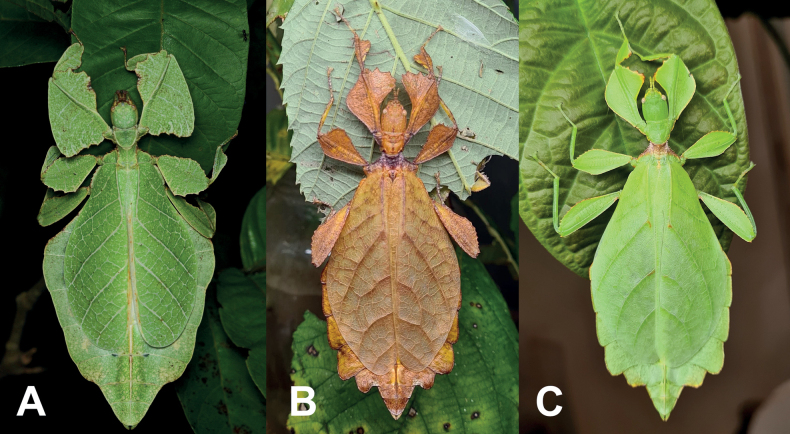
Live adult female leaf insects showing intergeneric and intraspecific color variability **A***Pulchriphylliumanangu* sp. nov. photographed near Agumbe, India on 17 May 2022 by iNaturalist user vishwanathgowda. Used with permission of Vishwanath Gowda (India) https://www.inaturalist.org/observations/118478029**B, C***Phylliumortizi* sp. nov. reared and photographed by Maxime Ortiz (France) in captivity **B** brown form female **C** green form female.

One clade which mimics general angiosperm leaf morphology particularly well are the Phylliidae, or true leaf insects. These masters of leaf masquerade are widely distributed across the tropical regions of Asia, Australasia, and the Pacific (Größer 2008; Bank et al. 2021; Brock et al. 2022). A dorso-ventrally flattened body form coupled with leaf-like venation patterns on the fore wings (tegmina) achieves the simulation of angiosperm leaves in female phylliids (Fig. 1; Ragge 1955). The diversity in coloration and patterns representing different stages of leaf decay lends to the remarkable mimicry of leaf insects (Fig. 1; Cumming et al. 2020d). Although predominantly green, the wide variety in coloration appears to be a response to specific environmental conditions (i.e., reflects phenotypic plasticity; Verrier 1929). Leaf insects exhibit strong sexual dimorphism with females being larger than males and having tegmina that extend over most of the abdomen but lack fully functional hind wings (Bank et al. 2021). In contrast, males possess shorter tegmina and have fully functional hind wings that allow for active flight (Boisseau et al. 2022). Another notable morphological feature that distinguishes the sexes is the antennae length, with males possessing long antennae that facilitate mate search and females having abbreviated antennae that enable defensive stridulation (Henry 1922).

Despite the stunning appearance of leaf insects, they are exceedingly rare within collections and in many cases solely known from the holotype (Cumming and Le Tirant 2022). In addition to their rarity in collections, many leaf insect species are morphologically very similar, or exhibit marked intraspecific variation (Hennemann et al. 2009; Cumming et al. 2020c), making them difficult to differentiate without a holistic approach to identification. This difficulty has led taxonomists of the past to focus on single or only a few prominent morphological features in an attempt to bring clarity to their higher classification (Griffini 1898; Rehn and Rehn 1933; Hennemann et al. 2009). These classifications, when based upon limited morphological features, led to poorly known species often being incorrectly associated with better-known clades (Cumming et al. 2021a, b; Bank et al. 2021; Cumming and Le Tirant 2022). Also, the inherent sexual dimorphism and variable morphological traits of phylliids have contributed to the misidentifications of opposite sexes as distinct and separate species, even to the extreme case of some species being placed in different genera (Cumming et al. 2020d). All of these difficulties have resulted in specimens from some clades being under-studied, leaving numerous phylliid species undescribed.

The relationship of the Phylliidae to the greater Phasmatodea has been debated for decades, with various authors placing them in dramatically different locations in the phasmid family tree (examples of past relationships summarized in Bank et al. 2021). Now, in recent decades as sample sizes have increased in both species and gene coverage, the phylogenetic results have begun to consistently recover the Phylliidae as a subordinate lineage within Phasmatodea (Simon et al. 2019; Tihelka et al. 2020; Bank and Bradler 2022). Unfortunately, sister-clade associations to the Phylliidae are still somewhat elusive as different inference methods and/or constraints often recover different tree topologies (Simon et al. 2019; Tihelka et al. 2020; Bank and Bradler 2022).

Despite the uncertainty of the higher-level phylogenetic relationship of the phylliids, the application of molecular data has been found to be a useful tool to clarify phylogenetic relationships within the Phylliidae (Cumming et al. 2020b, 2021b; Bank et al. 2021). Based on molecular data for the so far most comprehensive taxon sampling of leaf insects, Bank et al. (2021) was able to provide new insights on phylogenetic relationships within and among the major phylliid lineages as well as on their historical biogeography. Moreover, the phylogeny constructed therein emphasized not only the utility and need to assess phylliid relationships by including sequencing data but also by simultaneously expanding the morphological examination to include a variety of significant diagnostic characters. The results presented by Bank et al. (2021) provided the basis for a systematic revision of the major phylliid lineages and additionally revealed several undescribed species, seven of which are the focus of this work.

## ﻿Materials and methods

### ﻿Specimens

Specimens and observation records for this study come from permitted fieldwork conducted by the authors, records from reviewed museum collections, records from several private collections, and observations from citizen science-based platforms such as iNaturalist and biodiversity enthusiast groups on Facebook. For each species discussed herein, we explicitly state within Suppl. material 1 the number of specimens reviewed, their collection data/online source record, and/or the collection they are contained within to ensure the reproducibility of our work (note: specimen data listed within quotations is verbatim and is therefore in a variety of non-standard formats but is presented “as is” to ensure traceability of specimens utilized). Besides reviewing specimens in person, we also utilized the high-quality images of captive-reared specimens available publicly (http://www.phasmatodea.com) and many type material images (http://phasmida.speciesfile.org) to understand intraspecific variability.

Despite the usefulness of internal genitalia for differentiation of many insects, including phasmids (Ghirotto 2021), repeated investigations into phylliid genitalia as a useful tool for differentiation has found limited success. Attempts have been made within phylliids to identify internal genitalia features useful for differentiation, but what has repeatedly been found is that features were either too similar between species (therefore offering no differentiation ability) or that potential features were far too variable for reliable use (Größer 2011; Cumming et al. 2021b). Due to these results, phylliid internal genitalia have thus far only shown to be useful for differentiation of genera, not species (Bank et al. 2021). Therefore, within the following morphological descriptions, the genitalia are described as fully as is reasonable, but not exhaustively given the lack of usefulness found by past investigations. Within this work we discuss multiple species which are morphologically impossible to differentiate when based upon both the habitus as well as the genitalia, leaving these species only differentiable by molecular analyses.

Measurements of specimens were made to the nearest 0.1 mm using digital calipers and are given for individual specimens where applicable (such as for holotype specimens) or for series recording a minimum to maximum range (such as for paratype specimens). Images taken using: a Canon EOS 5DS R camera (Canon, Tokyo, Japan) with a 100-mm lens. Zerene Stacker (v. 1.04) was used to generate images from stacked photos (Zerene Systems LLC, Richland, WA, USA). Holotype and paratype specimens herein designated are deposited within several different institution collections, which are explicitly listed within the type material information of the new species descriptions and/or within Suppl. material 1, where the following collection acronyms are used:

**BYU**Brigham Young University, Monte L. Bean Life Science Museum, Provo, Utah, USA;

**Coll EF** Private collection of Evelyn Marie Foley, New Jersey, USA;

**Coll FH** Private collection of Frank H. Hennemann, Germany;

**Coll HMY** Private collection of H. M. Yeshwanth, Bangalore, India;

**Coll MO** Private collection of Maxime Ortiz, France;

**Coll MS** Private collection of Megan Solan, Texas, USA;

**Coll NE** Private collection of Norman Enrico C. Eulin, Samar, Philippines;

**Coll PEB** Private collection of Phil E. Bragg, Derbyshire, United Kingdom;

**Coll RC** Private collection of Royce T. Cumming, California, USA;

**Coll SLT** Private collection of Stéphane Le Tirant, Québec, Canada;

**IEBR** Entomological Collection of the Institute of Ecology and Biological Resources Hanoi, Vietnam;

**IMQC** Insectarium de Montréal, Montréal, Québec, Canada;

**MNHN**Muséum national d’Histoire naturelle, Paris, France;

**MZPW**Museum of the Zoological Institute of the Polish Academy of Sciences, Warsaw, Poland;

**MZSF**Université de Strasbourg, Musée Zoologique, Strasbourg, France;

**NHMUK**Natural History Museum United Kingdom, London, United Kingdom;

**NMS**National Museums Scotland, Edinburgh, United Kingdom;

**OUMNH** University Museum of Natural History, Oxford, United Kingdom;

**RBINS**Royal Belgian Institute of Natural Sciences, Brussels, Belgium;

**SASRD** School of Agricultural Sciences and Rural Development, Nagaland, India;

**ZSI**National Zoological Collections, Zoological Survey of India, Kolkata, India.

### ﻿Photography

Photographs of specimens deposited within the IMQC collection were taken using a Nikon D850 DSLR camera (Nikon Corporation, Tokyo, Japan) with Nikon Micro-Nikkor 200mm f/4 lens on Manfrotto 454 micrometric positioning sliding plate (Manfrotto, Casolla, Italy). Two Nikon SB-25 flash units provided lighting with a Cameron Digital diffusion photo box (Henry’s, Vancouver, Canada). Adobe Photoshop Elements 13 (Adobe Inc., San Jose, USA) was used as post-processing software. For details that could not be seen with the naked eye, such as the stridulatory file of the third antennomere in females, scanning electron microscopy (SEM) was used. Samples were sputter-coated with a 10 nm gold-palladium layer. SEM images were then taken with a SEM Hitachi TM3000 (Hitachi High-technologies Corp., Tokyo, Japan) at 15 kV acceleration voltage as outlined in Büscher et al. (2023).

### ﻿Molecular laboratory work and phylogenetic analysis

Bank et al. (2021) presented several undescribed *Phyllium* Illiger, 1798 and *Pulchriphyllium* Griffini, 1898 species and some species with tentative identification. By using the same dataset and including 15 additional specimens representing those and related taxa, we conducted a phylogenetic analysis to validate potentially new species. We generated 31 new sequences for up to six loci (16S, 18S, 28S, COI, COII, and H3) following the protocols given by Bank et al. (2021), except for one specimen (WS415 from India) for which data were obtained following the instructions given by Robertson et al. (2018) with Sanger dideoxy sequencing performed on an ABI 3730xl DNA Analyzer (Applied Biosystems Inc., Foster City, CA, USA). All sequences were deposited on GenBank and are made available in Suppl. material 2. For the phylogenetic analysis, we used the multiple sequence alignment produced by Bank et al. (2021) and added the newly obtained sequences for each gene separately by calling mafft v. 7.450 (Katoh and Standley 2013) with the options “--auto --keeplength --addfragments” (Katoh and Frith 2012). The extended single-gene alignments were then concatenated using FASconCAT v. 1.1 (Kück and Meusemann 2010), with the resulting supermatrix containing 184 taxa and corresponding in length and partitioning to the original (Suppl. material 3). Thus, we used the same partitioning scheme and substitution models inferred by Bank et al. (2021) to reconstruct the phylogenetic tree with IQ-TREE v. 2.2.0 as described therein (Nguyen et al. 2015; Chernomor et al. 2016; Minh et al. 2020). Also following Bank et al. (2021), we assessed support using 10,000 ultrafast bootstrap trees (Hoang et al. 2018) and the SH-aLRT single branch test with 10,000 replicates (Guindon et al. 2010).

## ﻿Results

### ﻿Phylogenetic analysis

**Figure 2.**
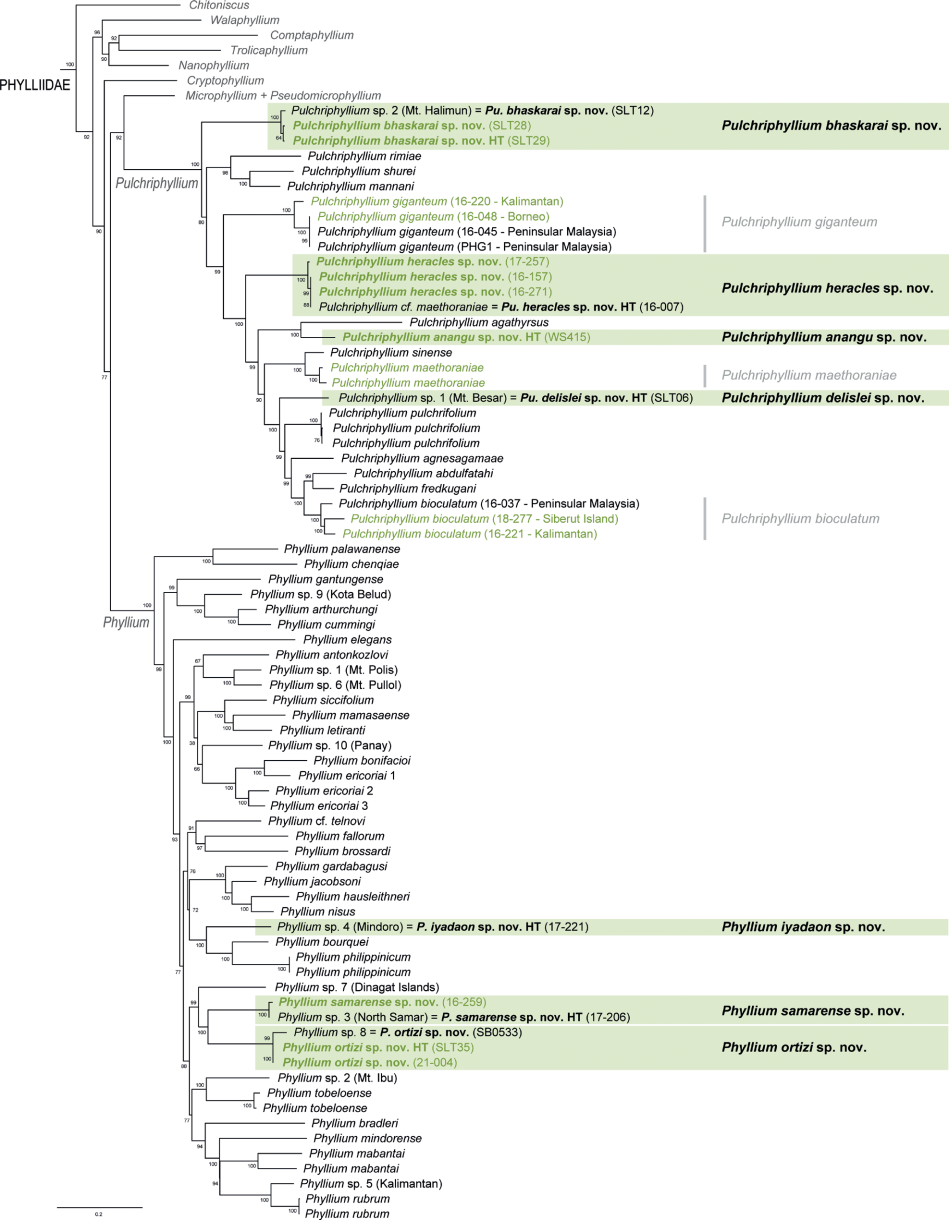
Phylogenetic tree of the Phylliidae based on six nuclear and mitochondrial loci with a focus on *Phyllium* and *Pulchriphyllium* (please refer to Suppl. material 4 for the complete tree). Ultrafast bootstrap support values are depicted at each node. Specimens in green font are in addition to the already existing tree inferred by Bank et al. (2021). Highlighted in green boxes are the seven newly described species.

The phylogenetic relationships of 169 taxa with 15 newly added specimens resulted in an almost identical phylogeny to that inferred by Bank et al. (2021) (Fig. 2; Suppl. material 4). The Phylliidae and all its genera were recovered with high to maximum support. Within *Pulchriphyllium*, *Pulchriphyllium* sp. 1 (Mt. Besar) was recovered with the same topology as in Bank et al. (2021) and determined to be an undescribed species. *Pulchriphyllium* sp. 2 and two additional specimens from Mt. Halimum (Java, Indonesia) were found to form a distinct clade which was recovered as a sister taxon to the remaining *Pulchriphyllium*. The specimen from Agumbe Ghats (WS415; Karnataka, India) was also recovered as a distinct species sister to *Pulchriphylliumagathyrus* (Gray, 1843) from Sri Lanka. Within *Phyllium*, the newly added specimens from Samar and Mindanao Island (Philippines) were recovered to cluster with *Phyllium* sp. 3 and *Phyllium* sp. 8, respectively, forming two separate and distinct species. *Phyllium* sp. 4 from Mindoro was found to be sister taxon to a clade formed by *Phylliumphilippinicum* Hennemann, Conle, Gottardo & Bresseel, 2009 and *Phylliumbourquei* Cumming & Le Tirant, 2017.

### ﻿Taxonomy

**Phylliidae, Phylliinae, Phylliini**

#### ﻿ Phyllium

Taxon classificationAnimaliaPhasmatodeaPhylliidae

Illiger, 1798

215121B7-ED94-5382-A2CF-94D6CD9C2096

##### Type species.

*Phylliumsiccifolium* (Linnaeus, 1758), type locality: “Indies”.

*Phyllium* historically encompassed most leaf insect species, with numerous internal subdivisions and species groups, some recognized as unique for more than a century (e.g., *Pulchriphyllium* Griffini, 1898) and other divisions more recent (e.g., those found in Hennemann et al. 2009; Cumming et al. (2019a, 2020a). Within the first phylliid-wide molecular-based phylogeny, which covered nearly all recognized genera/subgenera/species groups (Bank et al. 2021), *Phyllium* was adjusted only to include those species which form a monophyletic clade that shares significant morphological features with the type species *Phylliumsiccifolium* (Linnaeus, 1758).

#### ﻿ Phyllium samarense

Taxon classificationAnimaliaPhasmatodeaPhylliidae

sp. nov.

743A4AD2-23BD-58CD-9259-CBAA6E3912CE

https://zoobank.org/52538350-3294-40A1-B553-8DF4E47E31F5

**Figure 3.**
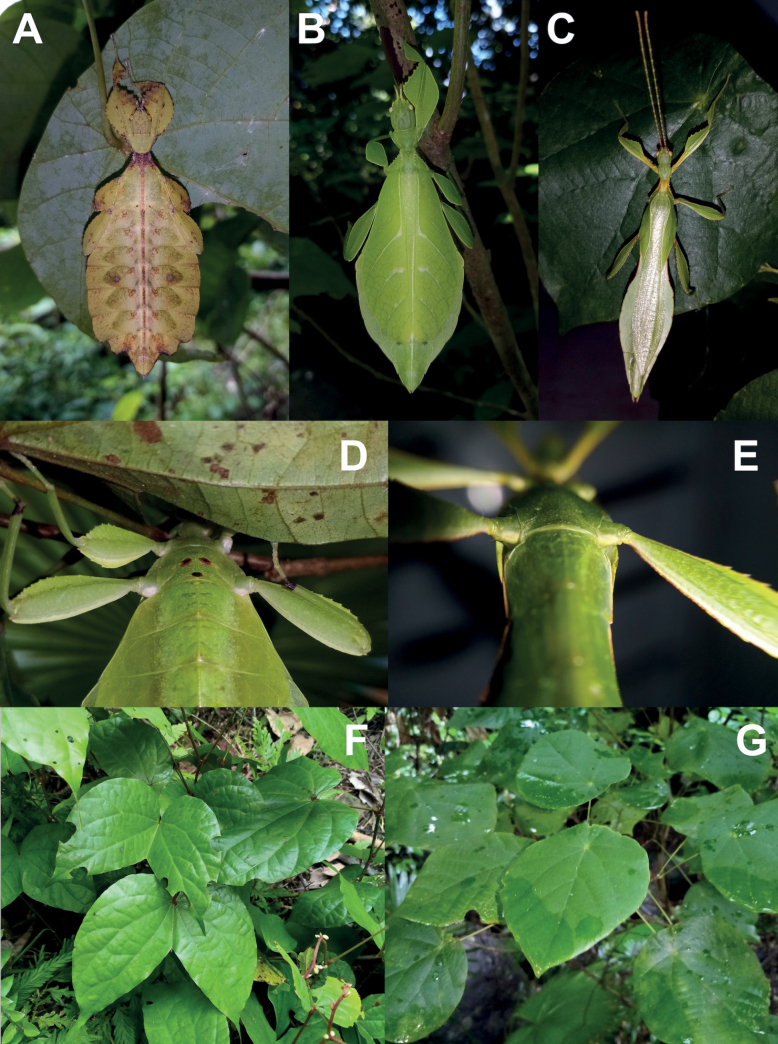
Live *Phylliumsamarense* sp. nov. observed in the wild **A** female nymph, dorsal habitus, found feeding on *Mallotusfloribundus* (Euphorbiaceae) **B** adult female, dorsal habitus, found feeding on *Mallotusfloribundus* (Euphorbiaceae) **C** adult male, dorsal habitus **D** detail of the coxae, ventral, female **E** detail of the coxae, ventral, male **F***Phanera* sp. (Fabaceae) **G***Mallotusfloribundus* (Euphorbiaceae).

**Figure 4.**
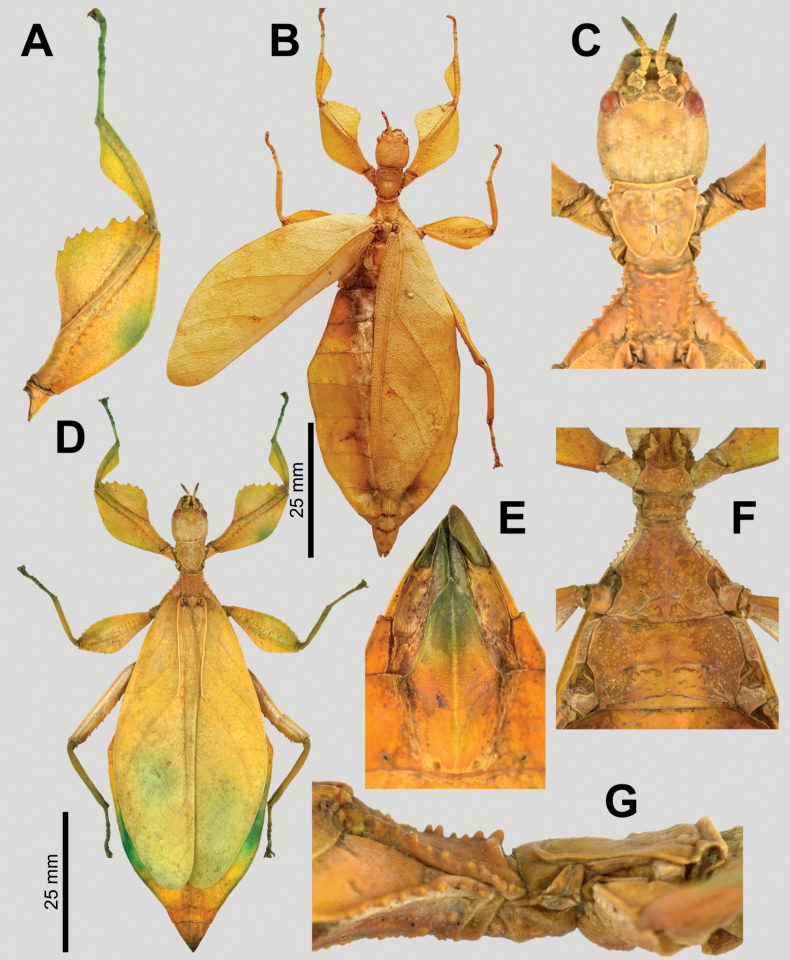
*Phylliumsamarense* sp. nov. female type material **A, C–G** paratype (IMQC) **B** holotype (IMQC), photographs by René Limoges (IMQC) **A** details of antennae, head capsule, and thorax, dorsal **B** habitus, dorsal **C** profemoral and protibial lobes, dorsal **D** habitus, ventral **E** genitalia, ventral **F** thorax details, lateral. Scale bars: 25 mm (**B, D**).

**Figure 5.**
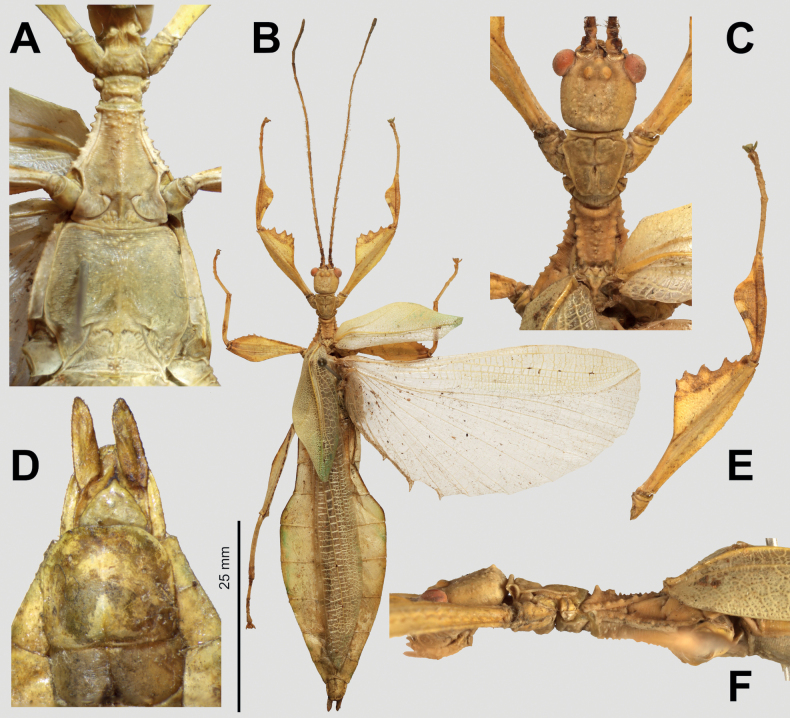
*Phylliumsamarense* sp. nov. male paratype, Coll RC #16-259 **A** details of thorax, ventral **B** habitus, dorsal **C** details of head and thorax, dorsal **D** genitalia, ventral **E** details of front leg, dorsal **F** thorax details, lateral. Scale bar: 25 mm (**B)**.

**Figure 6.**
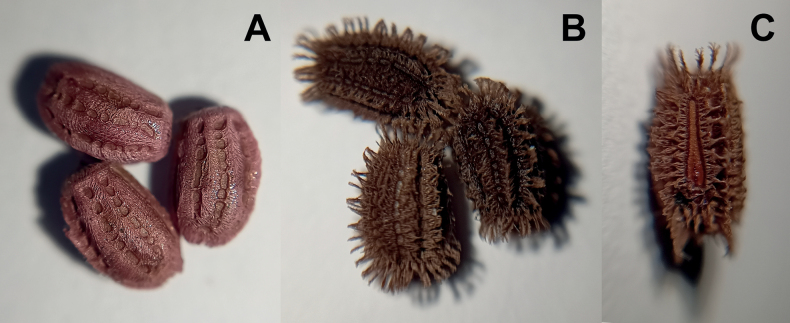
*Phylliumsamarense* sp. nov. eggs, collected from the female in Fig. 3B**A** freshly laid eggs which have not yet encountered water, lateral view **B** eggs after being exposed to high humidity so the frills expand, lateral view **C** detail in dorsal view of the micropylar plate on a single egg.

**Figure 7.**
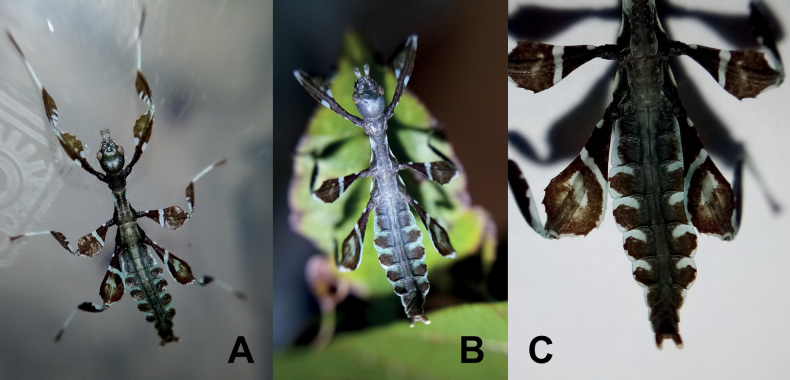
*Phylliumsamarense* sp. nov. freshly hatched nymph reared in captivity **A** dorsal habitus **B** dorsal habitus **C** details of the abdomen and back legs.

**Figure 8.**
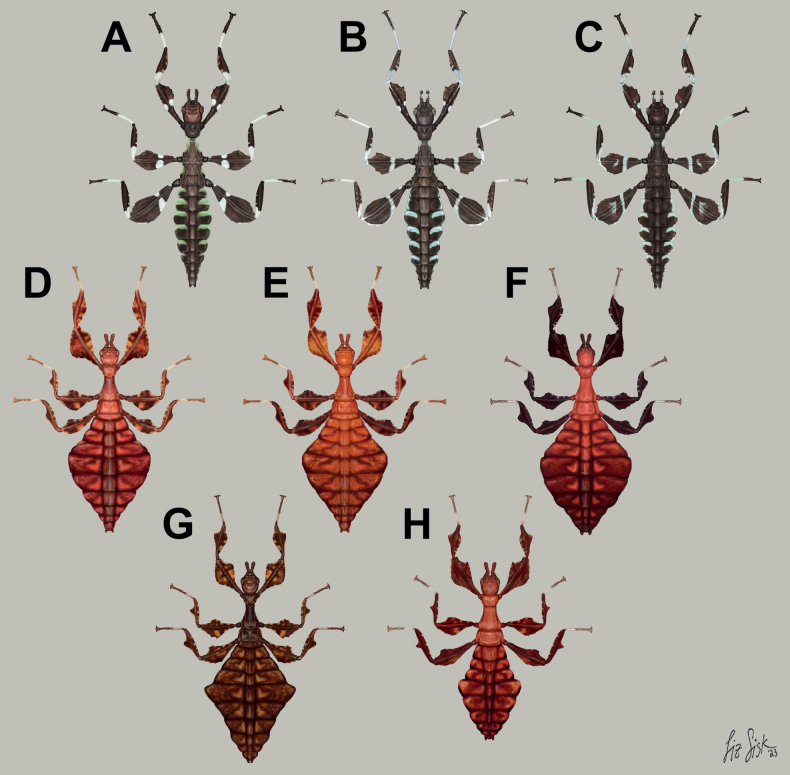
Illustrations of the freshly hatched nymph coloration for herein referenced species **A***Phylliumortizi* sp. nov. (Mindanao) **B***Phylliummabantai* (Mindanao) **C***Phylliumsamarense* sp. nov. (Samar) **D***Pulchriphylliumbioculatum* (West Malaysia) **E***Pulchriphylliumanangu* sp. nov. (Southwest India) **F***Pulchriphylliumagathyrsus* (Sri Lanka) **G***Pulchriphylliumgiganteum* (West Malaysia) **H***Pulchriphylliumbhaskarai* sp. nov. (Java). Illustrations by scientific illustrator Liz Sisk (Washington D.C., USA).

Figs 3A–E , 4 , 5 , 6 , 7 , 8C

##### Material examined.

***Holotype*** ♀: “Philippines: Eastern Visayas, Northern Samar, Lope De Vega: April, 2017; Coll RC 17-206; DNA ID #W9”. Deposited in the Montreal Insectarium, Quebec, Canada (IMQC). ***Paratypes***: (9 ♀♀, 6 ♂♂, 1 ♂ nymph, 2 ♀♀ nymphs, 6 eggs). See Suppl. material 1 for details about paratype specimens, their collection data, and depositories.

##### Differentiation.

**Female***Phylliumsamarense* sp. nov. (Figs 3A, B, 4) are morphologically most similar to *Phylliummabantai* Bresseel, Hennemann, Conle & Gottardo, 2009 and *Phylliummindorense* Hennemann, Conle, Gottardo & Bresseel, 2009 based upon the overall size, tegmina venation, and femoral lobe shape/serration. From *Phylliummindorense*, *Phylliumsamarense* sp. nov. can be differentiated by having a distinct sagittal spine on the prescutum anterior rim (*Phylliummindorense* rim lacks a spine), also the protibial interior lobe in *Phylliumsamarense* sp. nov. is slightly weighted more towards the distal end (vs in *Phylliummindorense* where the interior lobes widest point is the middle). From *Phylliummabantai*, *Phylliumsamarense* sp. nov. can be differentiated by the ventral coloration of the coxae, as it is white in *Phylliumsamarense* sp. nov. (Fig. 3D) but burnt orange/reddish-brown in *Phylliummabantai*.

**Male***Phylliumsamarense* sp. nov. (Figs 3C, 5) are most similar to *Phylliummabantai* based upon the thorax spination and shape, and the abdomen which is spade shaped. *Phylliumsamarense* sp. nov. males can be differentiated by the location of the media posterior (MP) vein split in the tegmina as *Phylliumsamarense* sp. nov. has the split on the distal ⅓ of the tegmina, but in *Phylliummabantai* the split is closer to the middle of the tegmina.

**Eggs** of *Phylliumsamarense* sp. nov. (Fig. 6) are most similar to *Phylliumphilippinicum* Hennemann, Conle, Gottardo & Bresseel, 2009 and *Phylliummabantai* based upon the pinnae morphology, pinnae arrangement, and overall egg shape. On *Phylliumsamarense* sp. nov. eggs the lateral margin pinnae arrangement encircling the capsule is rather similar to *Phylliummabantai*, but *Phylliumsamarense* sp. nov. can be differentiated by having anterior pinnae that are similar in size to the lateral pinnae (vs *Phylliummabantai* anterior pinnae which are notably larger than the lateral pinnae). Additionally, these two species can be differentiated by the lateral surface longitudinal bald patches which are smaller and more regular in *Phylliumsamarense* sp. nov. vs *Phylliummabantai* where these bald impressions are larger and more variable with areas of connection where one area connects with another. The lateral surface pinnae and bald markings on *Phylliumsamarense* sp. nov. are very similar to those found on *Phylliumphilippinicum* but these species can be differentiated by the dorsal and ventral margin pinnae as *Phylliumphilippinicum* has the dorso-anterior and most of the ventral margin lacking prominent pinnae, vs *Phylliumsamarense* sp. nov. which has prominent pinnae fully encircling the capsule.

Freshly hatched nymph *Phylliumsamarense* sp. nov. (Figs 7, 8C) are most similar to *Phylliummabantai* (Fig. 8B) and *Phylliumortizi* sp. nov. (Fig. 8A) based upon the bicolored abdominal segments). *Phylliumsamarense* sp. nov. can easily be differentiated from both by the presence of pale irregularly shaped markings on the femoral lobes in addition to the typical stripe/broken stripe (Fig. 7C) that is present on the other species.

##### Description.

**Female. *Coloration*.** Coloration description is based upon living individuals in which some variation has been observed. Overall coloration has been observed to range from fully pale green (Fig. 3B) to muddled yellow/tan (Fig. 3A). Certain variable areas (such as the lobes of the legs, the thorax, abdominal margins, and the venation in the tegmina) can either match the base coloration or be tan/brown/reddish at times. Ventral coxae coloration is white (Fig. 3D).

***Morphology*. *Head*.** Head capsule slightly longer than wide; vertex is irregularly lumpy due to moderately sized nodes throughout the surface and a base texture which is slightly wrinkled (Fig. 4C). The posteromedial tubercle is the most prominent node on the head capsule, rising above the other nodes. Frontal convexities broad and blunt, with a slight granular surface and marked throughout by singularly protruding short setae. Compound eyes not strongly protruding from the head capsule and take up ca ¼ of the head capsule lateral margins (Fig. 4C). ***Ocelli absent*.** Antennal fields slightly wider than the width of the first antennomere. ***Antennae*.** Antennae consist of nine segments, with the terminal segment approximately the same length as the preceding 1½ segments’ lengths combined (Fig. 4C). Antennomeres I–VII sparsely marked with short transparent setae, the terminal two segments are covered in stout, densely packed, darker setae. The stridulatory file on the third antennomere has 34 or 35 teeth packed side by side without a distinct gap between teeth. ***Thorax*.** Pronotum with slightly concave anterior margin and somewhat convex lateral margins, which converge to a straight posterior margin that is ½ the width of the anterior margin (Fig. 4C). The pronotum surface is smooth, with only a prominent pit in the center, and slight furrows anterior and lateral to the pit (Fig. 4C). The pronotum has well-formed anterior and lateral rims and a moderately formed posterior rim, all of which are relatively smooth (Fig. 4C). Prosternum marked throughout by moderately sized and spaced nodes, and the prosternum surface is relatively flat, lacking any prominent projections (Fig. 4F, G). Meso- and metasternum surfaces are irregularly marked with small to medium sized nodes with the mesosternum having larger nodes along the anterior ½ along the sagittal plane and the metasternum having more prominent nodes along the lateral margins (Fig. 4F). The prescutum is slightly longer than wide, lateral rims with 12 or 13 well-formed, uniformly sized tubercles (Fig. 4C). Prescutum anterior rim prominent and moderately protruding, rim surface smooth, rim marked by a distinct but not large sagittal spine (Fig. 4C, G). Prescutum surface smooth and raised slightly along the sagittal ridge which is marked by six or seven nodes that are at most ca ½ the size of the anterior rim spine, some slightly smaller, all relatively evenly spaced (Fig. 4C). Mesopleurae begin to diverge slightly posterior to the anterior prescutum rim, and they diverge evenly with relatively straight margins. Mesopleurae lateral margin with five or six larger tubercles, four or five moderately formed tubercles, and several nodes interspersed throughout the length (Fig. 4C). Mesopleura surface smooth but marked with two notable divots, one on the anterior margin and one slightly posterior to the middle (Fig. 4C). ***Wings*.** Tegmina long, reaching ⅓ to ½ of the way onto abdominal segment VIII. Tegmina venation; the subcosta (Sc) is the first vein in the forewing, and it runs parallel with the lateral margin for the first ½, then it bends slightly and runs to the margin where it terminates on the margin edge ca ¼ of the way through the wing length. The radius (R) spans the central portion of the forewing with two subparallel veins that branch from the radius; the first radius (R1) branches ca ⅕ of the way through the wing length and terminates ca ⅓ of the way through the wing length; and the radial sector (Rs) branches ca ⅓ of the way through the wing length, arcs gently through the central portion of the wing and terminates near the distal ⅓ of the wing. There is a thinner continuation of the radius following the prominent Rs branching which continues on as a short R–M crossvein that weakly connects the two veins. The media (M) is bifurcate with the media anterior (MA) splitting slightly posterior to ½ of the wing length and the media posterior (MP) splits near the posterior ¼ of the tegmina. Both the media anterior and media posterior terminate near the apex of the tegmina. The cubitus (Cu) is also bifurcate, branching near the posterior ⅕ of the wing into the cubitus anterior (CuA) and cubitus posterior (CuP) which both terminate at or very near the wing apex. The first anal vein (1A) is simple and fuses with the cubitus early on, ca ¼ of the way through the tegmina length. Alae rudimentary, no more than just a nub (Fig. 4B). ***Abdomen*.** Abdominal segments II through the anterior ⅔ of IV uniformly diverging with straight margins. The posterior ⅓ of segment IV through segment VI have margins which are straight and converging slightly towards the posterior. Segment VII through the abdomen apex are converging at a stronger angle than the previous segments but also have relatively smooth, straight margins, giving the abdomen an overall spade-shaped appearance (Fig. 4D). ***Genitalia*.** Subgenital plate starts at the anterior margin of tergum VIII, is moderately broad, and extends halfway under tergum X with margins that are converging gradually and straight for the majority of the length and then near the apex they bend slightly and converge more sharply to a fine point (Fig. 4E). Gonapophyses VIII are long and not notably broad, with their tips reaching the apex of the abdomen; gonapophyses IX are shorter and narrower, hidden below gonapophyses VIII (Fig. 4E). Cerci mostly flat, interior margin near the apex slightly cupped, surface relatively smooth with only a few setae and mild granulation along the margin (Fig. 4E). ***Legs*.** Profemoral exterior lobe broad, fully arcing from end to end, slightly narrower than the width of the interior lobe (Fig. 4A). Margin of the profemoral exterior lobe granular and marked with four or five small, dulled teeth (Fig. 4A). Profemoral interior lobe ca 2½× wider than the greatest width of the profemoral shaft, obtusely angled, and marked with six or seven serrate teeth that vary slightly in their size and spacing. The size of the profemoral interior lobe teeth appear to be somewhat variable as some specimens have slightly more prominent teeth than others (Fig. 4A). Mesofemoral exterior lobe reaches from end to end with straight margins and a gentle bend in the center, with the distal ½ slightly wider than the proximal ½, and the distal ½ only marked with two to five small serrate teeth. Mesofemoral interior lobe is approximately the same width as the mesofemoral shaft, and the exterior lobe is slightly wider than the mesofemoral shaft. Mesofemoral interior lobe arcs end to end but is slightly wider on the distal ½ of the arc, and only the distal ½ is ornamented with six or seven serrate teeth. Metafemoral interior lobe arcs end to end, with the distal ½ slightly wider than the proximal ½ and marked with seven to ten serrate teeth on the distal ½ of the lobe. Metafemoral exterior lobe is thin and smooth, hugging the metafemoral shaft and lacks dentition. Protibiae lacking an exterior lobe (Fig. 4A). Protibiae interior lobe spans the entire length of the protibiae and is ca 1½× the width of the protibiae shaft itself. The lobe is roundly triangular with the widest portion slightly distal to the midline. Mesotibiae and metatibiae simple, lacking exterior and interior lobes.

***Measurements of holotype female* [mm].** Length of body (including cerci and head, excluding antennae) 78.7, length/width of head 7.3/6.3, antennae 4.5, pronotum 5.4, mesonotum 7.1, length of tegmina 48.6, length of alae 4.3, greatest width of abdomen 29.3, profemora 15.2, mesofemora 12.9, metafemora 16.0, protibiae 9.2, mesotibiae 10.0, metatibiae 13.5.

***Measurements of paratype females* [mm].** Length of body (including cerci and head, excluding antennae) 76.4–80.1, length/width of head 7.2–7.4/6.3– 6.4, antennae 4.5–4.6, pronotum 5.2–5.4, mesonotum 7.0–7.4, length of tegmina 47.5–49.7, length of alae 4.3–4.4, greatest width of abdomen 28.2–30.2, profemora 15.1–15.6, mesofemora 12.7–13.2, metafemora 16.0–16.4, protibiae 9.2–9.5, mesotibiae 10.0–10.4, metatibiae 13.5–13.8.

**Male. *Coloration*.** Coloration based upon living individuals (Fig. 3C). Overall coloration pale green throughout with highlights of tan/reddish coloration. Tan areas are primarily the thorax, compound eyes, antennae, and bases of the femora. The margins of the abdomen are more of a rich red color. Abdominal segment V has a pair of slightly transparent eye spots that are not prominent. Ventral coxae coloration is white (Fig. 3E).

***Morphology*. *Head*.** Head capsule slightly longer than wide, with a vertex that is slightly lumpy with sparse irregularly spaced but relatively uniformly sized nodes throughout the posterior of the capsule. The posteromedial tubercle is singularly pointed and distinctly raised above the head capsule (Fig. 5C, F). Frontal convexities stout and bluntly pointed with sparse setae. Compound eyes large and bulbous, occupying ca ⅖ of the head capsule lateral margins (Fig. 5C). There are three well-developed ocelli distinctly raised above the capsule and located between the compound eyes (Fig. 5C). ***Antennae*.** Antennae (including the scapus and pedicellus) consist of 25 segments, all segments except the scapus and pedicellus and terminal five segments are covered in dense setae where most are as long as or slightly longer than the antenna segment is wide. The terminal five segments are covered in dense short setae and the scapus and pedicellus are nearly completely bare with only a few sparse setae. ***Thorax*.** Pronotum with anterior and lateral margins that are relatively straight and converging to a straight posterior margin that is ca ½ the width of the anterior margin (Fig. 5C). Anterior and lateral margins of the pronotum have distinct rims and the posterior margin has a weakly formed rim (Fig. 5C). Face of the pronotum is marked by a distinct pit in the center, a sagittal furrow on the anterior ½, and slight perpendicular furrows originating from the central pit. The pronotum surface is only slightly lumpy but lacking distinct granulation (Fig. 5C). Prosternum surface is lumpy with small nodes (Fig. 5A). Mesosternum surface anterior third marked heavily with granulation, the remainder of the mesosternum surface is wrinkled but lacks notable nodes (Fig. 5A). Metasternum surface mostly wrinkled throughout, and the anterior margin central area is additionally marked with granulation. Prescutum longer than wide, with lateral margins that are somewhat converging to the posterior margin which is only slightly narrower than the anterior margin (Fig. 5C). Lateral margins of the prescutum with six or seven moderately formed tubercles with most of a uniform size (Fig. 5C). Prescutum surface slightly raised along the sagittal plane which is marked with five or six more prominent nodes and the remainder of the surface has slight granulation (Fig. 5C, F). Prescutum anterior rim moderately formed with a distinct sagittal spine, and the remainder of the rim surface is relatively smooth (Fig. 5C, F). Mesopleurae begin on the anterior prescutum margin and diverge at a gradually increasing angle from the anterior to the posterior but are never notably wide throughout their length (Fig. 5C). Mesopleuron lateral margin with seven or eight moderately formed tubercles and a few small nodes interspersed throughout the length except for the posterior ¼ of the margin which is relatively smooth (Fig. 5C). Mesopleuron face moderately wrinkled and marked by a distinct pit near the center. ***Wings*.** Tegmina moderate length, extending ½ of the way onto abdominal segment III. Tegmina wing venation: the subcosta (Sc) is the first vein, is simple, and terminates ca ½ of the way through the overall wing length. The radius (R) spans the entire length of the tegmina with the first radius (R1) branching just proximal to the midline and terminating near the distal ⅓ of the tegmina, followed by the radial sector running straight to the wing apex. The media (M) also spans the entire length of the tegmen running side by side along the radius/radial sector with the media posterior (MP) branching off near the distal ⅓ of the tegmen and running angled towards the apex/cubitus, and the media anterior (MA) runs straight to the tegmen apex. The cubitus (Cu) cuts across the tegmen to the margin ca ⅓ of the way through the length and runs along the edge of the wing where the media posterior vein fuses with it and as the cubitus reaches the apex it fades. The first anal (1A) vein terminates upon reaching the cubitus slightly < 1/2 of the way through the tegmen length. Alae well-developed in an oval fan configuration, long, reaching to the middle or posterior of abdominal segments IX. Ala wing venation: the costa (C) is present along the entire foremargin giving stability to the wing. The subcosta (Sc) is long, spanning ca ⅗ of the wing length and is mostly fused with the radius near the base of the wing but terminates when it meets the costa. The radius (R) spans the entire wing and branches slightly proximal to the midline into the first radius (R1) and radial sector (Rs) which run gently diverging for ca ½ of their length, then run parallel until they near the apex of the ala where they begin to converge slightly but they terminate at the margin near each other but not touching. The media (M) branches early, ca ⅙ of the way through the ala length into the media anterior (MA) and the media posterior (MP) which run parallel with each other throughout the wing until the distal ⅓ the media posterior fuses with the media anterior and they run fused to join with the radial sector and this fused set of veins runs to the apex where it terminates. The cubitus (Cu) runs unbranched and terminates at the wing apex. Of the anterior anal veins, the first anterior anal (1AA) fuses with the cubitus near the ala base and then the first anterior anal branches from the cubitus ⅔ of the way through the ala length where it uniformly diverges from the cubitus until it terminates at the wing margin. The anterior anal veins two–seven (2AA–7AA) have a common origin and run unbranched in a folding fan pattern to the wing margin. The posterior anal veins (1PA–6PA) share a common origin separate from the anterior anal veins and run unbranched to the wing margin with slightly thinner spacing than the anterior anal veins. ***Abdomen*.** Lateral margins of abdominal segment II parallel, III diverging slightly, IV diverging at a more prominent angle to the widest point, V parallel sided or slightly subparallel (converging slightly), VI through X converging gradually with smooth margins, giving the abdomen a spade-shaped appearance. ***Genitalia*.** Poculum broad and ends in an apex that slightly passes the anterior margin of the abdominal segment X with a margin that is straight (Fig. 5D). Cerci long and slender, with slightly > ½ of their length extending from under abdominal segment X, nearly flat, covered in a granulose surface and numerous short setae with those along the margins slightly longer (Fig. 5D). Vomer broad and stout with straight sides evenly converging for the proximal ¾ and then near the apical hook the margins converge more sharply to the apical hook which is thick and has a singular point (Fig. 5D). ***Legs*.** The profemoral exterior lobe is narrow, approximately the same width as the profemoral shaft at its widest. The profemoral exterior lobe margin is slightly granular and on the distal ½ there are three or four weakly formed, dulled teeth (Fig. 5E). The profemoral interior lobe is obtusely triangular and at its greatest width it is slightly > 2× the greatest width of the profemoral shaft. The profemoral interior lobe is ornamented on the distal ½ with five serrate teeth arranged in a three-two pattern with looping gaps between them, where the central three teeth are notably larger than the first and last teeth (Fig. 5E). Mesofemoral exterior lobe arcs end to end but is slightly wider on the distal ⅓ which has a distinct bend and on this distal portion it can be bare or be marked with as many as three teeth, while the proximal ½ of the lobe is rather thin and lacks teeth. Mesofemoral interior lobe, exterior lobe, and mesofemoral shaft are all approximately the same width. The mesofemoral interior lobe, is broader on the distal end which is rounded (not as strongly bent as on the exterior lobe) and the distal end is ornamented with six or seven small serrate teeth while the proximal portion of the lobe is thin and lacks teeth. Metafemoral exterior lobe lacks dentition and has a straight margin along the metafemoral shaft. Metafemoral interior lobe smoothly arcs end to end with nine or ten sharply serrate teeth on the distal ½, which is wider than the smooth proximal portion of the lobe. Protibiae lacking exterior lobe, interior lobe reaching end to end in a rounded triangle with the widest portion on the distal ½ and at its widest the lobe is ca 2–2⅓× as wide as the protibial shaft width (Fig. 5C). Meso- and metatibiae simple, lacking lobes completely.

***Measurements of paratype males* [mm].** Length of body (including cerci and head, excluding antennae) 57.6–60.2, length/width of head 3.9–4.5/3.3– 3.5, antennae 33.2–35.1, pronotum 2.9–3.1, mesonotum 4.6–4.8, length of tegmina 19.0–19.6, length of alae 42.8–43.3, greatest width of abdomen 14.2–14.5, profemora 11.6–12.0, mesofemora 10.2–10.5, metafemora 12.7–13.1, protibiae 7.6–8.0, mesotibiae 7.3–7.5, metatibiae 9.7–10.1.

**Eggs.** (Fig. 6). The overall color is uniformly brown (slightly pinker in color when freshly laid before moisture contact; Fig. 5A), pinnae of a similar color to the capsule. The lateral surfaces are nearly flat (only slightly convex), with the posterior of the egg is slightly wider than the anterior. The pinnae on the capsule surfaces are variable with some short and “carpet-like” (not having significant lateral frills, typically shorter and dense) up to larger “feather-like” pinnae (which are longer, sometimes with a split apex, and short frills along their lengths). The feather-like pinnae are mostly along the margins, anterior, and posterior, while the carpet-like pinnae mark the surfaces. The lateral surfaces of the capsule are marked throughout with the shorter, dense pinnae arranged in longitudinal rows separated by three longitudinal rows of bald impressions formed by partially connected ovals. The dorsal surface has a long micropylar plate (spanning approximately the central ¾ of the capsule length) with a width that is almost uniform throughout (only slightly wider around the micropylar cup). The micropylar cup is located on the posterior ⅖ of the capsule (Fig. 5C). On either side of the micropylar plate is shorter, dense pinnae. The operculum is slightly ovular, the surface is flat and covered in dense short pinnae, and the outer margin is rimmed with larger feather-like pinnae (Fig. 5C). The ventral surface of the egg capsule has a surface similar to the lateral surfaces with short pinnae and longitudinal bald impressions.

***Measurements including the extended pinnae* [mm].** Length (including operculum): 4.5–5.0; maximum width of capsule when viewed from lateral aspect 2.5–3.0; length of micropylar plate 2.7–3.0.

##### Newly hatched nymphs.

(Fig. 7). The general color throughout the body is a rich dark brown. The basitarsi are cream colored and the remaining tarsal segments are of a similar dark brown to the rest of the body. All tibiae lack exterior lobes but do have interior lobes. The protibiae have a smoothly arcing interior lobe which is ca 2× wider than the protibial shaft width and the meso- and metatibiae have interior lobes ca 1½× as wide as the shaft they are on. All tibiae have a similar color pattern with the proximal ⅓–½ a striped/patchy white/cream with the remainder rich brown. The profemora have a base color of rich brown marked with three white patches, two larger and prominent white spots (one on each side of the profemoral shaft) on the proximal ⅖ along with one smaller and less defined white spot on the interior lobe closer to the center of the length. The meso- and metafemora have similar coloration, both with the base coloration rich brown, a small white spot near the base of the interior lobe, an unbroken white stripe running from the interior lobe to the exterior lobe located on the proximal ⅖, and the distal margin of the exterior lobe is also marked with a white edge. The meso- and metafemora differ by their size (metafemora are larger) and by an additional, irregular white patch near the middle of the femora (smaller and fainter in the mesofemora and larger and brighter white on the metafemora). All femoral interior lobes and the meso- and metafemoral exterior lobes have distinct serration on the distal ends. The head and pronotum are mostly dark brown/black and marked by lighter colored nodes. The mesothorax and metathorax are mostly brown but are marked with pale lime green margins and white speckling. The abdomen base color is brown, with the center of the abdomen fully brown. The thin lobes on each side of the abdomen are bicolored. Segments II and III have white margins, segments IV through VII have roughly the anterior ⅖ white and the posterior ⅕ white, and segments VIII through X are mostly brown and are only marked minimally on the lateral margin with white. The abdomen general shape is long and narrow, with a maximum width < ½ of the abdomen length. The widest point of the abdomen is abdominal segment IV. The first four antennae segments are brown, the central three are white, and the terminal two are brown. Overall length of freshly hatched nymphs ca 13–14 mm.

##### Etymology.

Toponym. Named after the type locality Samar Island, Philippines. As the first officially endemic species of *Phyllium* from the island, we wished to highlight this significant find by referencing the island.

##### Distribution.

At present only known from North Samar province, on Samar Island, Philippines in the Eastern Visayas region.

##### Remarks.

This population was previously thought to be a range expansion for *Phylliummabantai*, a species which is quite variable in abdominal morphology and has a similar overall habitus (Cumming 2017). Interestingly, despite the similar size, thorax shape and spination, and leg shapes with *Phylliummabantai*, this new species from Samar was recovered as sister species to *Phylliumortizi* sp. nov., which is morphologically notably different with exaggerated abdominal lobes (Fig. 10). Most observed specimens of *Phylliumsamarense* sp. nov. have a rather tapered abdomen (Fig. 3B) although a few records have been found which have slight lobes on abdominal segments VII and VIII (Fig. 3A), suggesting that like several other *Phyllium* species, the *Phylliumsamarense* sp. nov. abdominal shape can be variable (Cumming et al. 2020c). As in other *Phyllium* species, despite females having variable abdominal shapes, the males so far observed have all been uniform in their morphology (Fig. 3C).

To date, *Phylliumsamarense* sp. nov. has been found feeding on three host plants in the wild; *Phanera* sp. (Fabaceae; Fig. 3F), *Mallotusfloribundus* (Euphorbiaceae; Fig. 3G), and *Terminalia catappa* (Combretaceae).

The *Phylliumsamarense* sp. nov. egg morphology fits within the results of Büscher et al. (2023) which recovered most *Phyllium* species as having pinnae “type 5” (defined within as: feather-like and hierarchically splitting pinnae with a broad base and several side branches). Within the recovered egg morphology ancestral state reconstruction pinnae “type 5” was recovered as the likely ancestral state for the *Phyllium*, and *Phylliumsamarense* sp. nov. adds an additional species supporting this recovered state (Büscher et al. 2023).

#### ﻿ Phyllium iyadaon

Taxon classificationAnimaliaPhasmatodeaPhylliidae

sp. nov.

B9A061D9-998A-580E-80D8-7131E8F9A68D

https://zoobank.org/1EFD93F9-9343-4FD4-BE96-2A135A3D6951

Fig. 9

##### Material examined.

***Holotype*** ♂: “Philippines: Mindoro, Puerto Galera: April, 2017; Coll RC 17-221”. Deposited in the Montreal Insectarium, Quebec, Canada (IMQC). ***Paratype***: (1♂) • “Philippines: Mindoro, Puerto Galera: April, 2017; Coll RC 17-222” (Coll RC).

##### Differentiation.

**Figure 9.**
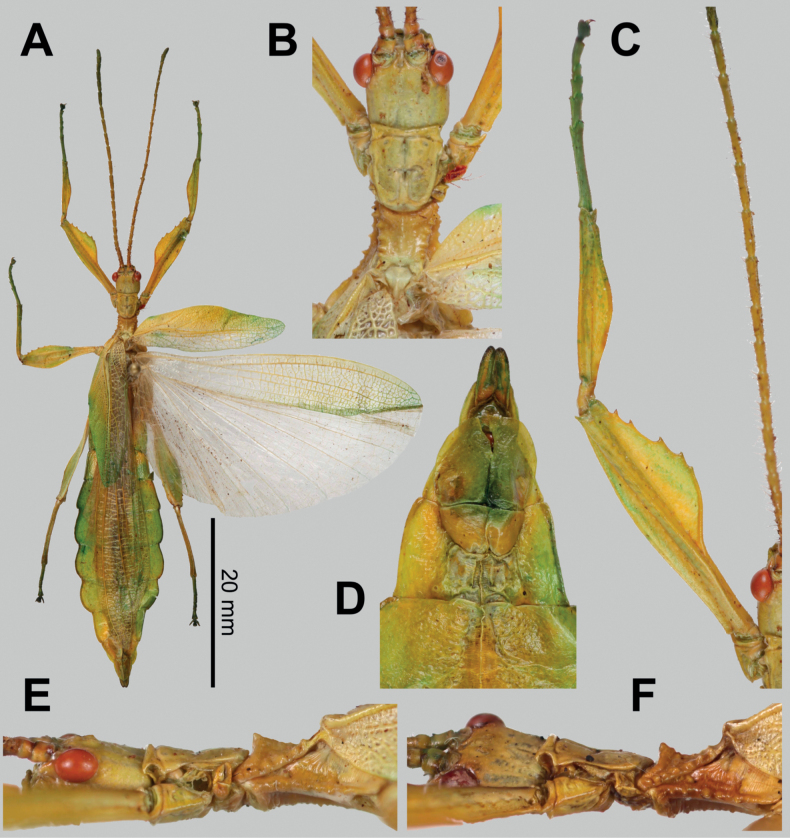
*Phylliumiyadaon* sp. nov. type specimens **A–E** holotype (IMQC) Coll RC 17-221 **F** paratype (Coll RC) Coll RC 17-222 **A** habitus, dorsal **B** details of the head through thorax, dorsal **C** details of the front leg, dorsal **D** genitalia, ventral **E** details of the head through thorax, lateral **F** details of the head through thorax, lateral. Scale bar: 20 mm (**A)**.

Female, freshly hatched nymph, and egg unknown. Males morphologically are similar to *Phylliumphilippinicum* and *Phylliumbourquei* in all features except for the undulating abdomen, the profemoral interior lobe with a notable space between the third and fourth tooth, and the sagittal crest of the mesonotum differs by having a prominent anterior spine with the remainder of the sagittal crest having weakly formed tubercles (Fig. 9E, F).

**Male. *Coloration*.** Coloration description based on the dried type specimens. Coloration is pale green with variable areas of straw yellow throughout likely due to the drying process, particularly if alcohol was used to help preserve the specimens (Fig. 9). Compound eyes are burnt red (Fig. 9B) and the anterior seven or eight segments of the antennae are darker green than the other segments which are a similar off-yellow color like the discolored patches on the body (Fig. 9C). All abdominal segments are of a similar color, lacking eye spots.

***Morphology*. *Head*.** Head capsule ca ¼ longer than wide, with a vertex that is slightly lumpy, but smooth, lacking distinct granulation; posteromedial tubercle is not prominent, only slightly raised above the head capsule (Fig. 9B). Frontal convexities stout, only slightly projecting with blunt rounded apexes. Compound eyes are large and bulbous, occupying slightly < ½ of the head capsule lateral margins (Fig. 9B). Between and slightly posterior to the compound eyes are three moderately formed ocelli (Fig. 9B). Antennal fields are slightly wider than the scapus width. ***Antennae*.** Antennae (including the scapus and pedicellus) consists of 25 segments. The scapus and pedicellus are smooth, lacking setae, all other segments are covered evenly in thin, dark setae which in most cases are as long as or slightly longer than the antennal segment is wide. The terminal four antennal segments have short, dense setae, which are more numerous than the setae on the other segments. ***Thorax*.** Pronotum rectangular in shape, with a length that is ⅕ longer than the width (Fig. 9B). Pronotum anterior margin slightly concave; lateral margins are nearly straight for the anterior ¾ of their length but then converge slightly for their remainder to a straight posterior margin that is ca ¾ the width of the anterior rim (Fig. 9B). Anterior and lateral margins of the pronotum with distinct rims and the posterior margin with a moderately formed rim (Fig. 9B). Face of the pronotum is marked by a relatively smooth surface (only slightly wrinkled in places and lacking distinct nodes). Face of the pronotum has a distinct sagittal furrow, a notable central pit, a moderately formed perpendicular furrow just anterior to the central pit, and two distinct pits along the anterior margin behind the anterior rim (Fig. 9B). Prosternum with distinct and numerous granulations throughout, evenly spaced. Mesosternum anterior surface and sagittal plane marked with similar granulation to the prosternum surface, followed by more prominent wrinkles and less granulation gradually to the posterior. Metasternum distinctly wrinkled on the anterior and lateral margins, posterior with granulation slightly more prominent than the granulation on the prosternum. Prescutum as long as the anterior rim is wide, with lateral margins only slightly converging to the posterior which is ca ⅙ narrower than the anterior rim width (Fig. 9B). Lateral rims with seven to nine tubercles of varying sizes, typically five or six are larger than the others, the smallest one or two can be notably reduced but still distinct (Fig. 9B). The surface of the prescutum is slightly lumpy but smooth with the lateral surfaces rising slightly up to meet the prescutum sagittal crest (Fig. 9B). The prescutum crest along the sagittal plane is connected to the anterior margin spine, and slopes posteriorly from the anterior to the posterior, lacking distinct tubercles (Fig. 9E, F). Prescutum anterior rim distinctly raised into a prominent spine rising notably above the pronotum surface (Fig. 9E, F). Mesopleura narrow, diverging only slightly on the anterior ½, and then distinctly widening on the posterior ½ but not projecting strongly away from the body, thus giving them an overall slender appearance (Fig. 9B). Mesopleuron lateral margin with seven to nine moderately sized tubercles situated on the anterior ⅔ of the length with the posterior ⅓ mostly bare or at most with only a slightly undulating surface or weakly formed tubercles (Fig. 9B). Face of the mesopleuron distinctly wrinkled and with two distinct pits, one on the anterior ⅓ and one on the posterior ⅓. ***Wings*.** Tegmina moderate length, extending ca ¼ onto abdominal segment IV. Tegmen wing venation: the subcosta (Sc) is the first vein and it runs gradually to ca ⅜ of the way through the overall tegmen length and terminates on the margin. The radius (R) spans the entire length of the tegmen, running as the radial sector (Rs) straight through the center of the tegmen to the apex after the first radius (R1) branches ca ⅓ through the length of the wing and runs approximately to the posterior ⅓ margin where it terminates. The media (M) spans the entire length of the tegmen, running parallel with the radius (R) and radial sector (Rs) and terminates at the wing apex as the media anterior (MA) after the branching of a weakly formed media posterior (MP) near the middle of the tegmen which terminates slightly posterior to the tegmen midline. The cubitus (Cu) runs through the tegmen surface angled away from the media (M) for ca ⅓ of the length to the tegmen margin and then runs along the margin to near but not meeting with the apex. The first anal (1A) vein runs subparallel to the cubitus until it meets it ca ¼ of the way through the tegmen length. Alae well-developed in an oval fan configuration, reaching ca ½ through abdominal segment IX. Ala wing venation: the costa (C) is present along the entire foremargin giving stability to the wing. The subcosta (Sc) is fused with the radius for the anterior ½ and terminates when it meets the costa slightly < ½ of the way through the wing length. The radius (R) branches ca ⅓ of the way through the ala length into the first radius (R1) and radial sector (Rs) which run gradually diverging through most of their length until near the wing apex when they run parallel briefly then converge near but not touching the wing apex. The media (M) branches early, ca 1/7 of the way through the wing into the media anterior (MA) and the media posterior (MP) which run parallel with each other until the distal ¼ of the wing where the media posterior fuses with the media anterior and they run fused towards the media posterior, but do not appear to fuse with it fully, instead simply fading. The cubitus (Cu) runs unbranched and terminates at the wing apex. Of the anterior anal veins, the first anterior anal (1AA) fuses with the cubitus slightly distal to the point where the media branches into the media anterior and media posterior and then the first anterior anal branches from the cubitus ¾ of the way through the wing length where it uniformly diverges from the cubitus until it terminates at the wing margin. The anterior anal veins 2–7 (2AA–7AA) have a common origin and run unbranched in a folding fan pattern of relatively uniform spacing to the wing margin. The posterior anal veins (1PA–6PA) share a common origin separate from the anterior anal veins and run unbranched to the wing margin with slightly thinner spacing than the anterior anal veins. ***Abdomen*.** Abdomen general shape is narrow with a maximum width only ca ⅓ wide as long. Abdominal segment II parallel sided, III slightly diverging and terminating in a small spur on the posterior margin, IV-VII individually diverging and then converging to create an undulating margin, giving the abdomen a lobed appearance. Abdominal segments VIII through X converge gradually to the narrow apex. ***Genitalia*.** Poculum ovular in overall shape, broad with lateral margins exceeding the width of abdominal segment IX, ending in a straight margined apex which passes beyond the anterior margin of abdominal segment X (Fig. 9D). Cerci long and slender, with ca ½of their lengths extending from under abdominal segment X (Fig. 9D). The cerci are relatively flat, not strongly cupped, and have a weakly granular surface and sparse setae throughout. Vomer broad and stout with slightly convex sides converging evenly to the apex, which is armed with a singular upwards turning hook. ***Legs*.** Profemoral exterior lobe arcing thinly through its length with a margin that is only finely granular (none prominent), with the greatest width of the lobe only slightly wider than the profemoral shaft itself, and distinctly thinner than the greatest width of the profemoral interior lobe (Fig. 9C). Profemoral interior lobe beginning slightly proximal to ½ of the overall shaft length in a rounded scalene triangle with the shorter proximal edge lacking teeth, but the distal edge marked by teeth arranged in a three-two pattern with a gap notably wider between these sets of small, serrate, anteriorly pointing teeth (Fig. 9C). The profemoral interior lobe is ca 2× as wide as the profemoral shaft at its widest. Mesofemoral exterior lobe arcs end to end with the widest portion near the distal ⅓ of the length which is approximately as wide as the mesofemoral shaft at its widest, for the proximal ⅓ of the length the lobe is thin and hugging the mesofemoral shaft. The mesofemoral exterior lobe lacks teeth, only ornamented on the distal end in a singular spine. The mesofemoral interior lobe is ca the same width as the exterior lobe and is similarly weighted from end to end with the widest portion near the distal ⅓. The distal ½ is marked with five serrate teeth which begin small and increase in size towards the distal end. Metafemoral exterior lobe lacks dentition and has a smooth margin arcing thinly along the metafemoral shaft. Metafemoral interior lobe is smooth and straight on the proximal ½ but then arcs gently and is marked by six serrate, fine teeth on the distal ½. Protibia lacking exterior lobe, interior lobe reaching end to end in a thin rounded isosceles triangle slightly < 2× as wide as the protibial shaft, with the widest point near the midline (Fig. 9C). Mesotibiae and metatibiae simple, lacking lobes completely.

***Measurements of holotype male*Coll RC 17-221 [mm].** Length of body (including cerci and head, excluding antennae) 50.3, length/width of head 2.8/2.9, antennae 27.3, pronotum 2.8, mesonotum 3.8, length/width of tegmina 18.4/5.6, length of alae 35.8, greatest width of abdomen 10.7, profemora 10.4, mesofemora 8.9, metafemora 11.2, protibiae 7.4, mesotibiae 7.1, metatibiae 8.5.

***Measurements of paratype male*Coll RC 17-222 [mm].** Length of body (including cerci and head, excluding antennae) 52.1, length/width of head 2.8/2.9, antennae damaged and missing most segments, pronotum 2.8, mesonotum 3.8, length/width of tegmina 18.6/5.7, length of alae 36.4, greatest width of abdomen 10.8, profemora 10.4, mesofemora 8.8, metafemora 11.3, protibiae 7.4, mesotibiae 7.1, metatibiae 8.6.

##### Etymology.

Noun, Alangan in origin, meaning “shy leaf”. We wish to honor the original inhabitants of the area that this species is native to by using a language local to northern Mindoro. We choose the threatened language, Alangan, which is spoken by as few as 2,150 speakers in north-central Mindoro (according to Ethnologue: Languages of the World; ethnologue.com). The name is formed from the Alangan words iyá (meaning shy) + daon (meaning leaf; Barbian 1977). This specific epithet was chosen to reference, first, how rarely seen this species is (as it is only known from two specimens), and second, leaf insects with their excellent camouflage can reasonably be considered to be “shy” creatures, blending into their surroundings and actively avoiding detection.

##### Distribution.

Currently only known from the type locality Puerto Galera, Mindoro Island, Philippines.

##### Remarks.

Mindoro, as a smaller island, was originally thought to only have one *Phyllium* species present, *Phylliummindorense* Hennemann, Conle, Gottardo & Bresseel, 2009, and based upon examination of multiple specimens from this island, *Phylliummindorense* appears to be the most commonly collected species. However, a morphologically distinct set of males which did not match with the known male *Phylliummindorense* morphology were reviewed. These specimens were included within the analyses of Bank et al. (2021) as “*Phyllium* sp. 4” to ascertain if they were a morphological variation of *Phylliummindorense*, or a distinct, yet to be described species. Interestingly, these specimens were recovered as sister species to *Phylliumphilippinicum* Hennemann, Conle, Gottardo & Bresseel, 2009 and *Phylliumbourquei* which are species from nearby Luzon.

One possible identification which was explored is that these could represent the unknown male sex of *Phylliumbilobatum* Gray, 1843, which unfortunately is only known from a single holotype specimen and the inexact locality of “Philippine Islands” (Gray 1843). Based upon the phylogenetic recovery and morphological similarity to *Phylliumphilippinicum* and *Phylliumbourquei*, we find it unlikely that these Mindoro males represent the opposite sex of *Phylliumbilobatum* (a species which appears to be more morphologically similar to *Phylliumortizi* sp. nov. based upon the smaller size, prominent femoral lobes and serration, and strongly lobed abdominal segments). The size of these two *Phylliumiyadaon* sp. nov. type specimens (ca 50–52 mm in length) fall within the size range for *Phylliumphilippinicum* males (49.0–56.5 mm; Hennemann et al. 2009) and given these species’ close phylogenetic relationship, it is probable that the female *Phylliumiyadaon* sp. nov. may be of a similar size to *Phylliumphilippinicum* females (which range in size from 77.5–88.0 mm; Hennemann et al. 2009). The holotype female *Phylliumbilobatum* on the contrary is only 65.0 mm long and therefore these *Phylliumiyadaon* sp. nov. males are probably not the opposite sex of *Phylliumbilobatum* as they are presumably too large. These two *Phylliumiyadaon* sp. nov. male type specimens are the only representatives of this species known to us at this time and we hope that a fresh female specimen can one day be located and confirmed as the opposite sex to allow comparison with the little known *Phylliumbilobatum* female.

#### ﻿ Phyllium ortizi

Taxon classificationAnimaliaPhasmatodeaPhylliidae

sp. nov.

360A0306-8F3F-56D0-B980-D54EC87C5828

https://zoobank.org/92E1EBF0-AD20-46A2-9B4B-4002E951661B

**Figure 10.**
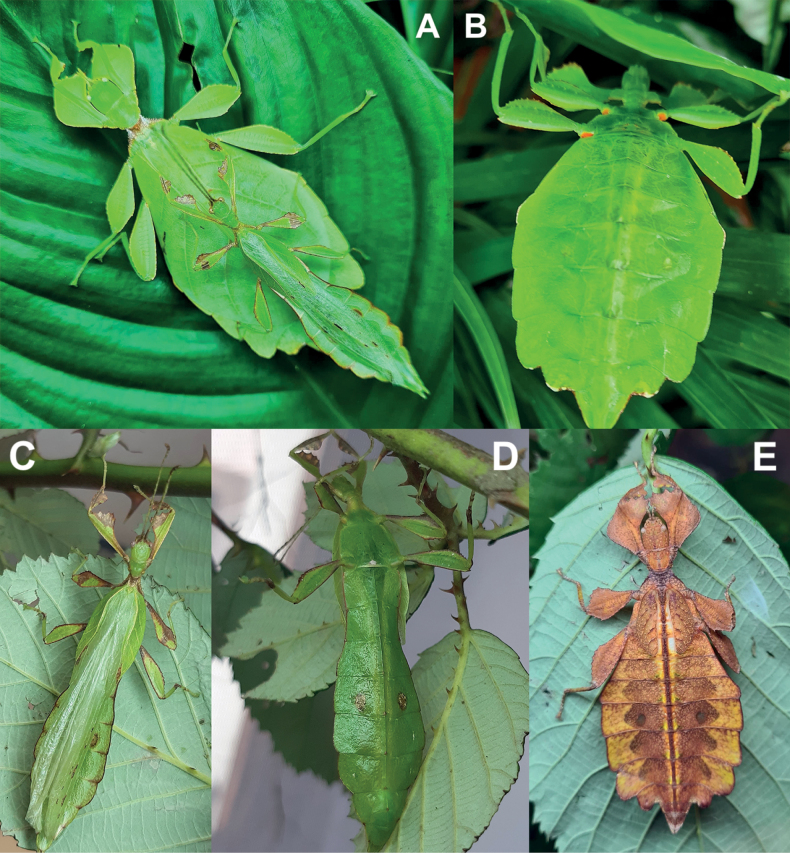
*Phylliumortizi* sp. nov. live individuals from captive culture highlighting aspects of their coloration. Photographs by Maxime Ortiz (France) **A** adult male and female pair, dorsal habitus **B** adult female, postero-ventral view, showing the bright orange coxae coloration **C** adult male, habitus, dorsal **D** adult male, habitus, ventral **E** brown form nymph, same individual as in Fig. 1B before the final molt.

**Figure 11.**
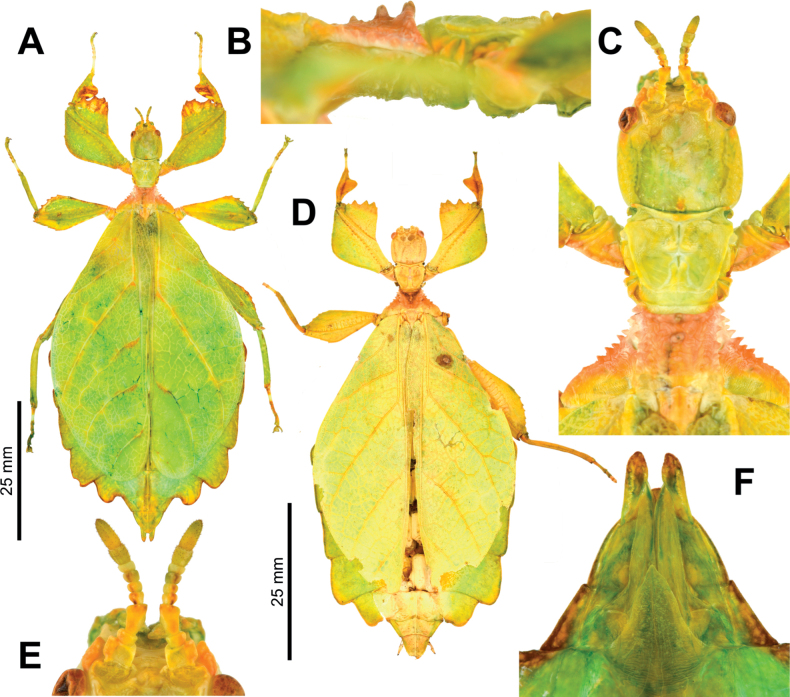
*Phylliumortizi* sp. nov. female holotype (IMQC) **D**, paratype (Coll RC) **A–C, E, F** photographs by René Limoges (IMQC). Scale bars associated with **A, D** respectively **A** dorsal habitus, paratype **B** lateral view of the thorax showing spination **C** details of the head and thorax, dorsal **D** dorsal habitus, holotype **E** details of the antennae, dorsal **F** genitalia details, ventral.

**Figure 12.**
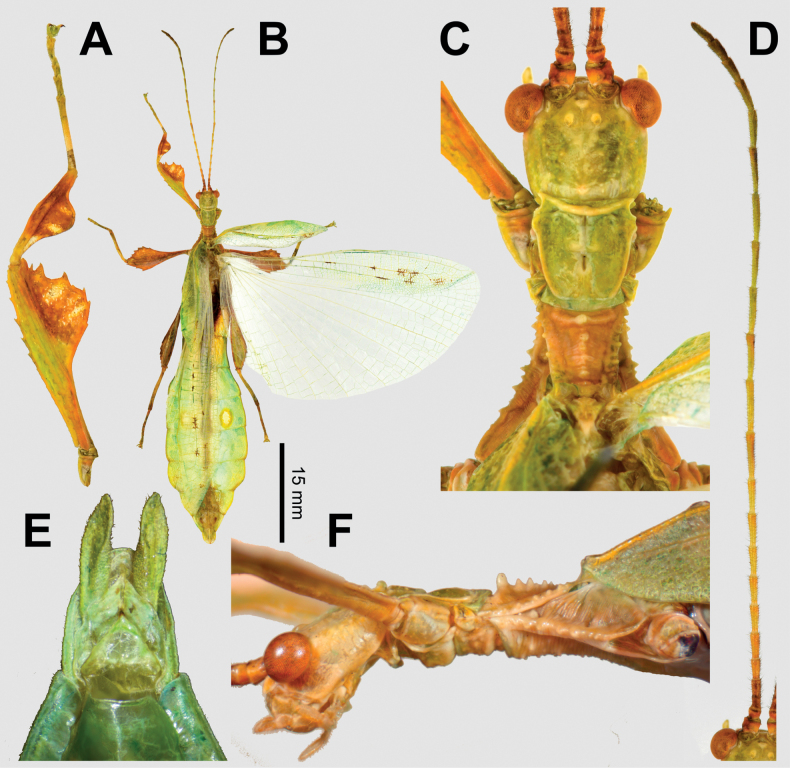
*Phylliumortizi* sp. nov. male paratypes **A–D** (Coll RC 21-001). Photographs by René Limoges (IMQC) **E, F** (Coll RC 21-002) **A** details of front leg **B** dorsal habitus **C** details of the head and thorax, dorsal **D** antenna, dorsal **E** genitalia details, ventral **F** lateral view of the thorax showing spination. Scale bar: 15 mm (**B)**.

**Figure 13.**
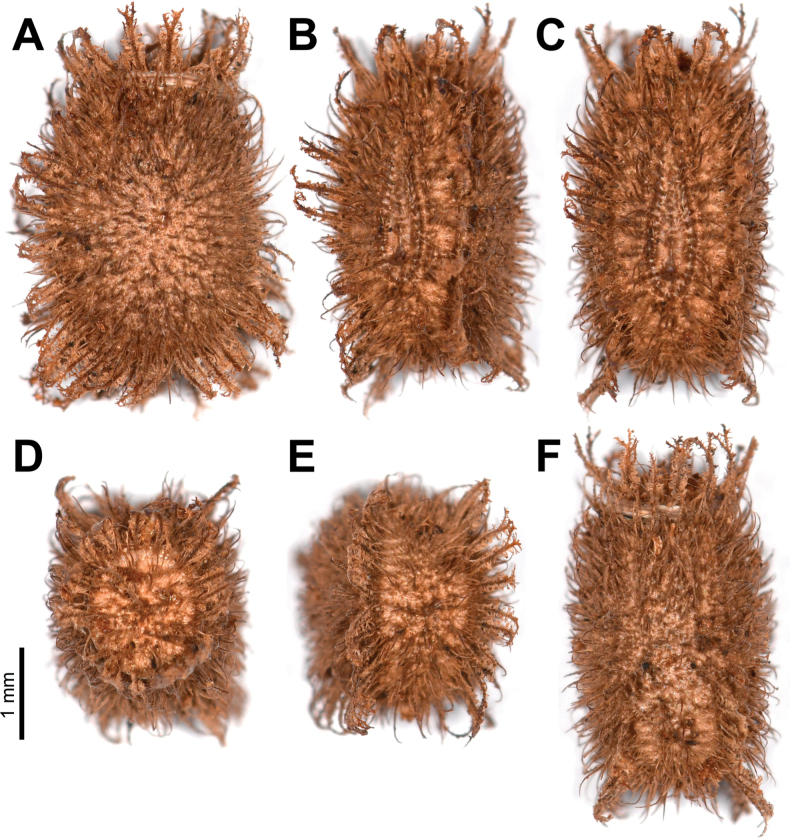
*Phylliumortizi* sp. nov. egg capsule, IMQC collection, photographs by René Limoges (IMQC), scale bar associated with all images **A** lateral view **B** dorso-lateral view **C** dorsal view **D** opercular (anterior) view **E** posterior view **F** ventral view.

**Figure 14.**
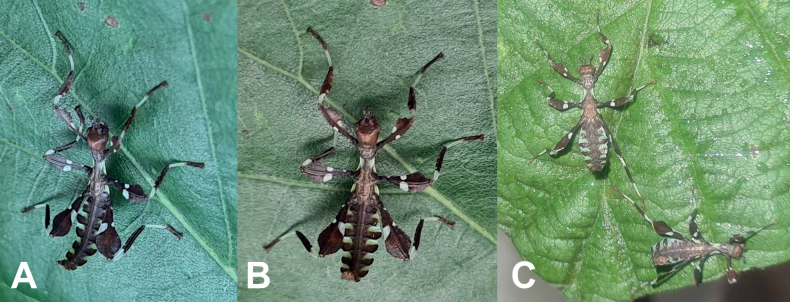
*Phylliumortizi* sp. nov. freshly hatched nymphs reared and photographed by Maxime Ortiz (France) **A** habitus, dorsolateral **B** habitus, dorsal **C** two nymphs drinking moisture off of bramble (*Rubus* sp.), one of the few times freshly hatched nymphs hold still.

Figs 8A , 10 , 11 , 12 , 13 , 14

##### Material examined.

***Holotype*** ♀: “Philippines, Mindanao, Lanao Del Sur, Wao, February 2019; Collection SLT; DNA no. 35”. Deposited in the Montreal Insectarium, Quebec, Canada (IMQC; Fig. 11D). ***Paratypes***: (5 ♀♀, 14 ♂♂, 2 ♀♀ nymphs, 99 eggs). See Suppl. material 1 for details about paratype specimens, their collection data, and depositories.

##### Differentiation.

Females are morphologically most similar to *Phylliumbilobatum* due to their small size, strongly lobed abdomen, broad profemoral exterior lobe, large triangular teeth of the profemoral interior lobe, and similar thorax spination. The antennae morphology can differentiate these species as *Phylliumortizi* sp. nov. has the terminal antennal segment notably longer (approximately the same length as the preceding two and a half or three segments; Fig. 11E) vs *Phylliumbilobatum* which has a stout terminal segment which is only approximately as long as the preceding two segments. Additionally, the tegmina venation can differentiate these species as *Phylliumortizi* sp. nov. has tegmina with the distance between the first radial (R1) split and the wing base ca 1½× greater than the distance between the first radial (R1) split and the radius to media crossvein (R–M) vs in *Phylliumbilobatum* where the distance between the first radial (R1) split and the wing base is notably greater than the distance between the first radial (R1) split and the radius to media crossvein (R–M) (ca 2½× longer). *Phylliumortizi* sp. nov. occurs within the range of *Phylliummabantai* and due to the extreme morphological variability of *Phylliummabantai*, certain strongly lobed forms of *Phylliummabantai* females can look similar to *Phylliumortizi* sp. nov. females. However, size appears to be able to consistently differentiate these two species as *Phylliumortizi* sp. nov. are smaller (66.8–76.4 mm long) vs *Phylliummabantai* which are larger (91.2–99.4 mm long; Hennemann et al. 2009). Besides size, it can be very difficult to differentiate these two species when only dealing with female specimens; however, it appears as though the spination of the prescutum sagittal plane might allow differentiation as there are typically only three tubercles in *Phylliumortizi* sp. nov. females which are systematically decreasing in size and are distinctly raised above the prescutum surface (Fig. 11B) vs *Phylliummabantai* which can have three to five blunted nodes which follow the anterior spine, and these are not as prominent.

For male *Phylliumortizi* sp. nov. they are morphologically most similar to *Phylliummabantai* and *Phylliumiyadaon* sp. nov. based upon their size, wing venison, and general lobes of the legs. The abdomen of *Phylliumortizi* sp. nov. appears to be relatively stable in shape based upon reared and wild collected specimens and within *Phylliummabantai* male abdominal shape is also rather stable despite female *Phylliummabantai* having drastically different forms. Between these two species the abdominal general shape and the size of the eye sports appears to be a reliable feature for differentiation as the *Phylliummabantai* abdomen has smooth abdominal edges (either giving the abdomen a spade shaped or slightly ovoid appearance) and abdominal segment V has small eye spots vs *Phylliumortizi* sp. nov. which has somewhat of a rectangular abdomen (with segments V and VI approximately even in width) and the margins of VI, VII, and VIII gently rounded giving the abdomen a slightly scalloped edge and the eye spots of abdominal segment V are always large taking up at least ½ of the segment length (Fig. 10D). This scalloped edge abdominal shape makes *Phylliumortizi* sp. nov. superficially appear similar to *Phylliumiyadaon* sp. nov. but these two species can be differentiated by the width of the interior protibial lobe, interior profemoral lobe, and exterior mesofemoral lobe. In *Phylliumortizi* sp. nov. the protibial interior lobe is at least 2× wider than the width of the protibial shaft (Fig. 12A) vs *Phylliumiyadaon* sp. nov. which is much narrower, at most only slightly wider than the protibial shaft width (Fig. 9C). In *Phylliumortizi* sp. nov. the protibial interior lobe is much more pronounced with a width greater than 2× the profemoral shaft width and an angle ca 90 degrees (Fig. 12A) vs *Phylliumiyadaon* sp. nov. which has a notably more slender profemoral interior lobe with a distinctly obtuse angle and a width only ca 1½× as great as the profemoral shaft width (Fig. 9C). Additionally, the exterior mesofemoral lobe allows differentiation as the lobe in *Phylliumortizi* sp. nov. is distinctly marked by serrate teeth (at least two) but in *Phylliumiyadaon* sp. nov. the exterior lobe is bare, lacking teeth.

The eggs of *Phylliumortizi* sp. nov. are rather unique as they have two general types of pinnae on their surfaces (of which all surfaces have the same types) some that are the more typical feather-like pinnae and a second type which is thinner and more filament-like, lacking projections like is seen in the feather-like pinnae. These thinner filament-like pinnae are not seen in other phylliids eggs, and the closest types are those seen in *Phylliumtobeloensebhaskarai*Cumming et al. 2019b; however, that species typically has the thinner pinnae ending in a fork or a hooked tip vs in *Phylliumortizi* sp. nov. where the tips taper off and are thin (Fig. 13). *Phylliumortizi* sp. nov. eggs also lack any “bald patches”, a feature which many species have such as *Phylliummabantai*.

Freshly hatched nymphs are only known for a handful of *Phyllium* species at present, but from what is known *Phylliumortizi* sp. nov. can be differentiated from congenerics reliably. *Phylliumortizi* sp. nov. (Figs 8A, 14) is most similar to *Phylliummabantai* (Fig. 8B) and *Phylliumsamarense* sp. nov. (Fig. 8C) due to each abdominal segment lateral lobe being split approximately in half by two colors (with the anterior ½ mint/lime green and the posterior 1/2 brown; Fig. 8A–C). All other *Phyllium* species have the abdomen rather uniform in color (brown/black) and at most only have the margins slightly marked with white or yellow. *Phylliumortizi* sp. nov. can be differentiated from *Phylliummabantai* and *Phylliumsamarense* sp. nov. based on the coloration of the meso- and metafemoral lobes. In *Phylliumortizi* sp. nov. the white stripe is broken on both the meso- and metafemora therefore giving them a somewhat large spotted appearance, vs, the other two species which have these white stripes unbroken reaching from one end of the femora to the other. Additionally, the green color on the abdomen of *Phylliumortizi* sp. nov. appears to be a bit darker and more richly green than the others which are paler and more mint green but depending on the lighting they are observed under the coloration can vary slightly.

##### Description.

**Female. *Coloration*.** Coloration description is based upon living individuals in culture (Fig. 10A, B, E). Overall coloration is lime green throughout, with certain areas slightly variable in coloration with tan, orange, or brown. To date the majority of females in captive culture have mostly been solidly green with minimal additional colored area, but consistently the mesonotum has been tan and brown in color. Within captive cultures a few individuals have expressed significant coloration variations, such as fully brown/tan (Fig. 10E). The margins of the femoral lobes and the protibial interior lobe are the most variable with darker colored individuals having most of the lobe colored (Fig. 11A) but often these features in paler individuals are only lightly highlighted in brown/orange. Compound eyes are of a tan coloration. The ventral coloration of the meso- and metacoxae are bright orange (Fig. 10B).

***Morphology*. *Head*.** Head capsule slightly longer than wide, vertex moderately wrinkled with evenly spaced, small nodes across most of the surface (most distinct on the posterior ½), with the posteromedial tubercle the most prominent feature on the head capsule, many times larger and broader than any of the nodes (Fig. 11C). Frontal convexities broad and moderately pointed, with a slightly granular surface and sparse short setae throughout. Compound eyes slightly protruding from the head capsule, not overly bulbous, taking up ca ⅖ of the head capsule lateral margins (Fig. 11C). ***Ocelli absent*.** Antennal fields ca ⅓ wider than the width of the first antennomere. ***Antennae*.** Antenna consist of nine segments, with the terminal segment approximately the same length as the preceding 2½ or 3 segments’ lengths combined (Fig. 11E). Antennomeres I–VII sparsely marked with short and thin transparent setae, the terminal two antennomeres are covered in stout, dark brown setae which are irregularly spaced (Fig. 11E). ***Thorax*.** Pronotum with gently concave anterior margin and straight lateral and posterior margins. The overall shape is an isosceles trapezoid with the anterior margin the longer side and the narrower posterior margin ca ⅗ of the anterior width (Fig. 11C). The pronotum surface is slightly wrinkled, with only a prominent pit in the center, and a distinct furrow anterior to the central pit and weakly formed posterior and lateral furrows to the pit (Fig. 11C). The pronotum has moderately formed anterior and lateral rims and a weakly formed posterior rim, all of which are smooth, lacking distinct granulation or setae (Fig. 11C). Prosternum and the anterior ⅓ of the mesosternum have a slightly wrinkled surface marked with numerous broad nodes, with the remainder of the mesosternum and the metasternum relatively smooth without notable features. Prescutum approximately as long as wide, with lateral rims marked by seven or eight medium to large tubercles with somewhat irregular spacing (Fig. 11C). Prescutum anterior rim prominent, strongly protruding as a large sagittal spine, with the rim surface slightly lumpy (Fig. 11C). Prescutum sagittal crest with two additional distinct tubercles, reducing in size steadily from the prominent anterior tubercle to a smaller posterior tubercle (Fig. 11B). Prescutum surface irregularly lumpy and slightly raised along the sagittal crest (Fig. 11B). Mesopleura begin at the anterior of the prescutum and diverge slowly on the anterior margin but immediately angle outwards prominently and then evenly diverge throughout the rest of their lengths (Fig. 11C). Mesopleuron lateral margin with six or seven medium to large tubercles with fine points and three or four smaller nodes interspersed, all situated on the anterior ⅗ with the remainder of the margin relatively smooth (Fig. 11C). Face of the mesopleuron heavily wrinkled, with two notable divots, one on the anterior margin and one near the middle (Fig. 11C). ***Wings*.** Tegmina long, reaching onto abdominal segment VIII. Tegmen venation; the subcosta (Sc) is the first vein and runs relatively straight from the base with just a slight arcing in the middle as it angles towards the margin, terminating on the margin ca ⅕ of the way through the tegmen length. The radius (R) spans the central portion of the tegmen and branches into two diverging veins; the first radius (R1) branches ca ⅕ of the way through the wing length, runs straight, and terminates on the margin slightly < ⅓ of the way through the tegmen length, and the radial sector (Rs) branches ca 2/7 of the way through the tegmen length, arcs gently as it spans through the majority of the tegmen central surface, and terminates on the margin of the distal ⅓ of the tegmen length. There is a weak continuation of the radius following the prominent Rs branching which continues on as a short and thin radius to media crossvein (R–M) that weakly connects the two veins. The media (M) is bifurcate with the media anterior (MA) branching near the middle of the tegmen length and media posterior (MP) branches near the distal ⅓ of the tegmen length with both veins arcing gently and running parallel/subparallel towards the tegmen margin with the media anterior terminating near the distal ¼ of the tegmen and the media posterior terminating near the distal ⅕ of the tegmen length. Following the branching of the media posterior there is a thin media to cubitus crossvein (M–Cu). The cubitus (Cu) is also bifurcate, branching near the posterior ¼ of the tegmen into the cubitus anterior (CuA) which runs arcing parallel with the media posterior to the tegmen apex and the cubitus posterior (CuP) which runs away from the cubitus anterior as it follows the tegmen margin and it fades near the apex. The first anal vein (1A) is simple and fuses with the cubitus early on, at the length approximately midway between the splitting of the first radial and the radial sector. Alae rudimentary, only just a nub. ***Abdomen*.** Abdominal segments II through the anterior ⅔ of segment IV uniformly diverging with straight margins. The posterior ⅓ of segment IV through segment VI are subparallel, giving the abdomen a boxy appearance as these segments only slightly converge. The posterior margin of segments V and VI are slightly broader than the anterior margin of their following segment and this difference is notably more significant in segments VII and VIII which have distinct lobes projecting slightly beyond the anterior margin of the following segment as these segments narrow notably and converge towards the abdomen apex. Segments IX and X are notably narrower than the previous segments, and have converging, straight margins to the broad rounded apex. ***Genitalia*.** Subgenital plate starts at the anterior margin of tergum VIII, is moderately broad, and the apex extends ca ⅓–½ of the way onto tergum X with margins that arc smoothly at first and then run slightly converging to the bluntly pointed apex (Fig. 11F). Gonapophyses VIII are long and moderately broad, with their tips slightly exceeding the apex of the abdominal segment X; gonapophyses IX are shorter and narrower, mostly hidden below the gonapophyses VIII (Fig. 11F). Cerci are mostly flat with only the interior margin near the middle slightly cupped, a surface that is relatively smooth with little granulation, and only the very slightly serrate terminal margins are marked with sparse, short setae (Fig. 11F). ***Legs*.** Profemoral exterior lobe reaching from end to end in a broad, rounded, and obtusely angled lobe which is only slightly wider than the interior lobe. Edge of the profemoral exterior lobe granular with two or three small teeth present on the distal margin. Profemoral interior lobe ca 2× as wide as the greatest width of the profemoral shaft, with the lobe in the shape of a right angle with the distal margin ornamented with four or five serrate or triangular teeth irregularly arranged in a two-two or three-two pattern with looping gaps between them, and typically the largest teeth are the ones in the middle. Mesofemoral exterior lobe arcs from end to end with the greatest width on the distal ⅓ and distal to this widest point are two small serrate teeth. Mesofemoral exterior lobe is slightly wider than the mesofemoral shaft width, and the interior lobe is slightly < 2× the width of the mesofemoral shaft. Mesofemoral interior lobe arcs from end to end with the greatest width on the distal ⅓ and from this greatest width to the distal end there are five serrate teeth while the thinner proximal portion of the lobe lacks dentition. Metafemoral interior lobe arcs end to end, with the distal half slightly wider than the proximal half and this distal half is marked with six to seven serrate teeth. Metafemoral exterior lobe is thin and smooth, hugging the metafemoral shaft and lacks dentition. The protibial interior lobe spans the entire length and is ca 2× the width of the protibial shaft. The lobe is roundly triangular with the widest portion slightly distal to the midline. Protibiae lacks exterior lobes, mesotibiae and metatibiae lack both exterior and interior lobes.

***Measurements of holotype female* [mm].** Length of body (including cerci and head, excluding antennae) 66.8, length/width of head 6.0/5.4, antennae (broken in the holotype), pronotum 5.9, mesonotum 6.2, length of tegmina 43.5, greatest width of abdomen 31.6, profemora 12.3, mesofemora 11.0, metafemora 13.8, protibiae 7.3, mesotibiae 8.5, metatibiae 12.1.

***Measurements of paratype females* [mm] (ex culture).** Length of body (including cerci and head, excluding antennae) 74.2–76.4, length/width of head 6.2–6.4/5.9– 6.1, antennae 4.1–4.4, pronotum 3.9–4.2, mesonotum 6.4–6.6, length of tegmina 47.8–48.2, length of alae 2.4–2.6, greatest width of abdomen 35.5–36.8, profemora 14.0–14.4, mesofemora 12.8–13.2, metafemora 15.7–16.1, protibiae 7.5–8.2, mesotibiae 8.4–8.9, metatibiae 13.7–14.1.

**Male. *Coloration*.** Coloration description is based upon living specimens in captivity (Fig. 10A, C, D). Overall coloration lime green throughout with variable patches of brown to ruddy brown coloration. These variable brown to ruddy brown areas are primarily around the margins on the lobes of the legs, the thorax, the tips of the antennae, a set of eye spots on abdominal segment V, and the margins of the abdomen. The areas that are most variable are the lobes of the pro- and meso- legs which can be fully or almost fully colored or only partially with just the margins colored. Ventral coloration of the meso- and metacoxae can be pale cream to slightly yellow, not identical to the green of their bodies (Fig. 10D).

***Morphology*. *Head*.** Head capsule approximately as long as wide, with a vertex that is slightly lumpy and marked by a few irregularly spaced nodes (Fig. 12C). Frontal convexities stout, notably short, and marked by only a few short setae. The posteromedial tubercle is not large (but it is larger than any of the nodes on the head) and it is only slightly raised from the head capsule. Compound eyes large and bulbous, occupying ca ⅖ of the head capsule lateral margins (Fig. 12C). There are three well-developed ocelli that are notably raised and located between the compound eyes (Fig. 12C). ***Antennae*.** Antennae (including the scapus and pedicellus) each consist of 23 segments, all segments except the scapus and pedicellus and terminal five segments are covered in varyingly spaced setae that vary in length (many approximately as wide as the segment or slightly shorter, others longer than the segment is wide; Fig. 12D). The terminal five segments are covered in dense, short, darkly colored setae and the scapus and pedicellus are nearly completely bare as they only have a few thin and short setae (Fig. 12D). ***Thorax*.** Pronotum with anterior margin notably concave and lateral margins slightly convex and converging to a straight posterior margin that is slightly > ½ the width of the anterior rim (Fig. 12C). Anterior and lateral margins of the pronotum have well-formed rims and the posterior margin lacks a rim (Fig. 12C). Face of the pronotum is marked by a distinct furrow and pit in the center and a relatively smooth surface with sparse, irregularly spaced granulation (Fig. 12C). Prosternum surface is heavily marked with large nodes with those on the posterior half slightly larger than those on the anterior. Almost the entire mesosternum surface is distinctly wrinkled and marked with granulation mostly along the sagittal plane. Metasternum surface with only weak wrinkles and minimal or no granulation. Prescutum approximately as long as the greatest anterior width, with lateral margins that are converging to the notably narrower posterior (Fig. 12C). Lateral rims with eight or nine tubercles, of these around five are slightly more prominent than the others (which are smaller and can vary in placement/size). Prescutum surface smooth and rises up to meet the sagittal crest which is marked by four tubercles which decrease in size from the anterior to the posterior. Prescutum anterior rim is well-formed and prominently raised into a distinct sagittal tubercle, and the surface of the rim is smooth (Fig. 12F). The mesopleura begin on the anterior prescutum margin and diverge at an increasing degree throughout their length, at first only gradually but increasingly diverging to the posterior (Fig. 12C). Mesopleuron lateral margin with five larger tubercles throughout the length with seven or eight smaller nodes spaced throughout (sometimes one or two between major tubercles) and almost all tubercles end with a seta or two. Face of the mesopleuron notably wrinkled and marked with two weak divots (one near the middle and the other near the anterior margin). ***Wings*.** Tegmina moderate length, extending ½ of the way onto abdominal segment III. Tegmen wing venation: the subcosta (Sc) is the first vein, is simple, runs through the tegmen for the first half of its length and then on the distal half it bends and runs angled towards the tegmen margin where it terminates ca ½ of the way through the overall tegmen length. The radius (R) spans the entire length of the tegmen with the first radius (R1) branching ca ⅖ of the way through the tegmen length and terminates near the distal ⅓ of the tegmen, and then the radial sector (Rs) runs straight to the wing apex. The media (M) also spans the entire length of the tegmen and runs side by side along the radius/radial sector with the media posterior (MP) branching off near the middle of the tegmen and running angled towards the apex/cubitus, and the media anterior (MA) runs straight to the tegmen apex. The cubitus (Cu) cuts across the tegmen to the margin ca ⅓ of the way through the length and runs along the edge of the tegmen. The media posterior vein fuses with the cubitus and as the cubitus reaches the apex it fades. The first anal (1A) vein terminates upon reaching the cubitus ca ⅓ of the way through the tegmen length. Alae well-developed in an oval fan configuration, long, reaching onto abdominal segments IX. Ala wing venation: the costa (C) is present along the entire foremargin giving stability to the ala. The subcosta (Sc) is long, spanning ca ½ of the ala length and is mostly fused with the radius near the base of the wing but terminates when it meets the costa. The radius (R) spans the entire wing and branches slightly < ⅓ of the way through the ala length into the first radius (R1) and radial sector (Rs) which run gently diverging for ca ½ of their length, then run parallel until they near the apex of the ala where the radial sector begins to arc towards the first radial and either weakly joins it or fades before fully fusing with it, and the first radial either weakly reaches the apex or fades before fully reaching the apex. The media (M) branches early, ca ⅛ of the way through the ala length into the media anterior (MA) and the media posterior (MP) which run gently diverging at first, then parallel for a short while, then gently converging for ca ½ their length until the distal ¼ of the wing where the media posterior fuses with the media anterior which runs fused to the apex where it terminates. The cubitus (Cu) runs unbranched and terminates at the wing apex. Of the anterior anal veins, the first anterior anal (1AA) fuses with the cubitus slightly > ⅕ of the way through the ala length and then the first anterior anal branches from the cubitus ⅗ of the way through the ala length where it uniformly diverges away from the cubitus until it terminates at the wing margin. The anterior anal veins two–seven (2AA–7AA) have a common origin and run unbranched in a folding fan pattern to the wing margin. The posterior anal veins (1PA–6PA) share a common origin separate from the anterior anal veins and run unbranched to the wing margin with slightly thinner spacing than the anterior anal veins. ***Abdomen*.** Lateral margins of abdominal segment II parallel, III slightly diverging, IV diverging more prominently for the anterior ⅔ then run parallel, V and VI parallel or nearly so with the posterior end slightly wider on each segment so the abdomen has a slight undulating appearance, VII has a rounded margin narrowing to the notably narrower VIII, VIII through X converging relatively uniformly to the rounded apex of the abdomen. ***Genitalia*.** Poculum broad and ends in a blunt apex that slightly passes the anterior margin of segment X (Fig. 12E). Cerci long and slender, extending from under the anal abdominal segment, nearly flat, surface slightly granular, and notably pubescent. Vomer broad and stout with straight sides evenly converging and ending in a singular thick apical hook (Fig. 12E). ***Legs*.** Profemoral exterior lobe arcs from end to end, hugging the profemoral shaft, with a maximum width as wide as the profemoral shaft width, and the margin on the distal half is marked with five small, finely pointed serrate teeth, with the proximal half marked with minimal granulation, and sparsely ornamented with short, fine setae throughout the lobe length (Fig. 12A). Profemoral interior lobe roundly triangular (approximately right angled) and typically marked with five sharp teeth arranged in a three-two pattern with looping gaps between them, with the central two teeth often larger than the others (Fig. 12A). Mesofemoral exterior lobe is almost entirely situated on the distal half with the proximal half thin to almost fully reduced. Mesofemoral exterior lobe has its greatest width on the distal end and is approximately the same width as the greatest mesofemoral shaft width. The distal ⅓ of the mesofemoral exterior lobe is marked with two or three sharply pointed serrate teeth. Mesofemoral interior lobe has a similar shape as the exterior lobe (very thin on the proximal ½ and widest on the distal ½) but the distal ⅖ is marked with 6–8 sharply pointed serrate teeth. Metafemoral exterior lobe lacks dentition and has a straight margin hugging the metafemoral shaft. Metafemoral interior lobe arcs end to end with the distal ½ wider than the proximal ½, and the distal ½ margin is marked with seven or eight strongly angled serrate teeth. Protibiae lacking exterior lobe, interior lobe reaching end to end in a broad arc with the proximal and distal ends notably tapered and the center the widest point which at its maximum is slightly > 2× the width of the protibial shaft (Fig. 12A). Meso- and metatibiae simple, lacking lobes completely.

***Measurements of paratype males* [mm] (ex culture).** Length of body (including cerci and head, excluding antennae) 53.5–55.8, length/width of head 2.9–3.0/2.8– 2.9, antennae 2.6.1–27.9, pronotum 2.3–2.4, mesonotum 2.8–2.9, length of tegmina 17.6–19.0, length of alae 39.7–41.2, greatest width of abdomen 12.3–12.9, profemora 8.7–9.4, mesofemora 8.9–9.3, metafemora 10.4–10.8, protibiae 5.8–6.2, mesotibiae 5.3–6.0, metatibiae 7.8–7.9.

**Eggs.** (Fig. 13). The overall color is brown, with the capsule surface lighter in color and the pinnae darker. The lateral surfaces are nearly flat (only slightly convex), with the posterior of the egg slightly wider than the anterior. There are two types of pinnae on the capsule surfaces; moderate to long feather-like pinnae which are found along the operculum rim and along the capsule margins (these feather-like pinnae are not as well formed as in other species with large feather-like pinnae and instead these appear more like a “wet feather” with the lateral frills less defined) and an addition pinna type which are short to medium length “filament-like” pinnae which lack lateral frills and are instead a simple stock without elaborate expansions. The lateral surfaces of the capsule are densely marked throughout with short to medium length filament-like pinnae emerging from a roughly textured surface (Fig. 13A). The dorsal surface has the micropylar plate (which is marked with a distinct margin) and the plate spans approximately the central ⅗ of the capsule length with a shape that is distinctly tear-shaped with the wide, rounded end around the micropylar cup which is located on the posterior ⅖ of the capsule (Fig. 13C). On either side of the micropylar plate is a similar surface texture/pinnae arrangement to the lateral surfaces. The operculum is slightly ovular, the surface is flat and marked moderately by filament-like pinnae, and the outer margin is rimmed with medium length feather-like pinnae (Fig. 13D). The ventral surface of the egg capsule has a surface texture and pinnae similar to the lateral surfaces (Fig. 13F).

***Measurements including the extended pinnae* [mm].** Length (including operculum): 4.5–4.6; maximum width of capsule when viewed from lateral aspect 2.9–3.0; length of micropylar plate 2.2–2.3.

##### Newly hatched nymphs.

(Fig. 14). The general color throughout the body is a rich dark brown. The basitarsi are cream colored and the remaining tarsal segments are dark brown. All tibiae lack exterior lobes but the protibiae do have a smoothly arcing interior lobe which is approximately as wide as the protibial shaft width and the meso- and metatibiae have very thin interior lobes only approximately as wide as the shaft they are on. All tibiae have a similar color pattern with the proximal ¼–⅓ pure white and the remainder brown with sparse white/tan partial striping (less so on the metatibiae and the most prominent on the protibiae). All femoral lobes are similar in color pattern with their primary color brown and near the middle (of the profemora) or the proximal ⅖ (of the meso- and metafemora) there are distinct white spots on each side of the femoral shaft that are crisply colored and not connected across the shaft. Additionally, on the meso- and metafemoral interior lobes at the base are small single white patches. All femoral interior lobes have mild serration and the meso- and metafemoral exterior lobes have dulled dentition on their distal ends. The distal end of the metafemoral exterior lobe also has a faint white edge. The head and thorax base color are brown, and the mesothorax and metathorax margins are marked with lime green. The abdomen base color is brown, with the centerline of the abdomen where the internal organs are fully brown but the thin lobes on each side of this center are bicolored. Each segment is marked on the anterior ½–⅓ with green and the remainder of the segment is brown, except for segments VIII–X that are mostly brown and only marked minimally on the lateral margin with green. The abdomen’s general shape is long and narrow, with a maximum width < ½ of the abdomen length. The widest point of the abdomen is abdominal segment IV.

##### Etymology.

Patronym. Named after Maxime Ortiz (France) who is a well-known phasmid breeder who established this new species in culture and who shared type material with the authors for this study. Thanks to his efforts we were able to understand the morphological diversity within the males, females, freshly hatched nymphs, and eggs.

##### Distribution.

At present this species is known from four provinces in Mindanao; Lanao Del Sur Province (with the exact type locality of Wao Municipality), South Cotabato Province, Davao del Sur Province, and from the inexact locality of Bukidnon Province from captive reared specimens.

##### Remarks.

This species was first thought to represent a small, strongly lobed *Phylliummabantai* or possibly the first record of *Phylliumbilobatum* Gray, 1843 in more than a century, a species with which *Phylliumortizi* sp. nov. shares many general similarities. However, thanks to the rearing of additional specimens by Maxime Ortiz (France), the intraspecific variation was revealed, and the fine details instead reliably differentiate these species. *Phylliumortizi* sp. nov. was sampled within Bank et al. (2021) as “*Phyllium* sp. 8 ‘Kitanglad’” where it was recovered as sister species to *Phylliumsamarense* sp. nov. from Samar Island (“*Phyllium* sp. 3 ‘North Samar’” within Bank et al. (2021)). The same tree topology was recovered within our included phylogenetic analysis (Fig. 2).

The eggs of *Phylliumortizi* sp. nov. are primarily covered with pinnae “type 5” (feather-like) as defined by Büscher et al. (2023). In addition to this type of pinnae, however, is a pinnae type not characterized within Büscher et al. (2023). Intermixed within the pinnae “type 5” are filament-like pinnae, thin tapering pinnae without significant side branches or prominent splitting at the apex (Fig. 13). Morphologically these filament-like pinnae appear to be simplified “type 5” pinnae, not a wholly unique type worth characterizing as an independent state, particularly in light of the addition of “type 5” pinnae on the egg which logically fits within the results of Büscher et al. (2023).

#### ﻿ Pulchriphyllium

Taxon classificationAnimaliaPhasmatodeaPhylliidae

Griffini, 1898

DD2C1EF3-623E-5EF1-B5F2-CEE4F14EE728

##### Type species.

*Pulchriphylliumpulchrifolium* (Audinet-Serville, 1838).

**Figure 15.**
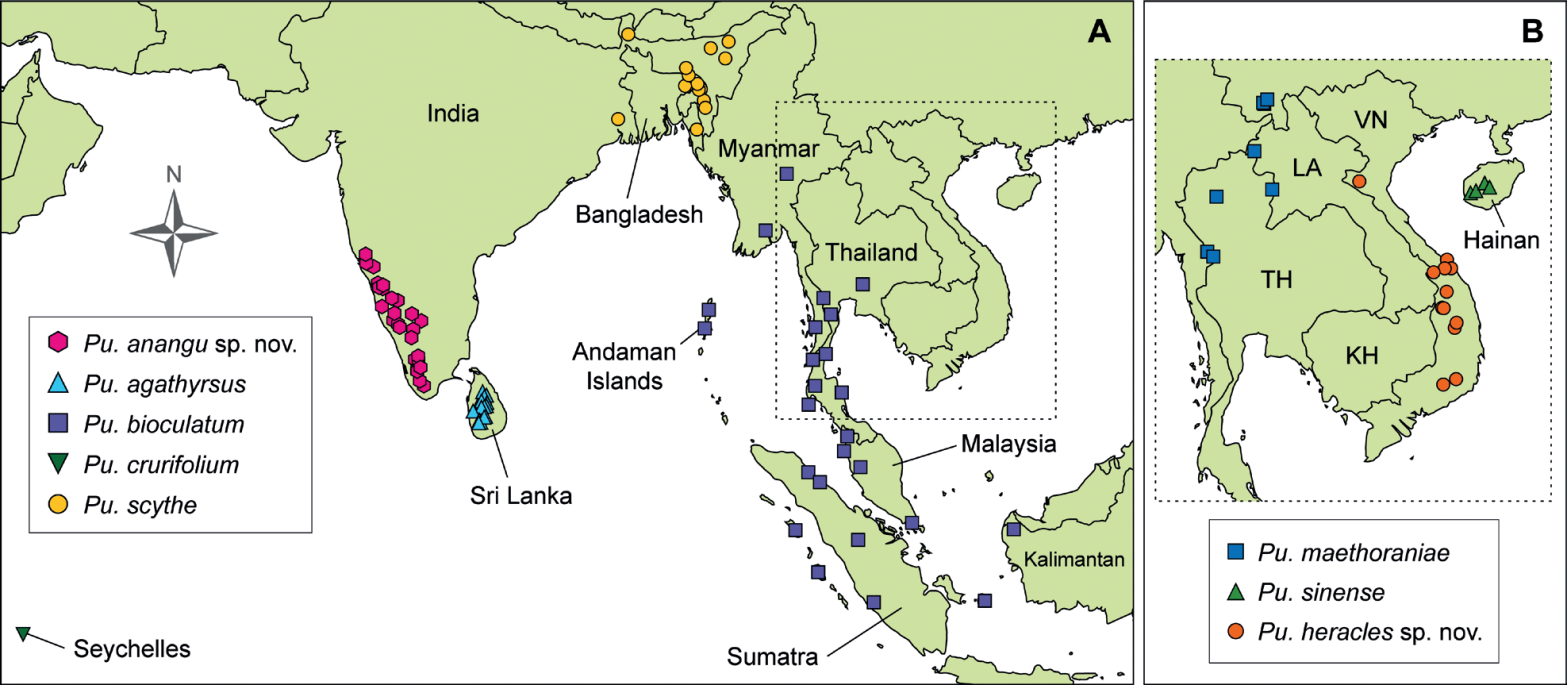
Distribution map for the herein discussed *Pulchriphyllium* species with known records plotted. For data on the distribution points used for this map, see Suppl. material 1**A** distribution of the “*bioculatum*-like species” **B** distribution of the *Pulchriphyllium* species from mainland Asia with a boxy abdominal shape (inset outline in **A**). Abbreviations: KH, Cambodia; LA, Laos; TH, Thailand; VN, Vietnam.

Historically, many *Pulchriphyllium* populations have been the subject of repeated taxonomic adjustments, oftentimes without being properly assessed or clearly defined. Originally, many of the populations were treated as distinct species, but eventually, as phylliids became better known and more common in museums, authors started to assume phylliids were species-poor with wide geographic ranges (Brock 1999; Größer 2008; Brock et al. 2022). Several taxa have been treated as forms, subspecies, or synonyms by past authors (Giglio-Tos 1910; Brock 1995; Hennemann et al. 2009), and it was only recently that authors have begun to split certain lineages into distinct species again (Seow-Choen 2017). The *Pulchriphyllium* are only found Northwest of Wallace’s line of faunal balance (Huxley 1868) and range as far north as Bangladesh/northeastern India, and as far west as the Seychelles islands (Fig. 15A). The area with the highest *Pulchriphyllium* diversity appears to be the biogeographical region of Sundaland, an area which was recovered as the likely geographic ancestral range for this clade (Bank et al. 2021).

Within the *Pulchriphyllium* there appear to be several recurring, leaf-shape mimicry patterns; broad leaf (like in *Pulchriphylliumgiganteum*), boxy leaf (like in *Pulchriphylliumpulchrifolium*), and tapered leaf (like in *Pulchriphylliumbioculatum* Gray, 1832). Of particular difficulty to differentiate are the numerous species where females have a tapered abdominal shape and males have rounded/ovoid abdomen, herein referred to as “*bioculatum*-like species”. These “*bioculatum*-like species” are a subset of the “*bioculatum* species group”, the only species group within the *Pulchriphyllium* proposed by Hennemann et al. (2009) which has not been taxonomically adjusted (the other three species groups proposed within that work have either been transferred to a different genus (such as the “*frondosum* species group” which were transferred to the *Nanophyllium* by Cumming et al. 2020d), or have since been described as their own genera (like the “*brevipenne* species group” which is now *Acentetaphyllium* Cumming & Le Tirant, 2022 and the “*schultzei* species group” which is now *Rakaphyllium* Cumming & Le Tirant, 2022).

The “*bioculatum*-like species” are difficult to morphologically differentiate, have wide-ranging geographic distributions (Fig. 15A), and coupled with the fact that the holotype of *Pulchriphylliumbioculatum* lacks a precise collection locality, has made this group of similar-looking species difficult to clearly define. Based upon the type specimen morphology, the population considered true “*bioculatum*” is the species centered around Sundaland and mainland Asia (Fig. 15A; Hennemann et al. 2009). This leaves the numerous populations once considered subspecies or synonyms of *bioculatum* (but now recognized as independent species; Fig. 15A) in need of clarification. Thanks to extensive review of historic type specimen morphology, modern specimen morphology, geographic distribution, and molecular analyses, the “*bioculatum*-like species” are now beginning to be clarified.

#### ﻿ Pulchriphyllium scythe

Taxon classificationAnimaliaPhasmatodeaPhylliidae

(Gray, 1843) stat. rev., comb. nov.

1DBF40AA-AFB2-57B4-BED0-AAF3BC428082

**Figure 16.**
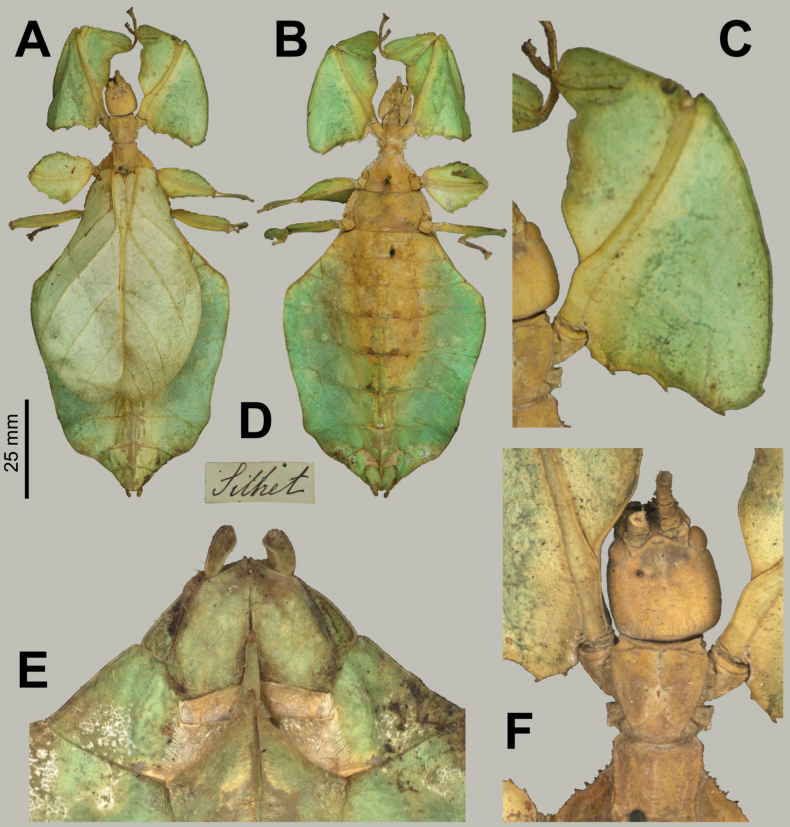
*Pulchriphylliumscythe* (Gray, 1843) stat. rev., comb. nov. herein designated lectotype female, photographs by Robert Douglas (OUMNH) **A** habitus, dorsal **B** habitus, ventral **C** detail of the front leg, dorsal **D** data label **E** detail of the genitalia, ventral **F** detail of the antenna, head, and anterior of the thorax, dorsal.

**Figure 17.**
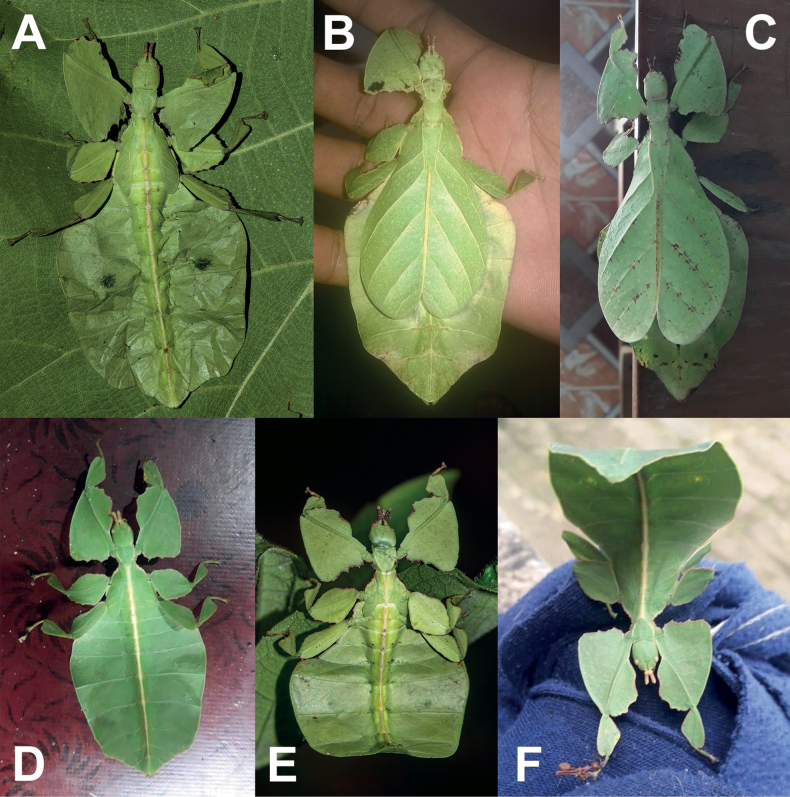
Live females of *Pulchriphylliumscythe* (Gray, 1843) stat. rev., comb. nov. **A** adult female, dorsal habitus, found near Makunda Christian Hospital, Karimganj District, Assam, 20 April 2016, photographed by Vijay Anand Ismavel (India), (https://www.inaturalist.org/observations/4311333) **B** adult female, dorsal habitus, found in Assam 788727, India, 27 December 2017, photographed by iNaturalist user @lovelymonlamin (https://www.inaturalist.org/observations/30552832) **C** adult female, dorsal habitus, found in Assam 788727, India, 27 August 2019, photographed by Dwithun Moshahary, (https://www.inaturalist.org/observations/51076352) **D** female nymph, dorsal habitus, found in Vaisam, Damchhara R. F., Tripura, India, 12 August 2020, photographed by Michael Lalruatfela (India) **E** female nymph, dorsal habitus, found near Makunda Christian Hospital, Karimganj District, Assam, 9 June 2019, photographed by Vijay Anand Ismavel (India), (https://www.inaturalist.org/observations/29119549) **F** same individual as in D but dorso-anterior view. Photographs used were uploaded to iNaturalist and are here used under license (CC BY-NC 4.0) or with explicit permission by the photographer.

**Figure 18.**
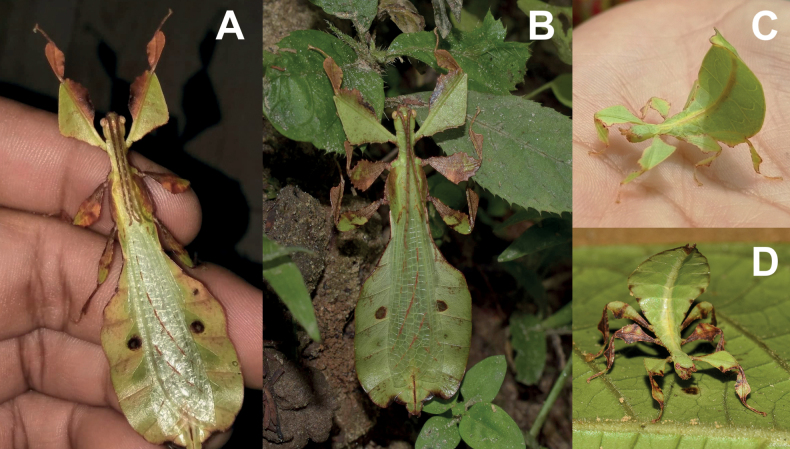
Live males and nymphs of *Pulchriphylliumscythe* (Gray, 1843) stat. rev., comb. nov. **A** male habitus, dorsal, found in Vaisam, Damchhara R. F., Tripura, India, 30 June 2019 and photographed by Michael Lalruatfela (India) **B** male habitus, dorsal, found in Badshahi Forest, Assam, India, 18 September 2020 and photographed by Rejoice Gassah (India) (https://www.inaturalist.org/observations/64092851) **C** nymph, dorso lateral view, found in Lawachara National Park, 31 July 2019, photographed by Hassan-al Razi Chayan (Bangladesh) (https://www.inaturalist.org/observations/103359437) **D** nymph, dorso-anterior view, found in Dosdewa Khasi Village, Katamoni, Assam, India, 24 April 2021 and photographed by Rejoice Gassah (India) (https://www.inaturalist.org/observations/79254856). Photographs used were uploaded to iNaturalist and here used under license (CC BY-NC 4.0) or with explicit permission by the photographer.

Figs 16 , 17 , 18 , 21C

##### Material examined.

(♀): “Silhet” (OUMNH; Fig. 16), herein designated as the lectotype. See Suppl. material 1 for additional non-type specimens reviewed.

##### Differentiation.

Presently, *Pulchriphylliumscythe* stat. rev., comb. nov. is one of the species we lack molecular data for, but thankfully there are consistent morphological differences which allow differentiation from congenerics. At the moment, the morphology of the freshly hatched nymph and the egg are not known, which would likely yield additional features for differentiation. The female *Pulchriphylliumscythe* stat. rev., comb. nov. are the easiest to consistently differentiate, and when coupled with their geographic isolation, are the reason why we are reinstating this population as a valid species.

From all other *bioculatum*-like species, *Pulchriphylliumscythe* stat. rev., comb. nov. females can be differentiated by their notably larger size (10.8 to 11.0 cm (vs the other *bioculatum*-like species which range from 6.7 to 9.5 cm long). Females of *Pulchriphylliumscythe* stat. rev., comb. nov. are so large they actually fall within the size range of *Pulchriphylliumgiganteum* (Hausleithner, 1984) (but can easily be differentiated by the mesopleural spination as *Pulchriphylliumscythe* stat. rev., comb. nov. does not have the notable medial projection like in *Pulchriphylliumgiganteum*). Male *Pulchriphylliumscythe* stat. rev., comb. nov. are poorly known (few males have been located in museum collections and few living observations have been located, coupled with the original male syntype specimen being lost), therefore, differences have been difficult to identify as consistent. At the moment the most notable feature we have been able to identify is that the profemoral exterior lobe appears to be slightly narrower than in most *bioculatum*-like species, giving the exterior lobe a more obtuse angle appearance.

##### Distribution.

Thanks to iNaturalist (Figs 17, 18) and review of museum collections, the distribution for this species is beginning to be clarified, as well as the geographic disconnect from the other “bioculatum”-like species adjacent to this species range. At the moment we are aware of records for *Pulchriphylliumscythe* stat. rev., comb. nov. from India (provinces of Bengale Occidental, Assam, Nagaland, Meghalaya, Tripura, and Mizoram) and from the Slyhet division of Bangladesh (Fig. 15A). This species is geographically separated by the drier Eastern Ghats, Deccan Plateau, and the Central Highlands from *Pulchriphylliumanangu* sp. nov. (which is found in the heavily forested and wetter southwestern India; Fig. 15A). The geographic separation from *Pulchriphylliumbioculatum* from southern mainland Asia is not as clearly defined as there are some potential “bioculatum-like” records from central Myanmar which would be useful to compare in a molecular phylogeny one day. A potential geographic barrier between the northern extreme of *Pulchriphylliumbioculatum* and *Pulchriphylliumscythe* stat. rev., comb. nov. is the high elevation Rakhine Mountains (Burmese: Rakhine Yoma).

##### Remarks.

The only remaining syntype female (Fig. 16) is herein designated as the lectotype. In the original description male and female morphology was described, suggesting that there were syntypes of both sexes (although the exact number of which were not recorded). Unfortunately, male specimens upon which the description was based could not be located within the OUMNH collection. If any male specimens or additional female specimens can ever be located, they should be considered paralectotypes.

*Pulchriphylliumscythe* stat. rev., comb. nov. is an uncommonly recorded taxon, which has mostly been recognized as a valid species by some authors, and as a synonym or subspecies/form by others (Brock et al. 2022). Some of this confusion likely is due to the lack of examination of the type material, which has never been illustrated to date, and it appears that this taxon has not been extensively reviewed and compared with other populations by past authors. As the types have not yet been illustrated, they first had to be identified within the OUMNH collection (which received the F. W. Hope collection which the type specimens were deposited within (Gray 1843)). Near one large female specimen was a label which reads “Possibly a ♀ Syntype of *Phylliumscythe* Gray. Only specimen with exactly correct size and locality data. C. O’Toole 13-xii-1979”. We agree with this assessment by C. O’Toole and based upon the style of the label (Fig. 16D) and the pin used for this specimen, this appears to match the era correctly. Unfortunately, even though at least one male syntype was described and noted by Gray (1843) as being within the same collection, a specimen could not be located which might be an original male. Unfortunately, any male specimens appear to be lost, but thanks to the remaining female specimen, to help remove uncertainty surrounding this species, we herein designate the female as the lectotype specimen for *Pulchriphylliumscythe* stat. rev., comb. nov. (Fig. 16).

#### ﻿ Pulchriphyllium crurifolium

Taxon classificationAnimaliaPhasmatodeaPhylliidae

(Audinet-Serville, 1838) stat. rev., comb. nov.

4CF362AA-9297-5F60-BBCF-966E1B08C2A8

**Figure 19.**
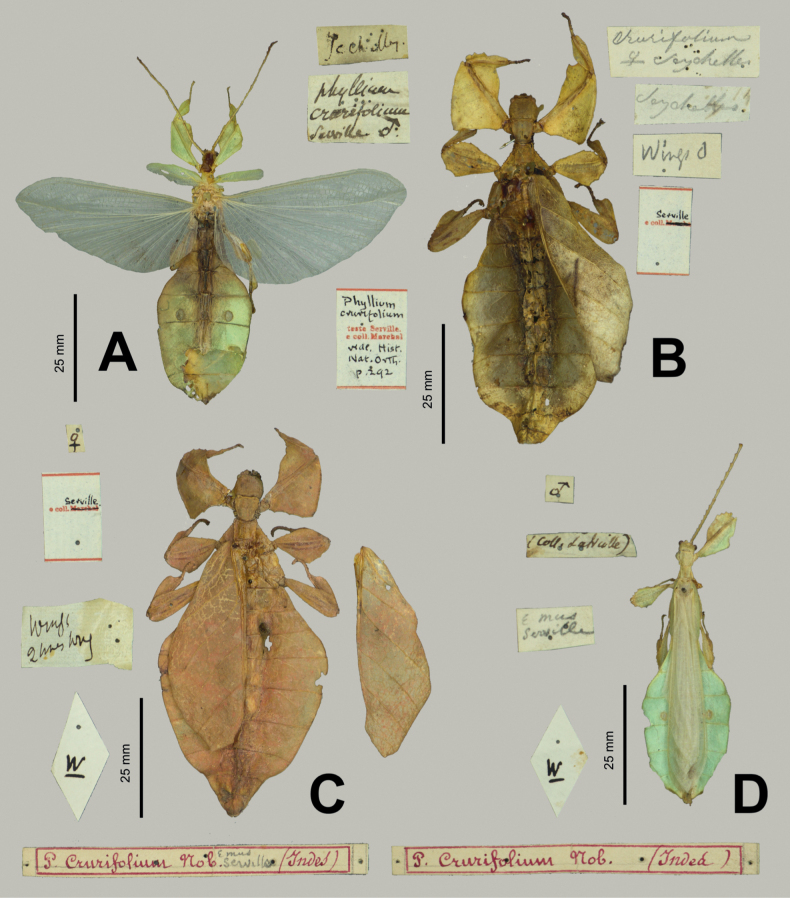
*Pulchriphylliumcrurifolium* stat. rev., comb. nov. specimens which, based upon their labels, appear to be the syntype specimens designated by Audinet-Serville in 1838 **A** paralectotype, male, dorsal habitus, associated data labels to the right **B** lectotype, female, dorsal habitus, associated data labels to the right **C** paralectotype, female, dorsal habitus, associated data labels to the left and below **D** paralectotype, male, dorsal habitus, associated data labels to the left and below. Photographs by Robert Douglas, courtesy of Oxford University Museum of Natural History.

**Figure 20.**
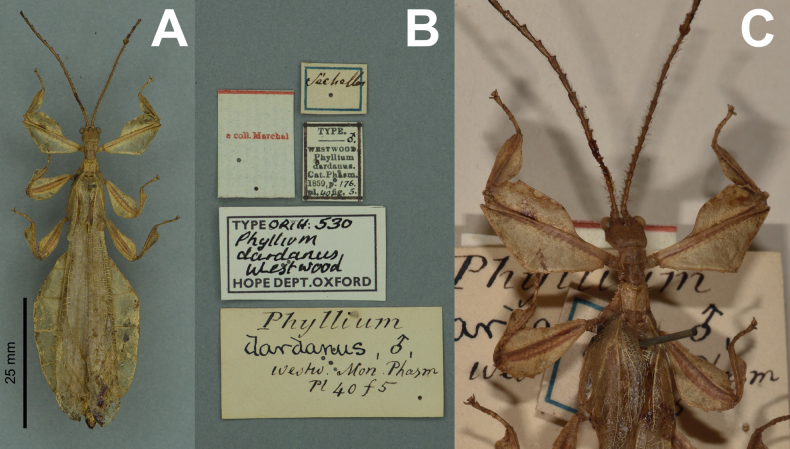
*Phylliumdardanus* Westwood, 1859 holotype male (= *Pulchriphylliumcrurifolium* stat. rev., comb. nov.) **A** dorsal habitus **B** holotype data labels **C** details of the antennae, front legs, head, and thorax, dorsal **A, B** photographs by Robert Douglas, courtesy of Oxford University Museum of Natural History **C** photograph by Paul Brock (NHMUK).

**Figure 21.**
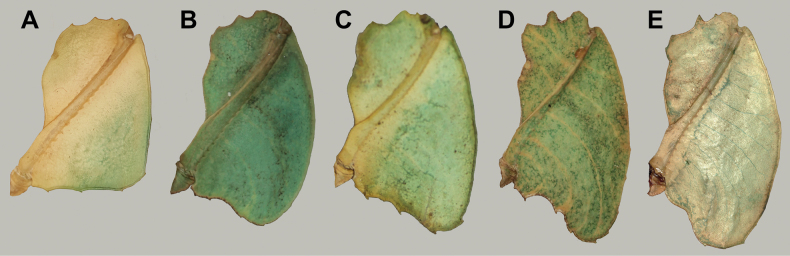
Adult female profemoral lobes from various *Pulchriphyllium* species for comparison **A***Pulchriphylliumcrurifolium* stat. rev., comb. nov. (NHMUK) **B***Pulchriphylliumbioculatum* from West Malaysia (Coll RC 16-038) **C***Pulchriphylliumscythe* stat. rev., comb. nov. from Sylhet, Bangladesh (OUMNH) **D***Pulchriphylliumpulcrifolum* from Java, Indonesia (Coll RC 16-024) **E***Pulchriphylliumbhaskarai* sp. nov. from Java, Indonesia (MZPW).

**Figure 22.**
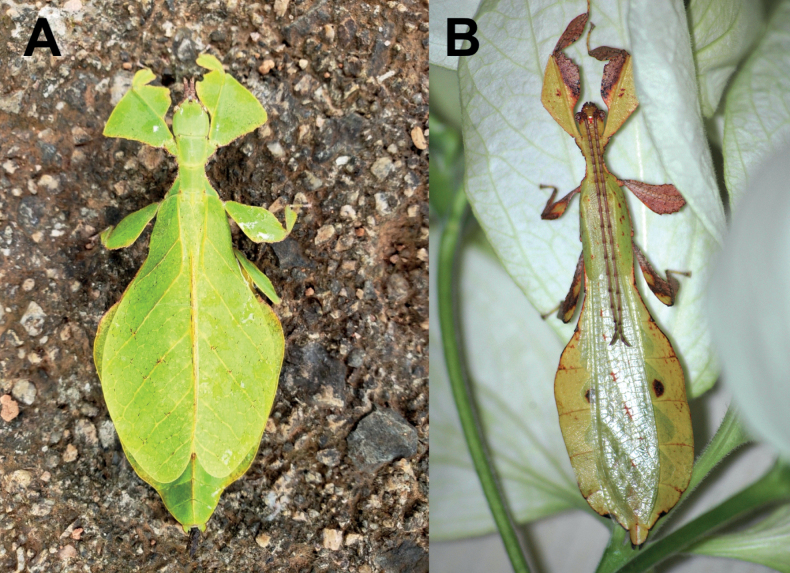
Live *Pulchriphylliumcrurifolium* stat. rev., comb. nov. **A** adult female, dorsal habitus, observed September 2019 at Sans Souci, Mahé, Seychelles, by Juan Jose Areso uploaded by iNaturalist user @liahg (Amalia Herrera Grau) (https://www.inaturalist.org/observations/76082181) **B** adult male, dorsal habitus, observed December 2006 at Grand’ Anse, Mahé, Seychelles by iNaturalist user @thierrycordenos (https://www.inaturalist.org/observations/22992534). Both images uploaded to iNaturalist and used under license (CC BY-NC 4.0).

**Figure 23.**
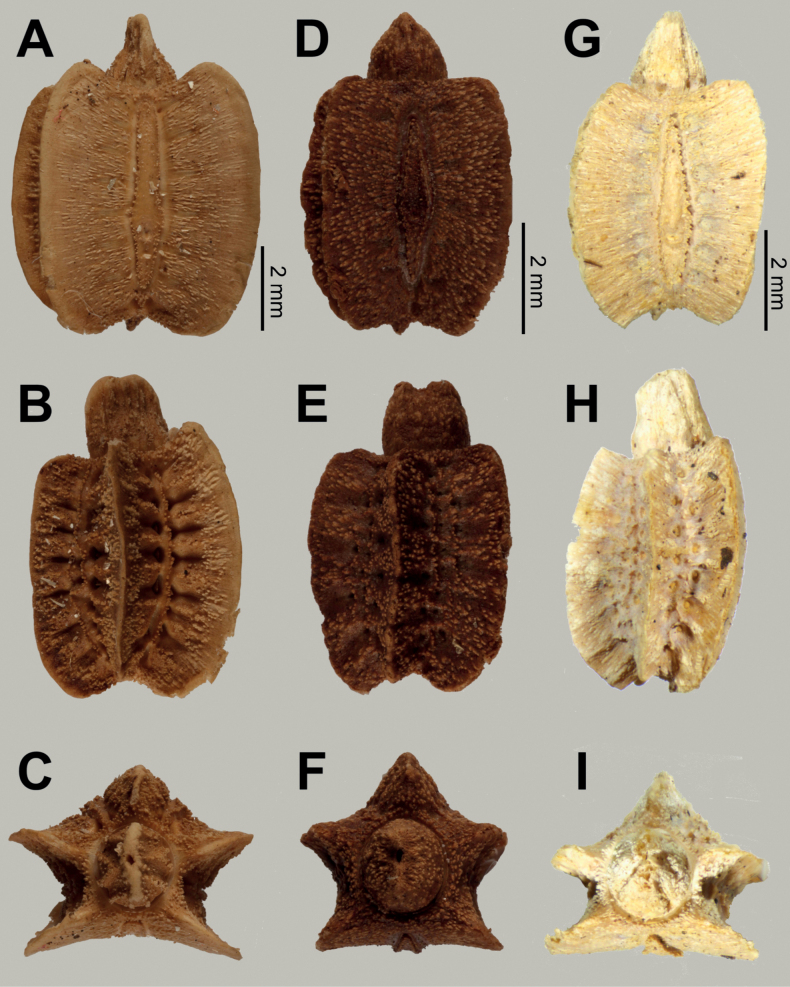
Eggs from *Pulchriphylliumbioculatum*-like species, where the eggs are known **A–C***Pulchriphylliumbioculatum* from West Malaysia (Coll RC #18-042) **D–F***Pulchriphylliumcrurifolium* stat. rev., comb. nov. from the Seychelles (Coll RC #18-233) **G–I***Pulchriphylliumagathyrsus* from Sri Lanka (Coll RC #18-232). Top row, dorsal view. Middle row, lateral view. Bottom row, anterior view.

Figs 19 , 20 , 21A , 22 , 23D–F

##### Type material examined.

(2♀♀, 2♂♂; OUMNH).

***Lectotype designation***: 1♀: “crurifolium ♀ Seychelles; Seychelles; Wings O; e coll. Serville” (Fig. 19B; OUMNH).

***Paralectotype designations***: 1♂: “Seychelles; *phylliumcrurifolium* Serville ♂; *Phylliumcrurifolium*, teste Serville. E coll. Marchal, Mae. Hist. Nat. Orth. p. 292” (Fig. 19A; OUMNH); 1♂: “♂; (coll. Latreille); E. Mus Serville; W; P. Crurifoilum Nob. (Indes)” (Fig. 19D; OUMNH); 1♀: “♀; e coll. Serville.; Wings R wings wny; W; P. Crurifoilum Nob. (Indes)” (Fig. 19C; OUMNH).

##### Additional material examined.

“*Phylliumdardanus*” ***Holotype*** (1♂; Fig. 20): “Seychelles; e coll. Marchal; TYPE ♂ WESTWOOD *Phylliumdardanus*. Cat. Phasm. 1859, p. 176. pl. 40 fig. 5.; TYPE Orth: 530 *Phylliumdardanus* Westwood HOPE DEPT. OXFORD; *Phylliumdardanus*, ♂, West. Mon. Phasm pl 40 f 5”. See Suppl. material 1 for additional non-type specimens examined.

### ﻿Synonyms

#### ﻿ Phyllium dardanus

Taxon classificationAnimaliaPhasmatodeaPhylliidae

Westwood, 1859 (OUMNH; Fig. 20)

040E6A79-D413-5DD4-81D4-2BF39902D1B7

Phyllium gelonus Gray, 1843 (NHMUK; lost according to Brock et al. 2022).

##### Differentiation.

**Female***Pulchriphylliumcrurifolium* stat. rev., comb. nov. are the easiest to differentiate from the other “bioculatum”-like species by comparing the profemoral exterior lobe shape. In *Pulchriphylliumcrurifolium* stat. rev., comb. nov. the anterior margin of the exterior profemoral lobe is nearly straight, giving the lobe a boxy appearance (Fig. 21A) vs the other “bioculatum”-like species which have this margin notably rounded, giving the lobe a slight recurve appearance (Fig. 21B).

**Males** are difficult to differentiate, and even after reviewing numerous specimens in series there appears to be enough intraspecific variation to make reliable differentiation impossible. On average males tend to have slightly narrower profemoral exterior lobes with a strongly obtuse angle (Fig. 22B), but some of the other “*bioculatum*”-like species can sometimes exhibit a similar lobe shape.

**Eggs** of *Pulchriphylliumcrurifolium* stat. rev., comb. nov. are slightly smaller than the other “*bioculatum*”-like species and have a great deal more raised nodes throughout the surfaces (Fig. 23D–F).

To date, the authors have yet to see an observation of a freshly hatched nymph of this taxon. With how unique and species-specific congenerics are (Fig. 8), it is likely that this species can be additionally differentiated by the freshly hatched nymph habitus.

##### Distribution.

(Fig. 15A) At present known from three of the islands of the Seychelles: Mahé, Praslin, and La Digue (see Suppl. material 1 for list of observations).

##### Remarks.

This taxon was originally described as a unique species from the Seychelles (Audinet-Serville 1838); however, over the decades this taxon has been treated either as a synonym, subspecies, or local form of “bioculatum” (Giglio-Tos 1910; Brock 1995; Hennemann et al. 2009). At present molecular data are lacking for this taxon, but there are numerous specimens in historic collections and recent citizen science observations (Fig. 22) which illustrate a consistent and unique morphology. Biogeographically it is likely that this species is sister to the Indian or Sri Lankan species as other organisms are known to have utilized the Maldives archipelago from the Indian sub-continent at some point during low sea levels of the past to colonize the Seychelles (O’Brien 2011; Bernardes et al. 2021).

In the decades following the description of *Pulchriphylliumcrurifolium* stat. rev., comb. nov., there were several other taxa described from the Seychelles, but since their description they have been synonymized with *Pulchriphylliumbioculatum* (Brock et al. 2022). Of the three taxa described from the Seychelles (*Phylliumcrurifolium*, *Phylliumdardanus*, and *Phylliumgelonus*), only type specimens of *Phylliumcrurifolium* and *Phylliumdardanus* remain (the type of *Phylliumgelonus* is reported as lost according to Brock et al. 2022). Thankfully, the syntype series of *Pulchriphylliumcrurifolium* stat. rev., comb. nov. (the senior synonym) has survived within the OUMNH collection. Within the original description, the syntypes are noted as: “East Indies. The male labeled from the Seychelles Islands by Latreille. Collection of M. le comte Dejean. I have both sexes” (Audinet-Serville 1838). Of these specimens, the male from the Latrielle collection (Fig. 19D), and three specimens labeled as from Serville (Fig. 19A–C) have been traced within the OUMNH collection. Unfortunately, any syntypes which were within the “Collection of M. le comte Dejean” (Pierre François Marie Auguste Dejean; 1780–1845) could not be traced. In choosing the specimen to be designated as the lectotype, we chose the specimen in question following several lines of thought. First, we wish to designate a female specimen as males are morphologically indistinguishable from several congenerics. Second, following chapter 16, article 74E of the ICZN Code (ICZN 1999) we chose the female specimen with the explicit collection data of “Seychelles” (Fig. 19B).

The eggs of *Pulchriphylliumcrurifolium* stat. rev., comb. nov. are very similar to most *Pulchriphyllium* species therefore this species adds credibility to the phylogeny of Büscher et al. (2023). Within Büscher et al. (2023) the *Pulchriphyllium* are primarily characterized as “type 7” eggs (defined as having “exochorionic structures fused to dense fins”). The only *Pulchriphyllium* species which lacks this type is *Pulchriphylliumgiganteum* which is sister species to all other *Pulchriphyllium*. Even though *Pulchriphylliumcrurifolium* stat. rev., comb. nov. could not be included within our phylogeny (Fig. 2), based upon adult and egg morphology this species likely is more closely related to one of the other “*bioculatum*-like” species.

#### ﻿ Pulchriphyllium heracles

Taxon classificationAnimaliaPhasmatodeaPhylliidae

sp. nov.

7BFFC75B-3038-52B1-BDED-A56E54F47B0C

https://zoobank.org/67CE5AF8-8489-49B4-B75B-C36DF041E763

Fig. 24

##### Material examined.

**Figure 24.**
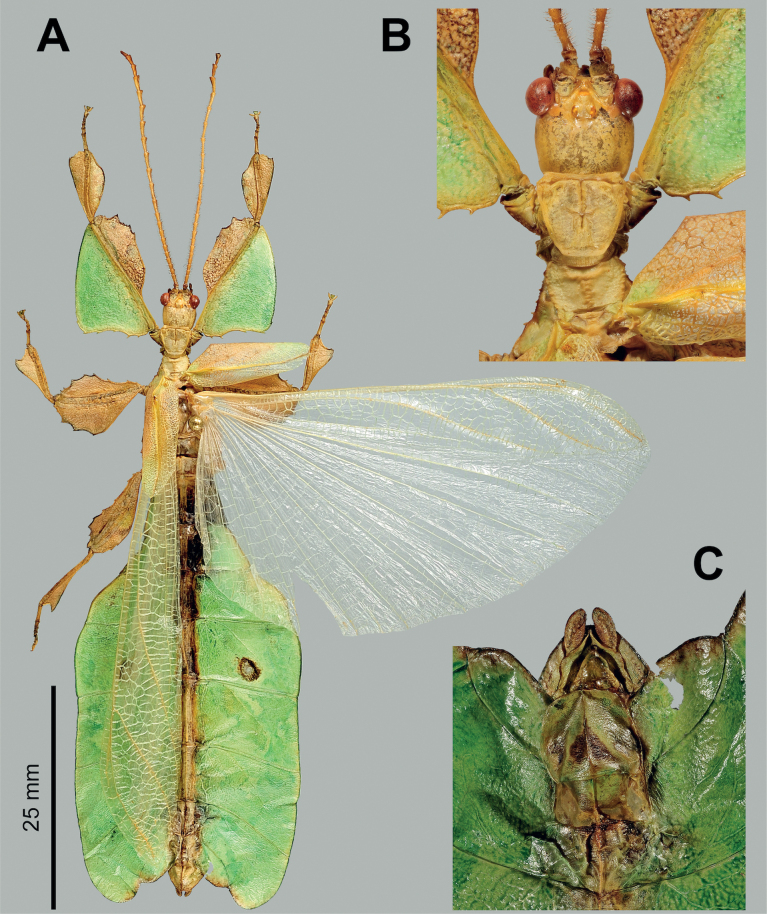
*Pulchriphylliumheracles* sp. nov. holotype male (Coll RC 16-007; IMQC) **A** habitus, dorsal **B** details of the head through thorax, dorsal **C** genitalia, ventral. Photographs by Steve Thurston (AMNH) using Microptics-USA/Visionary Digital photomicrographic system developed by Roy Larimer; using a Nikon D300 DSLR camera, Infiniti optics, and a Micro Nikkor 60 mm lens. Multiple layers stacked using Helicon Focus.

***Holotype*** ♂: “Vietnam: Da Nang Province, Ba Na Mt. 1,450 m. elv. May 2015; Coll RC 16-007” (Fig. 24). Deposited in the Montreal Insectarium, Quebec, Canada (IMQC). ***Paratypes***: (37♂♂) See Suppl. material 1 for details about paratype specimens, their collection data, and depositories.

##### Differentiation.

**Female**, freshly hatched nymph, and egg unknown. **Males** are morphologically most similar to *Pulchriphylliummaethoraniae* (Delfossee, 2015) and *Pulchriphylliumsinense* (Liu, 1990). These three species are difficult to morphologically differentiate, especially because *Pulchriphylliummaethoraniae* and *Pulchriphylliumsinense* males are only known from a few specimens, therefore the intraspecific variation is not well understood. On average it appears the mainland species (*Pulchriphylliummaethoraniae* and *Pulchriphylliumheracles* sp. nov.) are slightly larger than *Pulchriphylliumsinense* from Hainan Island, but that could simply be due to our limited knowledge of *Pulchriphylliumsinense* variation. One morphological feature which does appear to be consistent for differentiating *Pulchriphylliumheracles* sp. nov. from the other two is the interior lobe of the mesofemora. In *Pulchriphylliumheracles* sp. nov. the lobe is slightly thinner, with a maximum width of ca 1 ¼ the width of the mesofemoral shaft, whereas in the other two species this lobe is broader, ca 1½× as wide or wider.

##### Description.

**Male. *Coloration*.** Coloration description based on the dried type specimens, but despite slight discoloration from the preservation process, the natural patterns appear rather consistent. The coloration throughout is pale green with brown highlights/markings (Fig. 24). The antennae, head, thorax, and tegmina are typically yellow/tan but in life were probably green or tan. The lobes of the legs and the abdomen are green. Abdominal segment V with a set of eye spots, one on each side of the midline. The meso- and metafemora, tibiae, and the exterior lobe of the protibiae are uniformly brown. The protibia interior lobe and the profemoral interior lobe are muddled brown with patches of darker and lighter areas, and the profemoral exterior lobe is uniformly green.

***Morphology*. *Head*.** Head capsule ca ¼ longer than wide, with a vertex that is smooth, lacking distinct granulation; the posteromedial tubercle is not prominent, only slightly raised above the head capsule (Fig. 24B). Frontal convexities stout, not strongly projecting, ending in a blunt, rounded apex. Compound eyes are large and bulbous, occupying slightly < ½ of the head capsule lateral margins (Fig. 24B). Between and slightly posterior to the compound eyes are three protruding, well-formed ocelli (Fig. 24B). Antennal fields are slightly wider than the scapus width. ***Antennae*.** Antennae (including the scapus and pedicellus) consist of 24 segments. The scapus and pedicellus are smooth, lacking setae, the following 18 segments are covered evenly in thin, dark setae which in most cases are approximately as long as the antennae segment is wide. The terminal four antennal segments have short, dense setae, notably different from the long sparse setae on the other segments. Each antennomere on the distal end projects ventrally slightly, so the overall antenna has a serrate appearance. ***Thorax*.** Pronotum trapezoidal in shape, with the anterior width similar to the lateral lengths, and the posterior width ca ½ the anterior width. Therefore, the lateral margins distinctly converge to the narrow posterior (Fig. 24B). Pronotum anterior margin slightly concave; lateral margins relatively straight to slightly converging. Anterior and lateral margins of the pronotum with distinctly defined rims (Fig. 24B). Face of the pronotum is marked by a relatively smooth surface without heavy granulation or wrinkling. The only notable feature on the face of the pronotum is a slight sagittal furrow, a notable central pit, a moderately formed perpendicular furrow just anterior to the central pit, and two distinct pits along the anterior margin behind the anterior rim (Fig. 24B). Prosternum and mesosternum relatively smooth, lacking granulation. Metasternum slightly wrinkled on the anterior and lateral margins, and heavily wrinkled on the posterior. Prescutum anterior wider than the prescutum is long, with lateral margins slightly converging to the posterior which is ca ⅙ narrower than the anterior rim width (Fig. 24B). Prescutum lateral rims with slight granulation throughout their length (Fig. 24B). The surface of the prescutum is mostly smooth with only moderate, sparse granulation. The prescutum surface is raised along the sagittal crest, which is slightly textured (Fig. 24B). Prescutum anterior rim moderately formed but lacking a prominent sagittal spine (Fig. 24B). Mesopleura narrow, diverging slightly on the anterior ⅗, and then widening slightly on the posterior ⅖ (Fig. 24B). Mesopleuron lateral margin with five or six tubercles situated on the anterior ⅗ (with the tubercles increasing in size from the anterior to the posterior) and the posterior ⅖ of the mesopleuron is smooth, lacking tubercles (Fig. 24B). Face of the mesopleuron slightly wrinkled and marked with one distinct pit near the middle. ***Wings*.** Tegmina short, extending partially onto abdominal segment II. Tegmen wing venation: the subcosta (Sc) is the first vein, runs relatively straight for ca ½ of the wing length and terminates on the margin. The radius (R) spans the entire length of the tegmen, running as the radial sector (Rs) straight through the center of the tegmen to the apex after the first radius (R1) branches near the center of the wing and runs to the posterior ⅓ margin where it terminates. The media (M) spans the entire length of the tegmen, running parallel with the radius (R) and radial sector (Rs) and terminates at the wing apex as the media anterior (MA) after the branching of a weakly formed media posterior (MP) near the middle of the tegmen which terminates slightly posterior to the tegmen midline. The cubitus (Cu) runs through the tegmen surface angled away from the media (M) for ca ⅓ of the length to the tegmen margin and then runs along the margin where it fades before meeting the apex. The first anal (1A) vein runs subparallel to the cubitus until they meet ca ¼ of the way through the tegmen length. Alae well-developed in an oval fan configuration, reaching onto abdominal segment VIII or IX. Ala wing venation: the costa (C) is present along the entire foremargin giving stability to the wing. The subcosta (Sc) is fused with the radius for the anterior ⅗ of the ala length, then splits and runs parallel with the wing margin until it fades before reaching the apex. The radius (R) branches ca ⅔ of the way through the ala length into the first radius (R1) and radial sector (Rs) which run gradually diverging through the first ½ of their lengths, then run parallel until fusing with the cubitus at different locations on the posterior ⅕ of the wing. The media (M) branches early, near the anterior ⅛ into the media anterior (MA) and the media posterior (MP) which run diverging for ⅓ of their lengths, then parallel for ⅓, and then converge for the final ⅓. The media anterior fuses with the cubitus (Cu) near the posterior ¼ of the ala length. The media posterior fades before fully fusing with the cubitus. The cubitus runs unbranched and terminates at the wing apex after the media anterior, first radius, and radial sector fuse with it at different locations. Of the anterior anal veins, the first anterior anal (1AA) fuses with the cubitus early on and then the first anterior anal branches from the cubitus ¾ of the way through the wing length where it uniformly diverges from the cubitus until it terminates at the wing margin. The anterior anal veins 2–7 (2AA–7AA) have a common origin and run unbranched in a folding fan pattern of relatively uniform spacing to the wing margin. The posterior anal veins (1PA–6PA) share a common origin separate from the anterior anal veins and run unbranched to the wing margin with slightly thinner spacing than the anterior anal veins. ***Abdomen*.** The abdomen’s general shape is boxy and broad with a maximum width of approximately ⅗ the abdomen length. Abdominal segment II diverges slightly, III diverges more prominently, IV is slightly angled with the anterior ½ diverging strongly and the posterior ½ running parallel, V through VII have margins which are nearly parallel, VIII converges slightly then arcs into a lobe on each side of the abdomen as it curls towards the genitalia, IX converges strongly inward towards the genitalia, and X projects away from IX into a pointed apex. ***Genitalia*.** Poculum rectangular in shape, broad with a width only slightly less than the width of abdominal segment X. The poculum ends in a margin which is relatively straight that passes just beyond the anterior margin of segment X (Fig. 24C). Cerci ovular, not particularly long, with ca 1⁄2 of their length extending from under abdominal segment X (Fig. 24C). The cerci are relatively flat, not strongly cupped, and have distinctly granular surfaces and setae throughout the surfaces. The cerci margins are marked with distinct, stiff setae. The vomer is triangular and stout with all margins approximately equal in length, with lateral margins relatively straight and converging evenly to the apex, which is armed with a singular stout hook curving into the paraproct. ***Legs*.** Profemoral exterior lobe broad and right angled, ca 1½× widener than the interior lobe. Profemoral exterior lobe proximal margin distinctly serrate, with four prominent teeth, with the proximal two the closest together and the other two variable on their location, either spaced evenly or both situated closer to the distal end of that margin. Profemoral exterior lobe distal margin typically smooth, lacking prominent serration, at most with slight granulation throughout or weakly formed teeth on the proximal end. Profemoral interior lobe begins approximately ⅓ of the way through the profemoral length, and arcs in a slight undulation with a maximum width of ca 3–4× the profemoral shaft width. Profemoral interior lobe is marked by three teeth on the distal end of the lobe, the proximal most is small, followed by two broadly triangular teeth. Mesofemoral exterior lobe is roundly triangular, relatively evenly weighted from end to end, and broad, with the proximal margin straight and smooth, and the distal margin slightly arcing and marked by three to five distinct serrate teeth. The greatest width of the mesofemoral exterior lobe is ca 2× the width of the mesofemoral shaft. The mesofemoral interior lobe is thinner than the exterior lobe and is unevenly weighted from end to end with the widest portion near the distal ⅓. The proximal ⅓ of the mesofemoral interior lobe is narrow with a rounded arc on the distal ⅔. There is unevenly spaced and variable sized serration throughout the full margin of the mesofemoral interior lobe (typically seven or eight serrate teeth). Metafemoral exterior lobe arcs thinly along the metafemoral shaft, with a maximum width slightly thinner than the metafemoral shaft width. Metafemoral exterior lobe lacks dentition throughout most of its length, with only the distal ⅓ marked by three or four small serrate teeth. Metafemoral interior lobe is narrow and straight for the proximal ½ and then on the distal ½ there is a small, rounded lobe that is approximately as wide as the shaft width. There is serration of variable size and spacing throughout the length (six to eight teeth), but the distal ½ is marked by more prominent serration. Protibial exterior lobe is a thin scalene triangle weighted towards the distal end which is ca 3× as wide as the protibial shaft width. The protibial exterior lobe distal margin is marked with several small nodes and the remainder of the lobe is smooth. Protibial interior lobe is also a scalene triangle, but it is slightly wider than the exterior lobe, with the widest portion the distal ⅖, and the full length is smooth, lacking serration. Mesotibial exterior lobe is roughly a narrow right triangle which is weighted towards the distal ⅓ and has a greatest width ca 3× the width of the mesotibial shaft. The broadest point of the mesotibial exterior lobe has slight granulation/small serration. Metatibial exterior lobe is roughly a broad right triangle which is weighted towards the distal ¼ and has a greatest width ca 3× the width of the metatibial shaft. Mesotibiae and metatibiae lack interior lobes.

***Measurements of holotype male Coll RC 16-007* [mm].** Length of body (including cerci and head, excluding antennae) 66.0, length/width of head 4.1/3.7, antennae 27.3, pronotum 2.9, mesonotum 3.6, length/width of tegmina 13.8/5.1, length of alae 52.3, greatest width of abdomen 25.0, profemora 14.0, mesofemora 9.7, metafemora 10.2, protibiae 8.1, mesotibiae 7.0, metatibiae 8.6.

***Measurements of paratype males, given as a range for smallest to largest* [mm].** Length of body (including cerci and head, excluding antennae) 59.4–71.4, length/width of head 4.0–4.5/3.3–4.0, antennae 25.4–27.6, pronotum 3.0–3.7, mesonotum 3.3–5.1, length/width of tegmina 11.9–15.7/4.8–6.0, length of alae 45.0–57.2, greatest width of abdomen 24.2–26.7, profemora 11.4–14.6, mesofemora 9.0–9.3, metafemora 9.0–9.5, protibiae 6.9–8.0, mesotibiae 6.7–7.8, metatibiae 6.6–8.6.

##### Etymology.

Proper noun, Greek in origin. Following a mythological trend started by Gray (1843) who named species based upon characters from the tenth labor of Heracles. Interestingly, despite several of Gray’s names following this myth, he never named a species following the main character, Heracles himself. We herein would like to close Gray’s phylliid focused chapter on the tenth labor of Heracles by bestowing the name upon a large and impressive species, worthy of such a mighty character.

##### Distribution.

Records for this species are primarily from southern and central Vietnam (from the provinces of Kon Tum, Da Nang, Gia Lai, Lam Dong, Quang Nam, and Thua Thien Hue) and additionally one record from the northern Vietnam province of Nghe An is known (Fig. 15B).

##### Remarks.

Specimens of this Vietnamese population were originally thought to represent range expansions for *Pulchriphylliummaethoraniae*. Therefore, this population was tentatively identified within Bank et al. (2021) as Pulchriphylliumcf.maethoraniae due to a lack of true *Pulchriphylliummaethoraniae* samples from Thailand at the time. Yet, additional sampling from throughout mainland Asia (including in Thailand near the *Pulchriphylliummaethoraniae* type locality; Fig. 15B) has revealed that this complex of similar-looking specimens are instead three well-defined species: *Pulchriphylliummaethoraniae*, *Pulchriphylliumsinense*, and the Vietnamese species herein described as *Pulchriphylliumheracles* sp. nov. (Fig. 24).

Interestingly, despite near identical morphology with *Pulchriphylliummaethoraniae* and *Pulchriphylliumsinense*, *Pulchriphylliumheracles* sp. nov. has been recovered in the molecular phylogeny as entirely unrelated to *Pulchriphylliummaethoraniae* and *Pulchriphylliumsinense* (which were recovered as sister to each other; Fig. 2). Hopefully future collection efforts in Vietnam will reveal the female, egg, and freshly hatched nymph morphology to allow better morphological comparison of *Pulchriphylliumheracles* sp. nov. with congeners.

#### ﻿ Pulchriphyllium bhaskarai

Taxon classificationAnimaliaPhasmatodeaPhylliidae

sp. nov.

ADDADC45-3A9D-5086-B753-6702F3E4C807

https://zoobank.org/4C6707C0-3155-46D9-8ED2-620765AF8E71

Figs 8H , 21E , 25 , 26 , 27 , 28 , 29 , 30

##### Material examined.

**Figure 25.**
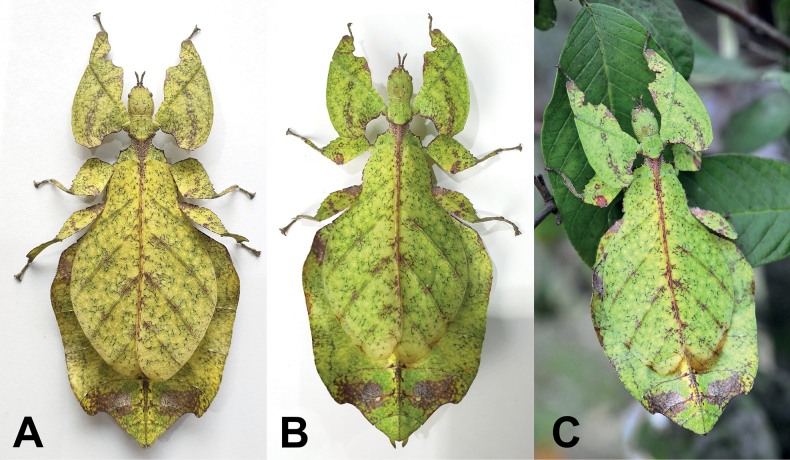
*Pulchriphylliumbhaskarai* sp. nov. live holotype female. Photographs by Edy Bhaskara (Indonesia) **A** coloration shortly after being found in the wild, dorsal habitus **B** altered coloration after several months in captivity, dorsal habitus **C** habitus, dorsolateral view.

***Holotype*** ♀: “Indonesia, West Java, Sukabumi, Kabandungan Village, Mt. Halimun, found 19^th^ August 2020, first started laying eggs 16^th^ September 2020, died 8^th^ December 2020; laid a total of 78 eggs” (Fig. 25). Deposited in the Montreal Insectarium, Quebec, Canada (IMQC). ***Paratypes***: (19♂♂, 36♀♀, 2 eggs) See Suppl. material 1 for details about paratype specimens, their collection data, and depositories.

##### Differentiation.

Adult males and females have proven difficult to differentiate morphologically from *Pulchriphylliumgiganteum*. Within both sexes of both species there is significant overlap in overall size and due to intraspecific variation, few to no features have allowed confident differentiation. Instead, the only reliable and easily observed morphological differences are the freshly hatched nymphs and egg morphology.

**Figure 26.**
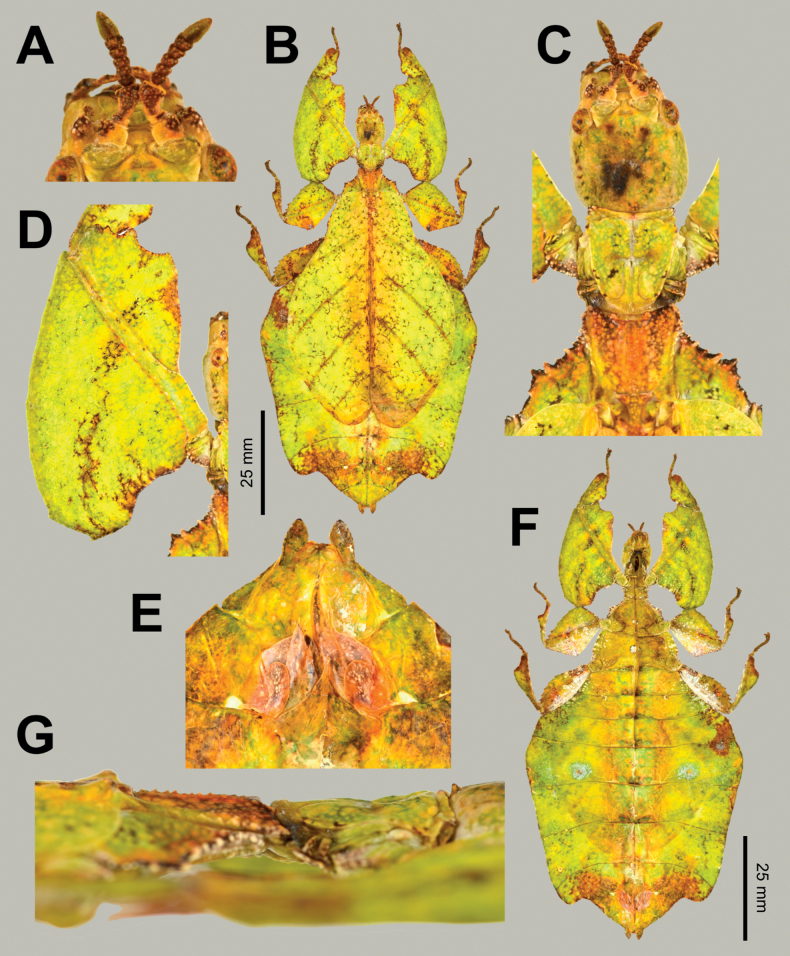
*Pulchriphylliumbhaskarai* sp. nov. female holotype **A** details of the antennae, dorsal **B** dorsal habitus **C** details of the head and thorax, dorsal **D** details of the profemora **E** genitalia details, ventral **F** ventral habitus **G** lateral view of the thorax (anterior to the right). Scale bars: 25 mm (**B, F)**.

**Females** are very similar to *Pulchriphylliumgiganteum* but can be differentiated by the mesopleurae. In *Pulchriphylliumgiganteum* the posterior two or three spines are grouped together as a broad set projecting away from the mesopleuron margin, while in *Pulchriphylliumbhaskarai* sp. nov. these posterior-most spines are less prominent, only slightly projecting away from the mesopleuron margin (Fig. 26C).

**Figure 27.**
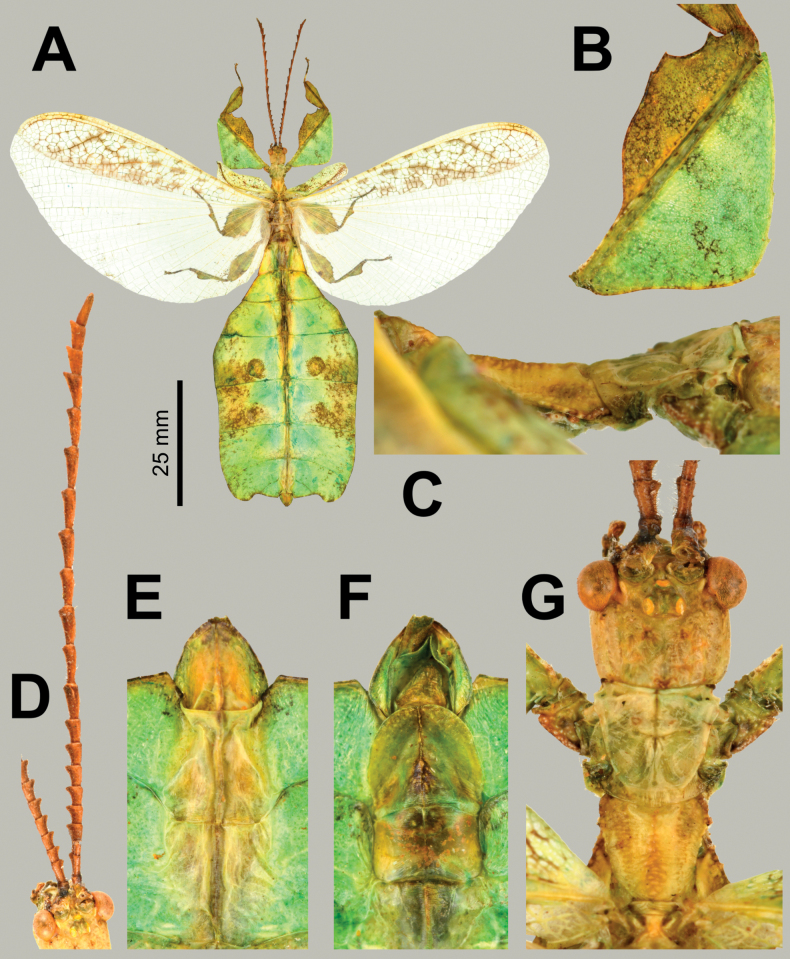
*Pulchriphylliumbhaskarai* sp. nov. male paratype, Coll. RC #21-032 **A** dorsal habitus **B** details of the profemora **C** lateral view of the thorax (anterior to the right) **D** details of the antennae, dorsolateral view **E** terminalia details, dorsal **F** genitalia details, ventral **G** details of the head and thorax, dorsal. Scale bar: 25 mm (**A)**.

**Males** (Fig. 27) have proven impossible to differentiate from *Pulchriphylliumgiganteum* with any certainty from morphology alone. Upon review of material from multiple locations and from both fresh and antique specimens, no single feature was consistently different enough to be useful (Cumming et al. 2020b).

**Freshly hatched nymphs** (Figs 8H, 29) can easily be differentiated from congeners as the abdomen is notably thinner than any other *Pulchriphyllium* species, with a maximum width only ca ⅗ of the abdomen length (where all other *Pulchriphyllium* species have a wider and longer abdominal shape, with a width nearly equal to the abdomen length).

**Figure 28.**
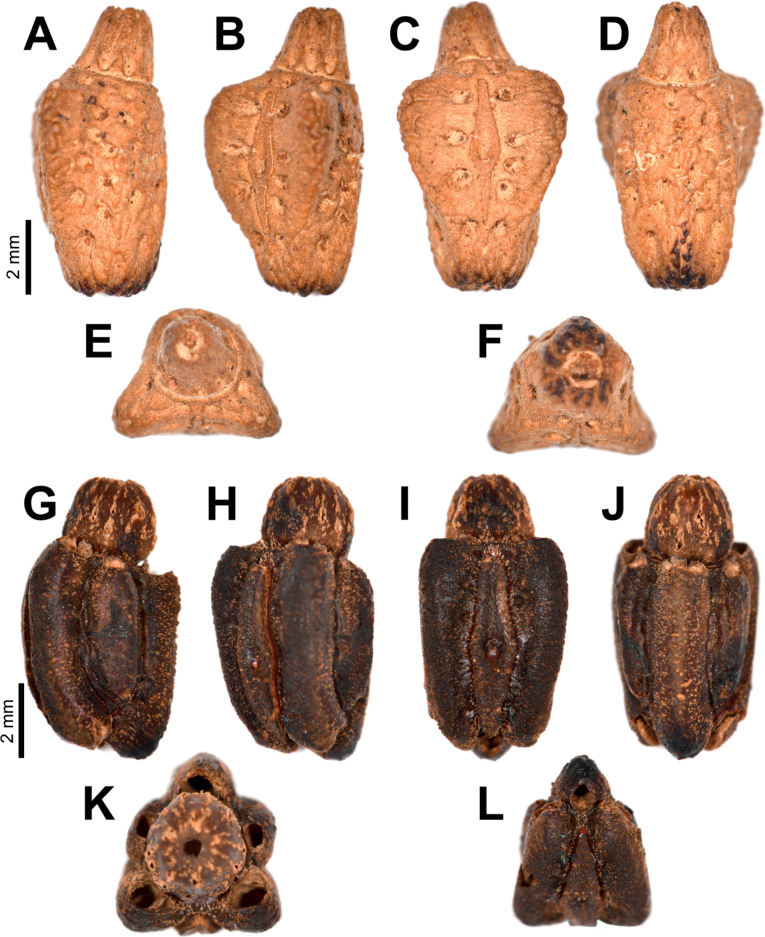
**A–F***Pulchriphylliumgiganteum* egg capsule, IMQC collection **A** lateral view **B** dorso-lateral view **C** dorsal view **D** opercular (anterior) view **E** posterior view **F** ventral view **G–L***Pulchriphylliumbhaskarai* sp. nov. egg capsule, IMQC collection (Coll RC 21-034) **G** lateral view **H** dorso-lateral view **I** dorsal view **J** opercular (anterior) view **K** posterior view **L** ventral view. Photographs by René Limoges (IMQC). Scale bars: 2 mm (**A–L**).

The eggs of *Pulchriphylliumbhaskarai* sp. nov. are rather unique and can easily differentiate this species from *Pulchriphylliumgiganteum* by the presence of five laterally running semi-hollow tubes which are open on the anterior end (two tubes are located on each of the lateral surfaces and one tube is located on the ventral surface: Fig. 28K). Additionally, the operculum is notably wider than long in *Pulchriphylliumbhaskarai* sp. nov. (Fig. 28H) but approximately as long as wide and slightly tapered in *Pulchriphylliumgiganteum* (Fig. 28B).

**Figure 29.**
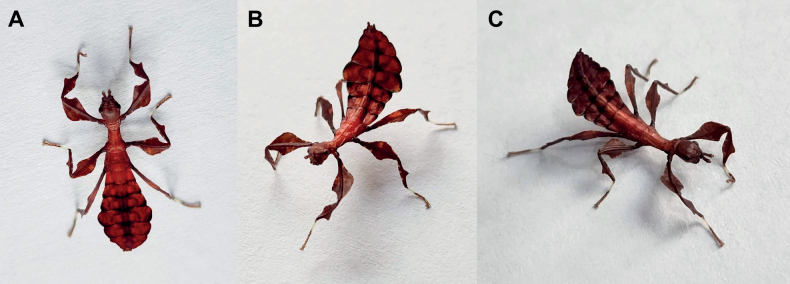
*Pulchriphylliumbhaskarai* sp. nov. live freshly hatched nymphs **A** dorsal habitus **B** habitus, dorsolateral view **C** habitus, dorsolateral view. Photographs by Edy Bhaskara (Indonesia).

##### Description.

**Female. *Coloration*.** The coloration description is based upon the holotype living individual when it was alive (Fig. 25). Interestingly the coloration altered throughout its development after it was brought into captivity. When first collected from the wild the overall base coloration was primarily yellow with muddled, variable patches of lime green to brown/gray coloration (Fig. 25A). After several months in captivity the individual shifted the yellow base coloration to lime green, with variable patches of yellow and brown/gray coloration (Fig. 25B, C). The areas that were brown/gray altered little during this period, only the base coloration appears to have changed.

***Morphology*. *Head*.** Head capsule is slightly longer than wide, with a vertex that is marked throughout by moderately formed granulation and a singularly pointed posteromedial tubercle which is notably larger than the other granulation on the head capsule (Fig. 26C). Frontal convexity triangular with a blunt point, which has a slightly granular surface and three to five setae near the apex. The compound eyes slightly protrude from the head capsule, not overly large, with a width taking up ca ¼ of the head capsule lateral margins (Fig. 26C). Ocelli appear to be either very weakly developed, or absent. Antennal fields slightly wider than the width of the first antennomere. ***Antennae*.** Antennae consist of nine segments including the scapus and pedicellus, with the terminal segment approximately the same length as the preceding three segments’ lengths combined (Fig. 26A). Antennomeres I–VIII sparsely marked with few transparent setae. Terminal antennomere covered densely in short, brown setae (Fig. 26A). ***Thorax*.** Pronotum with slight concave anterior margin and lateral margins that are straight and subparallel for the anterior ⅔ and the posterior ⅓ converges slightly to the posterior margin that is ⅔ the width of the anterior margin (Fig. 26C). The pronotum surface is marked with prominent granulation throughout, a distinct sagittal furrow, and a smaller perpendicular furrow near the center (Fig. 26C). The pronotum has distinctly formed anterior and lateral rims and a weakly formed posterior rim (Fig. 26C). Prosternum, mesosternum, and metanotum are marked by nodes of various size and spacing, with those along the margins more prominent. Prescutum approximately as long as the greatest width, with lateral rims marked by a granular surface with those on the anterior third larger than the others (Fig. 26C). Prescutum anterior rim not strongly protruding, rim surface is granular and lacks a sagittal spine (Fig. 26C). Prescutum surface slightly raised along the sagittal plane and the entire surface is marked throughout with granulation. Mesopleura are narrow for the anterior ¼, then diverge strongly for the middle ½, bend prominently and then run only slightly diverging for the remainder of their lengths. On the mesopleuron near the distinct bend from prominently diverging to slightly diverging, there is a cluster of two or three tubercles which are grouped together and project slightly away from the mesopleuron margin (Fig. 26C). Mesopleuron lateral margin with eight or nine various sized tubercles, with those on the anterior typically smaller (Fig. 26C). Mesopleuron surface granular and marked with a distinct divot near the middle (Fig. 26C). ***Wings*.** Tegmina long, reaching at least ½ onto abdominal segment VII or slightly onto VIII. Tegmen venation; the subcosta (Sc) is the first vein in the forewing, runs parallel with the margin for the first ½, and then bends slightly and runs towards the margin where it terminates ca ⅕ of the way through the wing length. The radius (R) spans the central portion of the tegmen with two slightly diverging veins; the first radius (R1) branches ca ⅕ of the way through the tegmen length and terminates approximately ⅓ of the way through; the radial sector (Rs) branches ca ⅓ of the way through the tegmen length and terminates near the distal ⅖. The media (M) is bifurcate with the media anterior (MA) branching near the middle of the tegmen length and terminating near the distal ¼ and the media posterior (MP) branches near the distal ¼ of the tegmen and terminates near the apex. The cubitus (Cu) is also bifurcate, branching near the posterior ⅕ of the wing into the cubitus anterior (CuA) and cubitus posterior (CuP) which both terminate at or near the wing apex. The first anal vein (1A) is simple and fuses with the cubitus ca ⅕ of the way through the tegmen length. Alae rudimentary. ***Abdomen*.** Abdominal segments II through the anterior ½ of IV gradually diverging. The posterior ½ of segment IV through the anterior ½ of segment V weakly diverging. The posterior ½ of abdominal segment V through VII with straight, slightly converging margins. Abdominal segment VIII arcing inward to form a small lobe, followed by segment IX and X converging to the apex. ***Genitalia*.** Subgenital plate starts at the anterior margin of tergum VIII, is not particularly broad, only extending at most to the anterior margin of tergum X and terminating in a fine point (Fig. 26E). Gonapophyses VIII are long and moderately broad, nearly reaching the apex of abdominal tergum X; gonapophyses IX are shorter and broader (Fig. 26E). Cerci flat, with a relatively smooth surface and few detectable setae (Fig. 26E). ***Legs*.** Profemoral exterior lobe broad, slightly recurved with an acute angle, with a width ca 2× the width of the interior lobe (Fig. 26D). Edge of the profemoral exterior lobe primarily smooth or slightly lumpy with at most three dulled, small teeth on the recurved angle (Fig. 26D). Profemoral interior lobe only located on the distal ⅔ as an arcing lobe ca 2½× as wide as the greatest width of the profemoral shaft, with the distal ½ with four or five dulled triangular teeth of irregular size and spacing with looping gaps between them (Fig. 26D). The mesofemoral exterior lobe arcs from end to end in a rounded triangular shape that is weighted towards the distal ⅖ and is at most slightly wider than the mesofemoral shaft width. The proximal margin is smooth but the distal margin following the bend is marked with three or four serrate teeth. The mesofemoral interior lobe is ca 1½× as wide as the mesofemoral shaft, and also shaped as a rounded triangle, but the interior lobe is slightly weighted towards the proximal ⅓. The mesofemoral interior lobe proximal margin is smooth and the distal margin following the bend is marked by six or seven dulled, serrate teeth. The metafemoral exterior lobe arcs end to end hugging the metafemoral shaft and lacks teeth. The metafemoral interior lobe arcs gently from end to end but is slightly thicker on the distal ⅔ (which is at most as wide as the metafemoral shaft width). The distal ½ of the metafemoral interior lobe has five or six distinct serrate teeth. The protibial exterior lobe is a thin triangle weighted to the distal end with a maximum width of ca 1½× the width of the protibial shaft. The protibial interior lobe spans the entire length as a rounded triangle weighted slightly towards the distal ½ with a maximum width ca 2× the width of the protibiae shaft itself. Mesotibiae and metatibiae lacking interior lobes.

***Measurements of holotype female* [mm].** Length of body (including cerci and head, excluding antennae) 97.0, length/width of head 8.3/7.1, antennae 5.2, pronotum 6.2, mesonotum 4.9, length of tegmina 55.5, greatest width of abdomen 50.7, profemora 21.3, mesofemora 15.3, metafemora 18.9, protibiae 10.6, mesotibiae 11.1, metatibiae 15.6.

***Measurements of paratype females* [mm].** Length of body (including cerci and head, excluding antennae) 95.6–114.5, length/width of head 8.2–10.8/7.0–8.3, antennae 5.1–6.2, pronotum 6.1–6.8, mesonotum 4.8–5.5, length of tegmina 55.3–67.0, greatest width of abdomen 50.5–56.1, profemora 21.0–27.6, mesofemora 15.2–17.5, metafemora 18.7–21.3, protibiae 10.5–12.6, mesotibiae 11.0–13.0, metatibiae 15.4–17.8.

**Male. *Coloration*.** Coloration description based upon images of live specimens. Overall coloration is pale green throughout with somewhat variable patches of tan, brown, or black (Fig. 27A). The antennae, head, thorax, protibiae, profemoral interior lobe, mesotibiae, mesofemora, and metatibiae are brown. The sclerotized area of the alae has variable tan portions around the veins. Abdominal segments V and VI have variable patches of brown/tan, and segment V has a pair of large brown/black eye spots.

***Morphology*. *Head*.** Head capsule approximately as wide as long, with a vertex that is slightly wrinkled/rough textured. The posteromedial tubercle is small but distinctly raised above the head capsule. Frontal convexity stout and ending with a blunt point and marked with only a few short setae. Compound eyes bulbous, occupying slightly > ⅖ of the head capsule lateral margins (Fig. 27G). There are three well-developed ocelli located between and slightly posterior to the compound eyes. Antennal fields are approximately as wide as the scapus. ***Antennae*.** Antennae (including the scapus and pedicellus) consist of 22 segments (Fig. 27D). The scapus and pedicellus are bare, the following 17 segments are covered with thin, short setae, and the terminal three segments have notably shorter and denser setae. Each antennomere on the distal end projects ventrally slightly, so the overall antenna has a serrate appearance. ***Thorax*.** Pronotum with anterior margin gently concave, with a well-defined margin. Lateral margins have moderately formed rims that converge gently to a straight posterior margin that lacks a distinct margin and is approximately ½ the width of the anterior rim (Fig. 27G). Face of the pronotum is smooth or slightly wrinkled and marked with a distinct sagittal furrow, and a slight perpendicular furrow near the center (Fig. 27G). The prosternum surface is slightly granular, and the mesosternum and metasternum surfaces are smooth centrally, but have slightly granular margins. The prescutum is approximately as long as wide, with lateral margins slightly converging to the posterior margin which is ca ¾ as wide as the anterior margin. Prescutum lateral rims roughly textured, with the nodes on the anterior more prominent than the posterior. The surface of the prescutum is heavily granulose and moderately wrinkled, with the surface slightly raised along the sagittal plane. Prescutum anterior rim weakly formed, surface only slightly granular, and lacking a distinct central tubercle (Fig. 27G). Mesopleura narrow for the anterior ⅓, then gradually diverging steadily throughout their lengths (Fig. 27G). Mesopleuron lateral margin with four or five small tubercles throughout the length, with the posterior two slightly more prominent (Fig. 27G). Face of the mesopleuron with a slightly wrinkled texture. ***Wings*.** Tegmina short, extending ¾ of the way onto abdominal segment II. Tegmen wing venation: the subcosta (Sc) is the first vein, runs relatively straight for ca ½ of the wing length and terminates on the margin. The radius (R) spans the entire length of the tegmen, running as the radial sector (Rs) straight through the center of the tegmen to the apex after the first radius (R1) branches near the distal ⅓ of the wing and terminates near the posterior margin. The media (M) spans the entire length of the tegmen, running parallel with the radius (R) and radial sector (Rs) and terminates at the wing apex as the media anterior (MA) after the branching of a weakly formed media posterior (MP) near the proximal ⅓ of the tegmen. The cubitus (Cu) runs through the tegmen surface angled away from the media (M) for slightly < ½ of the tegmen length to the margin and then runs along the margin where it fades before reaching the apex. The first anal (1A) vein runs subparallel to the cubitus until they meet ca ⅓ of the way through the tegmen length. Alae well-developed in an oval fan configuration, reaching onto abdominal segment VIII or IX. Ala wing venation: the costa (C) is present along the entire foremargin giving stability to the wing. The subcosta (Sc) is fused with the radius for the anterior ⅗ of the ala length, then splits and runs parallel with the ala margin until it fades before reaching the apex. The radius (R) branches ca ⅔ of the way through the ala length into the first radius (R1) and radial sector (Rs) which run gradually diverging until they fuse with the cubitus at different locations. The media (M) branches early, near the anterior ⅛ into the media anterior (MA) and the media posterior (MP) which run diverging for ⅓ of their lengths, then parallel for ⅓ of their lengths, and converging for the final ⅓. Both the media anterior and the media posterior weakly fuse with the cubitus at different locations. The cubitus runs unbranched and terminates at the wing apex after the media anterior, media posterior, first radius, and radial sector fuse with it at different locations. Of the anterior anal veins, the first anterior anal (1AA) fuses with the cubitus ca ⅕ of the way through the wing length and then the first anterior anal branches from the cubitus on the distal ⅓ where it uniformly diverges from the cubitus until it terminates at the wing margin. The anterior anal veins two–seven (2AA–7AA) have a common origin and run unbranched in a folding fan pattern of relatively uniform spacing to the wing margin. The posterior anal veins (1PA–6PA) share a common origin separate from the anterior anal veins and run unbranched to the wing margin with slightly thinner spacing than the anterior anal veins. ***Abdomen*.** Abdominal segments II through the anterior ½ of segment IV gradually diverge. The posterior ½ of segment IV through the anterior ½ of segment V diverge slightly less than the previous segments. The posterior ½ of segment V through segment VII have parallel margins, giving the abdomen a rectangular appearance. The posterior end of abdominal segment VIII converges significantly and forms a slight posterior pointing lobe. Abdominal segment IX has margins converging directly to abdominal segment X which is small and converges to a blunt apex. ***Genitalia*.** Poculum broad and rounded (with a maximum width broader than abdominal segment X) and ending in a blunt apex that passes beyond the anterior margin of segment X (Fig. 27F). Cerci slender, with ca ½ of their length extending out from under abdominal segment X. The cerci are slightly cupped, have a granulose surface, and numerous long setae along the margins. Vomer broad with straight margins evenly converging to the apex, which is armed with a singular, broad upwards turning hook. ***Legs*.** Profemoral exterior lobe broad, nearly right angled, ca 1½× wider than the interior lobe, with the proximal margin smooth and the distal margin marked with three to five teeth near the bend (Fig. 27B). Profemoral interior lobe only on the distal ⅔ of the profemoral shaft, roundly arcing, with the distal ½ ornamented with four teeth arranged in a two-two pattern, with a large looping gap between the sets (Fig. 27B). Mesofemoral exterior lobe is roundly triangular, slightly weighted towards the distal end, and broad (ca 1½× as wide as the mesofemoral shaft width), with the proximal margin straight and smooth, and the distal margin slightly arcing and marked by four distinct serrate teeth. The mesofemoral interior lobe is slightly thinner than the exterior lobe and it is relatively evenly weighted from end to end. The proximal ½ of the mesofemoral interior lobe is straight and smooth, while the distal ½ is armed with six or seven serrate teeth. Metafemoral exterior lobe arcs thinly along the metafemoral shaft, with a maximum width slightly thinner than the metafemoral shaft width. Metafemoral exterior lobe lacks dentition throughout most of its length, with only the distal ⅓ marked by three or four small serrate teeth. Metafemoral interior lobe is narrow and straight for the proximal ½ and then on the distal ½ it widens out slightly to a maximum width approximately as wide as the metafemoral shaft width. The distal ½ of the metafemoral interior lobe is armed with six or seven serrate teeth. The protibial exterior lobe is only present on the distal ½ as a thin arcing lobe at most 2× the width of the protibial shaft. Protibial interior lobe is a rounded, thin scalene triangle slightly weighted just distal to the midlength, with a maximum width ca 3× the width of the protibial shaft. Mesotibial exterior lobe is a small, triangular spur, only slightly wider than the width of the mesotibial shaft, situated just distal to the midlength, and occupying slightly < ⅓ of the overall length. The metatibial exterior lobe is ca 2× the size of the mesotibial exterior lobe but slightly more rounded in shape, is situated only on the distal ⅔ of the length and has a maximum width of 1½× the metatibial shaft width. Mesotibiae and metatibiae lack interior lobes.

***Measurements of paratype males* [mm].** Length of body (including cerci and head, excluding antennae) 73.4–74.0, length/width of head 4.5–4.6/2.1–2.2, antennae 25.7–25.9, pronotum 3.2–3.3, mesonotum 2.9–3.0, length of tegmina 14.3–14.5, length of alae 56.8–57.0, greatest width of abdomen 29.0–29.2, profemora 14.8–14.9, mesofemora 10.5–10.6, metafemora 11.2–11.3, protibiae 7.4–7.6, mesotibiae 6.9–7.0, metatibiae 9.0–9.2.

**Eggs.** (Fig. 28G–L). The overall color is dark brown, all surfaces are roughly textured, with some of the rough textured areas lighter brown in color. The cross-section shape is approximately pentagonal, with the dorsal surface the widest surface. Each lateral surface has two longitudinally running semi hollow tubes running the full length of the capsule with their openings on the anterior end (Fig. 28K). When viewed laterally, the dorsal surface is slightly arcing, and the ventral surface is straight (Fig. 28G). The dorsal surface has the micropylar plate which spans the entire length of the capsule in a roughly, rounded, diamond shape with the micropylar cup centrally located (Fig. 28I). The area around the micropylar plate lacks distinct features, and is relatively simple, only roughly textured (Fig. 28I). The operculum is slightly ovular, wider than long, and is semi hollow, ending in a distinct opening (Fig. 28K). The ventral surface has a fully hollow tube running the full length, with openings on each end (Fig. 28K–L). On the posterior (Fig. 28L) only the ventral tube has an opening, the rest of the surface is rounded from the closed bases of the lateral, longitudinally running tubes.

***Measurements* [mm].** Length (including operculum): 7.9–8.0; maximum width of capsule when viewed from lateral aspect 4.2–4.3; length of micropylar plate 5.0–5.1.

##### Newly hatched nymphs.

(Fig. 29). The general color throughout the body is vermillion with muddled lighter and darker areas. The basitarsi are white, with the remaining tarsal segments a similar red to the body base color. All tibiae have well-developed exterior lobes of a similar shape, a narrow triangle occupying the central ⅓ of the tibiae. The protibial interior lobe is prominent, gently arcing the full length of the tibia. The protibial lobe has a width that is ca 2× as wide as the protibia shaft width. The profemoral interior lobe occupies the distal ⅔, is broad and obtusely angled, with a width that is slightly larger than 2½× the profemoral shaft width. The profemoral interior lobe has two weakly formed teeth on each end of the distal margin. The profemoral exterior lobe is of a similar shape as the interior lobe, but differs in being slightly wider, lacking distinct serration, and the proximal end reaches closer to the base of the profemora. All femoral lobes have mild serration, typically only two or three dulled teeth on the distal halves of the lobes. The meso- and metafemoral interior lobes span end to end, but are more heavily weighted towards the distal end, with the proximal end thin. The meso- and metafemoral exterior lobes are slightly more evenly spread across their lengths and slightly thinner, giving them a more arcing appearance. The head and lobes of the legs are of a similar burnt red coloration while the thorax is brighter red. The abdomen base color is vermillion with darker red to black markings along the margins and looping along the central abdominal cavity, giving the appearance of spots. The abdomen is notably thinner than any other *Pulchriphyllium* species, with a maximum width ca ⅗ of the abdomen length. The widest point of the abdomen is abdominal segment V.

##### Etymology.

Patronym. Named to honor Edy Bhaskara who supplied the specimens which solved the mystery surrounding this species. Previously only males were known, which are morphologically indistinguishable from *Pulchriphylliumgiganteum*, until Edy Bhaskara supplied the authors with fresh specimens (which allowed DNA analyses) as well as adult females, freshly hatched nymphs, and eggs, all of which allowed a full understanding to be generated for this species and allowed differentiation from congenerics.

##### Distribution.

At present only known from Java, Indonesia, with records from throughout the island (Suppl. material 1).

##### Remarks.

Originally, it was thought that the “giganteum”-like population on Java, Indonesia was simply a range expansion for *Pulchriphylliumgiganteum* (Cumming et al. 2020b). This erroneous assumption was based solely upon male morphology as no significant morphological features could be identified at the time. It was not until fresh specimens could be sequenced and the adult female, freshly hatched nymph, and the egg morphology were known that it became apparent that this “giganteum”-like species from Java was unique and undescribed.

Within the DNA-based analyses of Bank et al. (2021), this population was included as “*Pulchriphyllium* sp. 2” and not recovered as close relative to *Pulchriphylliumgiganteum* from Malaysia, but instead was recovered with weak support to be sister to a clade comprising *Pulchriphylliumrimiae* (Seow-Choen, 2017), *Pulchriphylliumshurei* (Cumming & Le Tirant, 2018), and *Pulchriphylliummannani* (Seow-Choen, 2017). Herein, the Javan taxon was instead recovered with high support as sister to all other included *Pulchriphyllium* species (Fig. 2). Following DNA analyses, we were sent images of adult female specimens (Fig. 25) by Edy Bhaskara (Java, Indonesia) which, although appearing very similar to *Pulchriphylliumgiganteum* from Malaysia, the Javan taxon has less prominent mesopleurae (Fig. 26C). Additionally, Edy Bhaskara was able to share images of the eggs (Fig. 28G–L) and freshly hatched nymphs (Fig. 29), both of which added to our understanding of the uniqueness of this species. Upon review of all available evidence for this taxon, this population can now confidently be identified as an undescribed species and described.

Thanks to DNA analyses, the geographic distribution of *Pulchriphylliumgiganteum* has been clarified to encompass West Malaysia, West Borneo, and North Borneo as a single species. Unfortunately, Sumatra and Nias island leave an area of significant uncertainty as no DNA samples are yet available, and to date only male specimens have been observed/collected (which look identical to both *Pulchriphylliumgiganteum* and *Pulchriphylliumbhaskarai* sp. nov.). Interestingly, despite *Pulchriphylliumbhaskarai* sp. nov. being presently understood as endemic to Java, *Pulchriphylliumgiganteum* has a wide range from South Thailand, West Malaysia, and throughout Borneo (Cumming et al. 2020b, Jiaranaisakul and Pawangkhanant 2022). Hopefully, future collecting efforts on the islands of Sumatra and Nias will produce DNA data as well as the adult female, egg, and freshly hatched nymph morphology, and thus allow these populations to be identified.

The eggs of *Pulchriphylliumbhaskarai* sp. nov. are quite unique, as they lack the typical *Pulchriphyllium* dense fins, and instead have rigid, open tubes running along the egg surfaces (Fig. 28K). No other phylliid eggs have such structures, and these rigid tubes are only similar to “type 7” eggs characterized within Büscher et al. (2023) due to texture/material, but their overall shape is wholly unique. Within our recovered phylogeny (Fig. 2) *Pulchriphylliumbhaskarai* sp. nov. was recovered as sister to all other *Pulchriphyllium* species, a placement which is not surprising as within Büscher et al. (2023)*Pulchriphylliumgiganteum* with eggs that are also unlike most *Pulchriphyllium* species was recovered as external to the other included *Pulchriphyllium* species. Within our recovered phylogeny (Fig. 2) *Pulchriphylliumbhaskarai* sp. nov., *Pulchriphylliumgiganteum*, and a clade formed by three species whose eggs are presently unknown (*Pulchriphylliumrimiae*, *Pulchriphylliumshurei*, and *Pulchriphylliummannani*) were recovered as external to the bulk of the *Pulchriphyllium* species which have the dense fins. This leaves us speculating what egg morphology *Pulchriphylliumrimiae*, *Pulchriphylliumshurei*, and *Pulchriphylliummannani* may have as these species (which are only known from males at the moment) are morphologically unique and not nested within the greater *Pulchriphyllium*. Hopefully females of these species can be located one day, and their egg morphology identified in order to better frame the evolution of phylliid egg morphology and fill in this missing knowledge.

**Figure 30.**
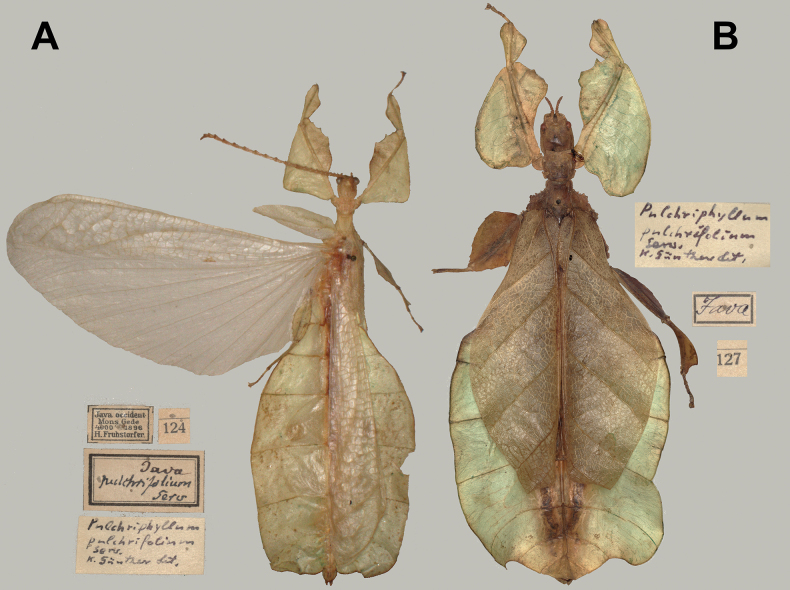
Paratype historic pair of *Pulchriphylliumbhaskarai* sp. nov. from MZPW**A** male with data labels inset to the lower left **B** female with data labels inset to the right.

#### ﻿ Pulchriphyllium anangu

Taxon classificationAnimaliaPhasmatodeaPhylliidae

sp. nov.

BE80A084-F2F6-5F38-A4F2-9B8BB377454D

https://zoobank.org/2F18CD6D-191F-47B9-9197-415956C367DD

**Figure 31.**
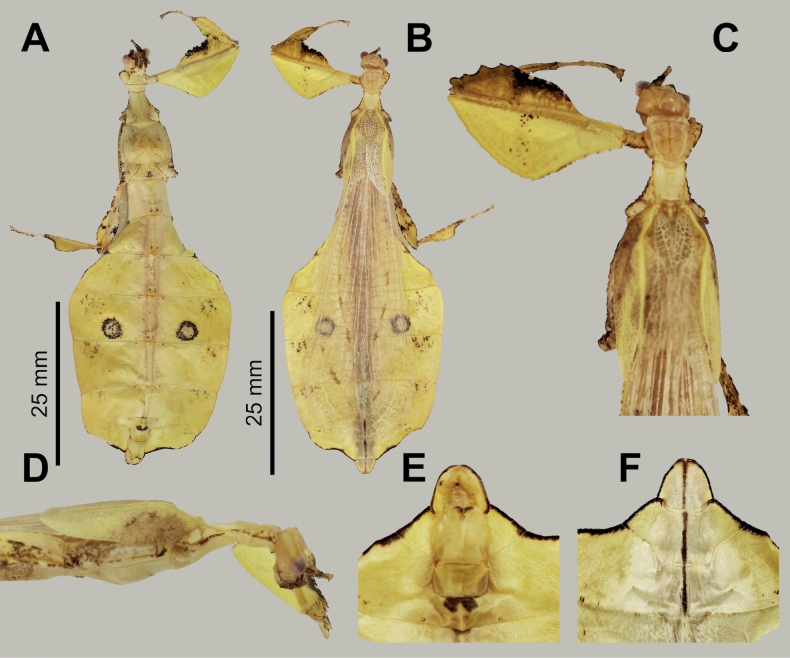
Male holotype *Pulchriphylliumanangu* sp. nov. (BYU) **A** ventral, habitus **B** dorsal, habitus **C** details of the front leg, head, and thorax, dorsal **D** details of the head through thorax, lateral **E** genitalia, ventral **F** terminalia, dorsal. Photographs taken by JBL.

**Figure 32.**
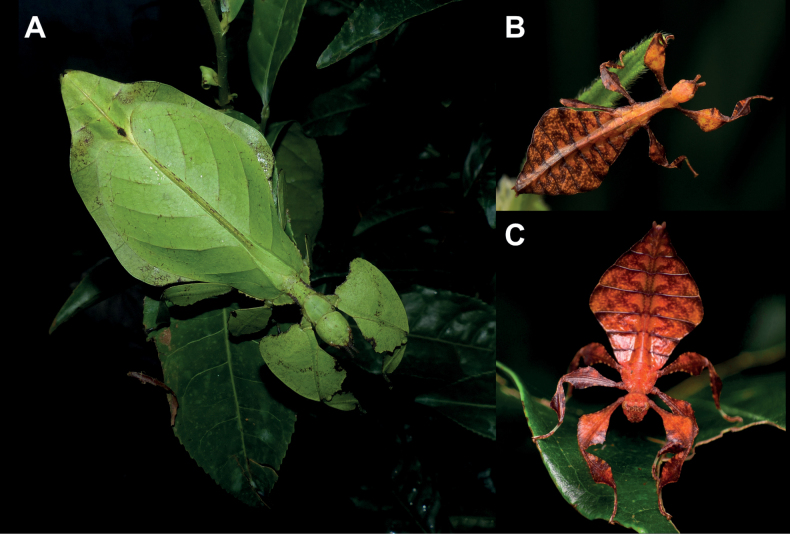
Live *Pulchriphylliumanangu* sp. nov. **A** adult female, dorsal habitus, observed July 2016 at Kadumane Estate, India by iNaturalist user @ashwinv (Ashwin Viswanathan) https://www.inaturalist.org/observations/29374627) **B** freshly hatched nymph, observed October 2021 at Chikkamagaluru, Karnataka, India by iNaturalist user @prashanthakrishnamc (Prashantha Krishna M C) https://www.inaturalist.org/observations/98984918) **C** freshly hatched nymph, observed January 2021 in Ponmudi, India by iNaturalist user @hari_krishnan_ (Harikrishnan S) https://www.inaturalist.org/observations/70254070). Uploaded to iNaturalist and used under license CC BY-NC 4.0.

**Figure 33.**
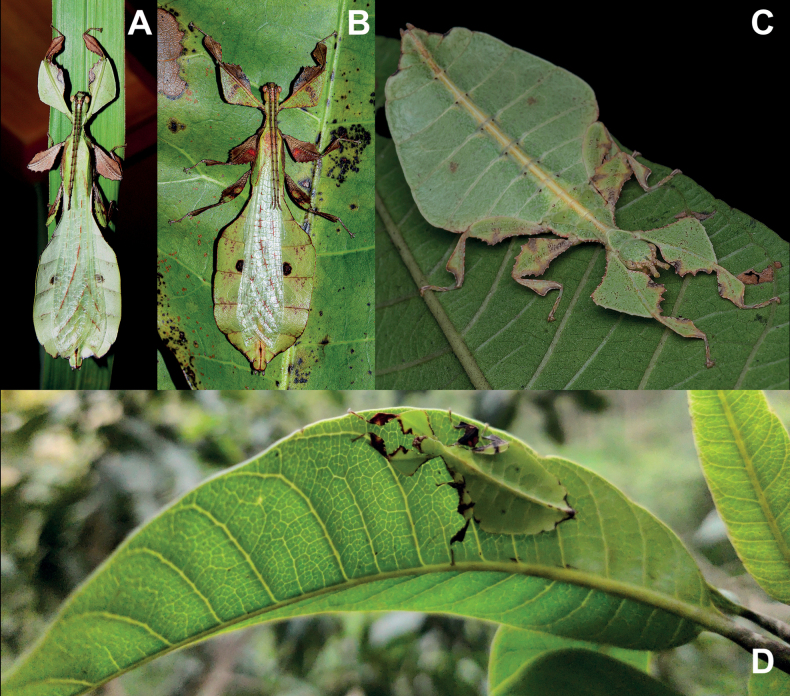
Live *Pulchriphylliumanangu* sp. nov. **A** adult male, dorsal habitus observed October 2006 in B.R. Hills, Karnataka, India by iNaturalist user @prashanthns (Prashanth N S) (https://www.inaturalist.org/observations/31929431) **B** adult male, dorsal habitus observed November 2019 in Pakkalakunja, Karnataka, India by iNaturalist user @sanath_ramesh_manimoole (Sanath R M) (https://www.inaturalist.org/observations/103401996) **C** male nymph, dorsal habitus observed February 2020 in Shimoga, Karnataka, India by iNaturalist user @girishgowda (Girish Gowda) (https://www.inaturalist.org/observations/104655374) **D** nymph, dorsal habitus, hiding under a leaf in the daylight observed August 2020 near Agali, India by iNaturalist user @manavsajan (https://www.inaturalist.org/observations/56588953). Uploaded to iNaturalist and used under license CC BY-NC 4.0.

Figs 8E , 31 , 32 , 33

##### Material examined.

***Holotype*** ♂: “WS 415; India: Karnataka, Agumbe Ghats, Canopy Light Trap, N 13 29.386’ E 75 04.537’ 10-X-2004” (Fig. 31). Deposited in Brigham Young University (BYU), Monte L. Bean Life Science Museum, Provo, Utah, USA. ***Paratypes***: (3♂♂, 3♀♀, 2♀♀ nymphs, 1♂ nymph). See Suppl. material 1 for details about paratype specimens, their collection data, and depositories.

##### Differentiation.

*Pulchriphylliumanangu* sp. nov. are morphologically most similar to *Pulchriphylliumbioculatum* and *Pulchriphylliumagathyrsus*, of which the adult morphology is difficult to differentiate, but the freshly hatched nymphs (Fig. 32B, C) allow reliable differentiation. Presently eggs of *Pulchriphylliumanangu* sp. nov. are not known to us.

**Female***Pulchriphylliumanangu* sp. nov. (Fig. 32A) are difficult to differentiate from *Pulchriphylliumbioculatum* and the only feature which appears to be somewhat reliable is the intensity of the serration on the proximal margin of the profemoral exterior lobe with *Pulchriphylliumanangu* sp. nov. tending to have slightly more prominent serration. For adult females *Pulchriphylliumagathyrsus* we have yet to identify a reliable morphological feature for differentiation.

**Male***Pulchriphylliumanangu* sp. nov. has proven impossible to differentiate morphologically from *Pulchriphylliumagathyrsus* due to notable coloration and abdominal shape variation and therefore no consistent differences have been identified (Fig. 33A, B). *Pulchriphylliumanangu* sp. nov. males can be differentiated from *Pulchriphylliumbioculatum* by subtle differences in the profemoral exterior lobe shape (slightly more obtuse and thinner in *Pulchriphylliumanangu* sp. nov.) and the abdominal shape in *Pulchriphylliumbioculatum* tends to be slightly more ovoid vs *Pulchriphylliumanangu* sp. nov. which have abdominal segments V and VI typically with more parallel margins (Fig. 33A, B).

**Freshly hatched nymphs** of *Pulchriphylliumanangu* sp. nov. are very similar to *Pulchriphylliumbioculatum* and only subtle differences have been identified in their coloration. Typically, the overall coloration of *Pulchriphylliumbioculatum* tends to be a bit darker (Fig. 8D), with the profemoral lobes the most reliable difference between the species as *Pulchriphylliumanangu* sp. nov. (Fig. 8E) tend to have lighter colored profemoral lobes than *Pulchriphylliumbioculatum* (Fig. 8D). *Pulchriphylliumanangu* sp. nov. can easily be differentiated from its sister species *Pulchriphylliumagathyrsus* by the coloration of the femoral and tibial lobes vs the head and thorax coloration. In *Pulchriphylliumagathyrsus* the femoral and tibial lobes contrast significantly with the body coloration as the lobes are dark brown/black while the head and thorax are bright red (Fig. 8F) vs *Pulchriphylliumanangu* sp. nov. which has lobes on the legs with a similar color to the head and thorax (Fig. 8E).

##### Description.

**Female. *Coloration*.** Coloration description is based upon numerous images of live individuals from iNaturalist (Fig. 32A). To date we have only seen records of females where their base coloration was pale green with some areas highlighted in yellow/tan (such as the antennae and venation of the tegmina). In a few individuals we have seen some areas marked with spotting of tan/brown coloration on the abdomen, tegmina, and femoral lobes, but these areas tend to be rather minimal. We have yet to observe extreme color variations in this species.

***Morphology*. *Head*.** Head capsule is slightly longer than wide, with a vertex that is marked throughout by moderately formed granulation (that is typically a paler green than the head capsule base color) and a posteromedial tubercle which is notably larger than the other granulation (and is often brown in color). Frontal convexity finely pointed, with several short setae on the apex. Compound eyes slightly protruding from the head capsule, not overly large, taking up ca ¼ of the head capsule lateral margins. ***Ocelli absent*.** Antennal fields slightly wider than the width of the first antennomere. ***Antennae*.** Antennae consist of nine segments (including scapus and pedicellus), with the terminal segment approximately the same length as the preceding 2⅓ segments’ lengths combined. Antennomeres I–VIII are marked with slight granulation and sparse short setae. ***Thorax*.** Pronotum with slightly convex anterior margin and gently converging lateral margins, which converge to the posterior margin that is ⅔ the width of the anterior margin. The pronotum surface is slightly lumpy and marked with a distinct sagittal furrow and slight central perpendicular furrow. The pronotum has distinct, smooth anterior and lateral rims. Prosternum, mesosternum, and metanotum are covered sparsely with nodes that are irregularly spaced throughout the surface, but all relatively small. Prescutum approximately as long as wide, with lateral rims marked by 10–12 small tubercles relatively evenly sized and spaced, giving the lateral rims a rough appearance. The prescutum anterior rim is distinct but not strongly protruding and the rim surface is relatively smooth. Prescutum surface slightly raised along the sagittal plane and the surface is only slightly marked with granulation. The mesopleura start to diverge slightly posterior to the prescutum anterior rim and diverge uniformly with straight margins. The mesopleuron margin is marked throughout with five or six larger tubercles and six or seven smaller tubercles interspersed throughout. Mesopleuron surface slightly granular. ***Wings*.** Tegmina long, typically reaching significantly onto abdominal segments VII or VIII. Tegmen venation; the subcosta (Sc) is the first vein in the forewing and runs parallel with the margin for the anterior ½, and then bends slightly and runs towards the margin where it terminates ca ¼ of the way through the tegmen length. The radius (R) spans the central portion of the forewing with two subparallel branched veins; the first radius (R1) branches ca ¼ of the way through the tegmen length and terminates slightly proximal to the midline, and the radial sector (Rs) branches ca ⅓ of the way through the tegmen length and terminates ca ⅗ of the way through the length. The media (M) is bifurcate with media anterior (MA) splitting near the middle of the tegmen length and the media posterior (MP) splits ca ⅗ of the way through the length. The media anterior and media posterior terminate near to the posterior ¼ of the tegmen after arcing gently and running parallel with each other. The cubitus (Cu) is bifurcate, branching near the posterior ¼ of the wing into the cubitus anterior (CuA) and cubitus posterior (CuP) which both terminate near the tegmen apex. The first anal vein (1A) is simple and fuses with the cubitus ca ¼ of the way through the tegmen length. Ala rudimentary, no more than a nub. ***Abdomen*.** Abdominal segments II through the anterior ⅔ of segment IV diverge strongly, the remainder of IV diverges only slightly to segment V which is the widest segment, followed by segments VI and VII which converge slightly with straight margins to VIII through X which converge more significantly to the broadly rounded apex. ***Genitalia*.** Subgenital plate starts on the anterior margin of tergum VIII, is moderately broad, and extends to the anterior margin of tergum X, ending in a blunt apex. Gonapophyses VIII are moderate in length, extending halfway onto tergum X, and are moderately broad; gonapophyses IX are smaller and hidden under gonapophyses VIII. The cerci are flat, not strongly cupped, with a granular surface and numerous small setae. ***Legs*.** Profemoral exterior lobe broad, and somewhat recurved with a slightly acute angle and a greatest width ca 5 ½ to 6× the width of the profemoral shaft. Profemoral exterior lobe proximal margin is marked with four or five distinct serrate teeth, and the distal margin is smooth, lacking serration. Profemoral interior lobe is only situated on the distal ⅔ of the profemora, ca 3× as wide as the greatest width of the profemoral shaft and is roundly arcing. The proximal margin of the profemoral interior lobe is relatively smooth and the distal margin is marked with three or four dulled, weakly formed teeth with looping gaps between them. The mesofemoral exterior lobe is roundly triangular and arcs from end to end. The mesofemoral exterior lobe proximal margin is straight and smooth, while the distal margin is slightly rounded and marked with four or five serrate teeth. The mesofemoral interior lobe is slightly wider than the mesofemoral shaft, and the exterior lobe is ca 1½× wider than the mesofemoral shaft. The mesofemoral interior lobe arcs unevenly end to end with seven or eight dulled teeth throughout the length, and the distal end slightly wider than the proximal end. The metafemoral exterior lobe is thin and smooth, hugging the metafemoral shaft and lacks dentition. The metafemoral exterior lobe is thin on the proximal ⅓ and then a gently arcing lobe on the distal ⅔ approximately as wide as the metafemoral shaft width. The metafemoral exterior lobe has moderate serration throughout with those on the distal ½ more prominent. Protibial exterior lobe unevenly arcing with a smooth margin from end to end with a steady increase in width from the proximal to the distal ends. Protibial interior lobe also spans the entire length in a smooth, rounded triangle, with the widest point on the distal ⅓. Mesotibiae and metatibiae lacking interior lobes, with exterior lobes having a similar rounded triangular shape weighted towards the distal end and ca 2× as wide as the shafts on which they are found.

***Measurements of paratype females* [mm].** Length of body (including cerci and head, excluding antennae) 76.6–78.7, length/width of head 8.9–9.4/6.9–7.1, antennae 4.0–4.1, pronotum 5.2–5.5, mesonotum 4.5–4.7, length of tegmina 44.7–47.6, greatest width of abdomen 34.9–35.4, profemora 19.5–19.9, mesofemora 14.0–14.4, metafemora 15.8–16.1, protibiae 9.8–10.0, mesotibiae 9.0–9.4, metatibiae 11.9–12.2.

**Male. *Coloration*.** Coloration description based upon numerous images of live individuals recorded on iNaturalist (Fig. 33A, B). Base coloration variable, ranging from yellow to pale green. Throughout the body are variable patches of tan/brown, primarily found on the abdomen, venation of the wings, and lobes of the legs. Typically, the protibiae and the profemoral interior lobe are brown as well as the antennae. Abdominal segment V has a set of large, dark eye spots.

***Morphology*. *Head*.** Head capsule approximately as long as wide, with a vertex that is weakly granular; posteromedial tubercle small but distinctly raised from the capsule surface (Fig. 31D). Frontal convexity finely pointed, with two or three short setae near the apex. Compound eyes notably bulbous occupying ca ⅖ of the head capsule lateral margins. Three ocelli are well-developed and located between and slightly posterior to the compound eyes. ***Antennae*.** Antennae (including the scapus and pedicellus) consist of 22 or 23 segments. The scapus and pedicellus are bare, all other segments are covered in dense, thin setae that are as long as or longer than the antennal segment is wide. The terminal three segments have shorter and denser setae than the other segments. Each antennomere on the distal end projects ventrally slightly, so the overall antenna has a serrate appearance. ***Thorax*.** Pronotum with anterior margin gently concave and lateral margins that are nearly straight and converge to a straight posterior margin that is approximately ½ the width of the anterior rim (Fig. 31C). Anterior and lateral margins of the pronotum with distinct rims and the posterior margin lacks a distinct rim (Fig. 31C). Face of the pronotum is marked by a slightly wrinkled surface, a distinct sagittal furrow, and a small perpendicular furrow near the center. The prosternum, mesosternum, and metasternum surfaces are nearly smooth, with slightly wrinkled surfaces and sparse granulation. The prescutum is slightly longer than wide, with lateral margins converging to the posterior which is slightly narrower than the anterior rim width (Fig. 31C). The lateral rims of the prescutum are marked with nodes throughout, giving the margins a rough textured appearance. The surface of the prescutum is somewhat raised along the sagittal plane and this crest has a weakly granular texture while the remainder of the surface is relatively smooth. The prescutum anterior rim is distinct, but not prominent, with a surface that is relatively smooth. The mesopleura are narrow on the anterior then diverge more prominently from the anterior to the posterior. Lateral margins of the mesopleurae with granulation throughout, giving the margin a rough textured appearance. Mesopleuron surface relatively smooth. ***Wings*.** Tegmina short, extending ca ½ onto abdominal segment II. Tegmen wing venation: the subcosta (Sc) is the first vein and runs relatively straight for ca ½ of the tegmen length before terminating on the wing margin. The radius (R) spans the entire length of the tegmen, running as the radial sector (Rs) straight through the center to the apex after the first radius (R1) branches near the center of the tegmen and runs to the margin on the posterior ⅓ where it terminates. The media (M) spans the entire length of the tegmen, running parallel with the radius and radial sector and terminates at the tegmen apex as the media anterior (MA) after the branching of a weakly formed media posterior (MP) near the middle of the tegmen and runs at an angle towards the apex. The cubitus (Cu) runs through the tegmen surface angled away from the media (M) for ca ⅓ of the length to the tegmen margin and then runs along the margin where it fades before meeting the apex. The first anal (1A) vein runs subparallel to the cubitus until they meet ca ¼ of the way through the tegmen length. The alae are well-developed in an oval fan configuration, reaching onto abdominal segment VIII or IX. Ala wing venation: the costa (C) is present along the entire foremargin giving stability to the wing. The subcosta (Sc) is fused with the radius for the anterior ⅗ of the ala length, then splits and runs parallel with the wing margin until it fades before reaching the apex. The radius (R) branches ca ⅔ of the way through the ala length into the first radius (R1) and radial sector (Rs) which run gradually diverging through the first ½ of their lengths, then run parallel until fusing with the cubitus at different locations on the posterior ⅕ of the wing. The media (M) branches early, near the anterior ⅛ into the media anterior (MA) and the media posterior (MP) which run diverging for ⅓ of their lengths, then parallel for ⅓ of their lengths, and converging for the final ⅓. The media anterior fuses with the cubitus (Cu) near the posterior ¼ of the ala length. The media posterior fades before fully fusing with the cubitus. The cubitus runs unbranched and terminates at the wing apex after the media anterior, first radius, and radial sector fuse with it at different locations. Of the anterior anal veins, the first anterior anal (1AA) fuses with the cubitus slightly distal to the point where the media branches into the media anterior and media posterior and then the first anterior anal branches from the cubitus ¾ of the way through the wing length where it uniformly diverges from the cubitus until it terminates at the wing margin. The anterior anal veins two–seven (2AA–7AA) have a common origin and run unbranched in a folding fan pattern of relatively uniform spacing to the wing margin. The posterior anal veins (1PA–6PA) share a common origin separate from the anterior anal veins and run unbranched to the wing margin with slightly thinner spacing than the anterior anal veins. ***Abdomen*.** The general abdominal shape is ovular. Abdominal segments II through the anterior ⅔ of IV are gradually diverging, the posterior ⅓ of segment IV diverges less prominently, V through VII are subparallel or slightly converging, VIII through X converge rapidly to the apex. ***Genitalia*.** Poculum broad and rounded, ending in a straight margined apex that passes beyond the anterior margin of segment X (Fig. 31E). The cerci are slightly cupped, covered in a partially granulose surface with sparse short setae throughout. The vomer is broad and slightly longer than wide, with relatively straight sides evenly converging to the apex, which is armed with a single thick upwards turning hook. ***Legs*.** Profemoral exterior lobe broad and slightly obtuse angled, ca 1½× widener than the interior lobe. Profemoral exterior lobe proximal margin slightly granular, and distal margin with small serration throughout the length (Fig. 31C). Profemoral interior lobe begins approximately ⅓ of the way through the profemoral length, and arcs gently with a maximum width of ca 2½× the profemoral shaft width. Profemoral interior lobe is marked by three to four teeth on the distal ½ of the lobe, the proximal most is small, followed by two or three broadly triangular teeth. Mesofemoral exterior lobe is roundly triangular, relatively evenly weighted from end to end, and broad, with the proximal margin straight and smooth, and the distal margin slightly arcing and marked by four or five prominent serrate teeth. The greatest width of the mesofemoral exterior lobe is ca 2× the width of the mesofemoral shaft. The mesofemoral interior lobe is slightly thinner than the exterior lobe and is unevenly weighted from end to end with the widest portion near the distal ⅓. The proximal ⅓ of the mesofemoral interior lobe is narrow, followed by a rounded arc on the distal ⅔. There is unevenly spaced and variable sized serration throughout the full margin of the mesofemoral interior lobe (typically six or seven serrate teeth). Metafemoral exterior lobe arcs thinly along the metafemoral shaft, with a maximum width slightly thinner than the metafemoral shaft width. Metafemoral exterior lobe lacks dentition throughout most of its length. Metafemoral interior lobe is narrow and straight for the proximal ½ and then on the distal ½ there is a slightly expanding lobe that is slightly wider than the shaft’s width. There is serration of variable size and spacing throughout the length (eight or nine teeth). Protibial exterior lobe is a thin scalene triangle weighted towards the distal end which is 1–1½× as wide as the protibial shaft width. The protibial interior lobe is also a scalene triangle but it is slightly wider than the exterior lobe, ca 2½× as wide as the protibial shaft width, and the widest portion is weighted near the distal ⅖. Mesotibial exterior lobe is roughly a thin, smooth triangle which is weighted towards the distal ⅓ and has a greatest width ca 1½× the width of the mesotibial shaft. Metatibial exterior lobe roughly a thin smooth triangle which is weighted towards the distal ⅓ and has a greatest width ca 3× the width of the metatibial shaft. Mesotibiae and metatibiae lack interior lobes.

***Measurements of holotype male* [mm].** Length of body (including cerci and head, excluding antennae) 63.1, length/width of head 3.7/3.9, antennae (missing from holotype specimen), pronotum 3.1, mesonotum 2.7, length of tegmina 13.2, length of alae 49.1, greatest width of abdomen 22.5, profemora 14.2, mesofemora 11.6, metafemora 9.2, protibiae 6.9, mesotibiae 6.8, metatibiae 9.1.

##### Newly hatched nymphs.

(Fig. 32B, C). The general color throughout the body ranges from orange to bright vermillion with muddled lighter and darker areas. The basitarsi are white/yellow, with the remaining tarsal segments similar to the body base color. All tibiae have well-developed exterior lobes of similar shapes, all a rounded arc that is widest approximately halfway through the tibial length. The protibial interior lobe is prominent, a similar size to the exterior lobe, but not as strongly angled. The protibial lobes have widths that are ca 2× as width of the protibia shaft width. Profemoral interior lobe occupies the distal ⅔ and is slightly < 2× the profemoral shaft width. The profemoral interior lobe has two weakly formed teeth and is slightly angled. Profemoral exterior lobe has a clearly defined obtuse angle with a distal margin that is marked by weak dentition. The profemoral exterior lobe is ca 3× the width of the profemoral shaft. All femoral lobes have mild serration (with the exterior lobes marked by slightly more prominent serration). The meso- and metafemoral interior lobes span end to end, but are more heavily weighted towards the distal end, with the proximal end thin. The meso- and metafemoral exterior lobes have a similar shape to their interior lobe counterparts but are less angled than the interior lobe. The meso- and metafemoral interior and exterior lobes have two or three small teeth on the distal end, with the teeth of the mesofemoral interior lobe less prominent than the mesofemoral exterior lobe, and the metafemoral interior lobe more prominent than the metafemoral exterior lobe. The head and thorax base color are variable, ranging from vermillion to light orange, often muddled in color. The thorax is of a similar color pattern to the head, but with margins marked in a darker color, ranging from reddish to brown. The abdomen base color is of a similar muddled coloration to the head/thorax, but typically with darker markings. Segments II through VIII are marked near the middle with an undulating darker pattern, with the inner area a lighter, uniform color, and the remainder of the segment of a darker muddled coloration. Segments IX and X are mostly muddled brown without a distinct pattern. The abdomen is notably wide, with a maximum width only slightly less than the abdomen length. The widest point of the abdomen is abdominal segment V.

##### Etymology.

Proper noun, named after Anangu, one of the Yakshi, or tree nymphs of Tamil mythology from southern India (Pai-Dhungat 2020). This name was chosen to reference the range of this species in southern India and because leaf insects are mysterious, seldom seem creatures, likening them to spirits of the trees. Additionally, the Yakshi are often associated with mango trees (a favorite host plant of leaf insects).

##### Distribution.

At present known from multiple states in southwestern India (Fig. 15). States that this species have been recorded from are Goa, Karnataka, Kerala, and Tamil Nadu. See Suppl. material 1 for more details.

##### Remarks.

Despite being an undescribed species, leaf insect observations from India are commonly recorded on the citizen science platform iNaturalist. These submitted observations allowed a significant look into adult male (Fig. 33A, B) and female (Fig. 32A) morphology, as well as the coloration of the freshly hatched nymphs (Fig. 32B, C). Freshly hatched nymphs are quite active after they hatch, brightly colored, and travel from the forest floor up into the canopy, thus allowing them to be readily observed by citizen scientists. Unfortunately, the eggs of this species are presently unknown, as the eggs are lost within leaf litter and never observed/recorded. Hopefully a captive colony in India one day will reveal the unknown egg morphology of this species and allow differentiation from congenerics. Phylogenetic analysis and morphological comparison with congenerics have now allowed this commonly encountered species to be recognized as undescribed and endemic to southwestern India.

Within the southwestern Indian state of Kerala, the Malayali people call this species “Pera Rani” (meaning “Queen of Guava plant” in Malayalam) a fitting common name as phylliids are frequently observed by farmers of guava (pers. comm. Gavas Ragesh, Kerala Agricultural University). Local farmers additionally report this species as being observed on cashew and mango trees, but never in significant enough populations to be considered a pest. Of these three common agricultural plants, only mango is native to Asia (with the other two native to the tropics of the western hemisphere; Morton 1987), but little is presently known about other potential native host plants for leaf insects.

#### ﻿ Pulchriphyllium delislei

Taxon classificationAnimaliaPhasmatodeaPhylliidae

sp. nov.

6A077A16-A393-5BA7-9A12-F5323D0FFA2C

https://zoobank.org/9260AEF5-42FA-48F2-AA6D-EEC9EFD21085

Figs 34 , 35

##### Material examined.

**Figure 34.**
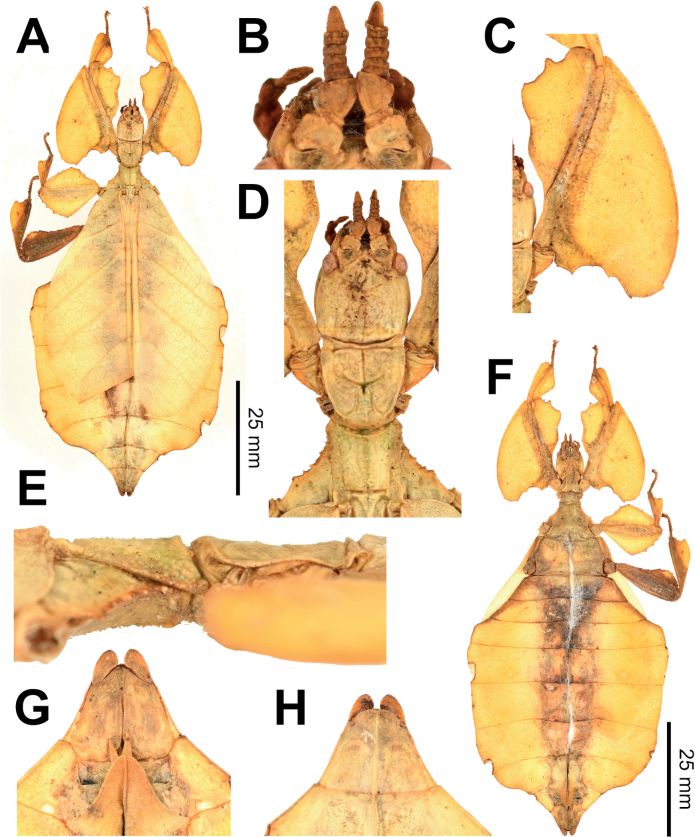
Female holotype *Pulchriphylliumdelislei* sp. nov. (IMQC) **A** dorsal, habitus **B** antennae, dorsal **C** right profemora, dorsal **D** head and thorax, dorsal **E** thorax, lateral, anterior to the right **F** ventral, habitus **G** genitalia, ventral **H** terminalia, dorsal.

***Holotype*** ♀: “Indonesia: South Kalimantan, Mt. Meratus, VIII.2020; DNA sample SLT050” (Fig. 34). Deposited in the Montreal Insectarium, Quebec, Canada (IMQC).

**Figure 35.**
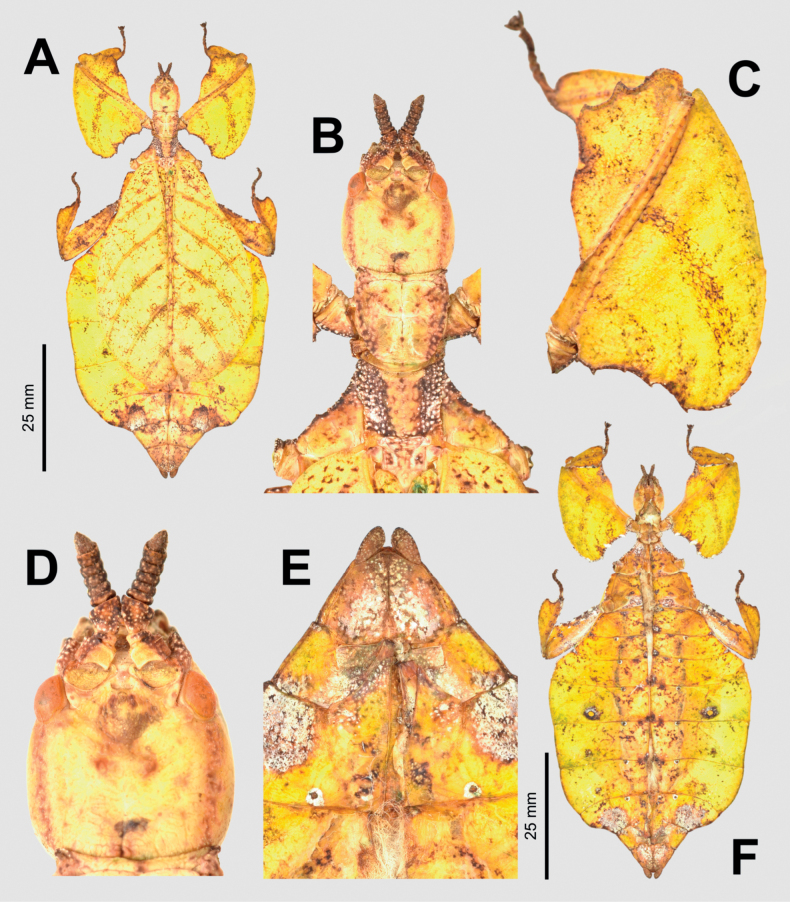
Female paratype *Pulchriphylliumdelislei* sp. nov. (IMQC) **A** dorsal, habitus **B** details of the head through thorax, dorsal **C** details of the front leg, dorsal **D** details of head and antennae, dorsal **E** genitalia, ventral **F** ventral, habitus.

***Paratype*** ♀: “Indonesia, Kalimantan, Mt. Besar VII.2018; DNA sample SLT006” (Fig. 35). Deposited in the Montreal Insectarium, Quebec, Canada (IMQC).

##### Differentiation.

Males, freshly hatched nymphs, and eggs are currently unknown. At present only known from two female specimens, therefore little is known of the intraspecific variation of this species. Additionally, throughout the “bioculatum”-like species ranges other species show a decent amount of variation in their abdominal shapes/femoral lobe spination, so a reliable morphological feature has yet to be identified for differentiation. Also, with two congenerics from Borneo with the “bioculatum”-like morphology only known from males, and this species only known from a female, nothing can be said about morphological differentiation presently for these Bornean species. Only DNA analyses have allowed differentiation at this time (Fig. 2).

##### Description.

**Female. *Coloration*.** The coloration description is based upon the dried type specimens which appears to have been well-preserved with minimal discoloration (Fig. 35). Both the holotype and paratype specimens are yellow form individuals, but as these are the only specimens currently known, we cannot say whether or not this color form is typical, or like many phylliid species, simply one of multiple color forms possible. The base coloration is straw yellow, with muddled areas of orange, tan, and brown, giving the specimen a decaying leaf resemblance. The thorax (Fig. 35B), ventral surfaces of the legs, spiracles on the abdominal sternites, and abdominal segments VIII, IX, and X (Fig. 35E) have white speckling which looks mold-like, further enhancing the decaying leaf appearance. The antennae, margins of the mesonotum, venation of the tegmina, and the terminal abdominal segment are the darkest colored areas, from tan to brown in color. Abdominal segment V is marked with a pair of gray spots (Fig. 35F).

***Morphology*. *Head*.** Head capsule is as long as wide, with a vertex that is marked throughout by weakly formed granulation (Fig. 35D). Frontal convexity blunt and fully covered by granulation and short setae (Fig. 35D). Compound eyes slightly protruding from the head capsule, small, occupying ca 2/7 of the head capsule lateral margins (Fig. 35D). ***Ocelli absent*.** Antennal fields slightly wider than the first antennomere width. ***Antennae*.** Antennae consist of nine segments including scapus and pedicellus. Antennomeres IV through VIII are all similar lengths/widths and the terminal antennomere is approximately as long as the previous two antennomeres. Antennomeres I–VIII are sparsely marked with small transparent setae and granulation. The terminal antennomere is covered in short, dense setae, giving it a fuzzy appearance (Fig. 35D). ***Thorax*.** Pronotum boxy, with a similar width to length. Anterior margin slightly convex, lateral margins subparallel until the posterior ¼ then converge slightly, posterior margin straight and ca ⅘ the width of the anterior margin (Fig. 35B). The pronotum surface is slightly lumpy and wrinkled, with a distinct furrow along the sagittal plane and smaller furrows perpendicular near the center (Fig. 35B). The pronotum has a distinctly formed anterior rim and moderately formed posterior and lateral rims (Fig. 35B). Prosternum surface slightly granular (more prominently on the posterior ⅓). Mesosternum anterior ¼ and posterior margin slightly granular, remainder relatively smooth. Metasternum relatively smooth except for a patch on the posterior which is slightly granular. Prescutum anterior margin slightly wider than the prescutum length, lateral margins heavily marked with granulation (Fig. 35B). Prescutum anterior rim moderately formed but not strongly protruding, central third of the anterior rim moderately marked with granulation, and the lateral third of the anterior rim on each side roughly textured (Fig. 35B). Prescutum surface central ⅓ raised gently along the sagittal plane and slightly granular, lateral ⅓ on each side rough, ornamented prominently by granulation (Fig. 35B). The mesopleura diverge consistently throughout their lengths and are marked with slight granulation throughout and a singular, prominently raised spine near the posterior ⅖ (Fig. 35B). Mesopleuron face relatively smooth except for the anterior margin which has notable granulation (Fig. 35B). ***Wings*.** Tegmina long, reaching ¾ the way onto abdominal segment VII. Tegmen venation; the subcosta (Sc) is the first vein in the tegmen, running parallel with the distal margin for the first ⅔, and then running slightly towards the margin where it terminates. The radius (R) spans approximately the anterior ⅖ of the tegmen with two subparallel veins; the first radius (R1) branches ca 1/7 of the way through the tegmen length and terminates slightly < ⅓ of the way through the tegmen length when it meets the margin; the radial sector (Rs) branches slightly < ⅓ of the way through the tegmen length, arcs gently, and terminates on the tegmen margin slightly distal to the midlength. There is a weak continuation of the radius following the prominent Rs branching which continues as a short and thin R–M crossvein that fades before solidly connecting the two veins. The media (M) is bifurcate with the media anterior (MA) terminating near to the posterior ¼ of the tegmen and the media posterior (MP) terminating near to the posterior 1/9 of the tegmen. The cubitus (Cu) is also bifurcate, branching near the posterior ⅕ of the tegmen into the cubitus anterior (CuA) and the cubitus posterior (CuP) which both terminate at or very near the tegmen apex. The first anal vein (1A) is simple and fuses with the cubitus early on. The radius, media, and cubitus all run side by side/slightly fused in places, no notable spacing between them. Alae rudimentary, only small nubs. ***Abdomen*.** Abdominal segments II through the anterior ½ of IV strongly diverging. Posterior ½ of IV to the anterior ½ of V weakly diverging to the widest point of the abdomen. The remainder of V through VII converge slightly, followed by VIII through X converging more strongly, giving the abdomen an overall spade-shaped appearance. ***Genitalia*.** Subgenital plate starts at the anterior margin of tergum VIII, is moderately broad (occupying the central ½ of the posterior margin of tergum VIII), is short (only extending just shy of reaching the anterior margin of tergum X) and has margins which are slightly convex to a blunt apex (Fig. 35E). Gonapophyses VIII are short, only reaching slightly onto abdominal tergum X; gonapophyses IX are shorter and narrower, hidden below gonapophyses VIII (Fig. 35E). Cerci flat, with a granular surface and few detectable setae (Fig. 35E). ***Legs*.** Profemoral exterior lobe broad (ca 1¾ x the width of the interior lobe), with the proximal margin slightly undulating away from the profemoral shaft at approximately a 90-degree angle, and the distal margin arcs giving the exterior profemoral lobe a broad rounded appearance (Fig. 35C). Profemoral exterior lobe proximal margin with distinct, evenly spaced serration (three or four prominent teeth and one or two minor teeth; Fig. 35C). Profemoral exterior lobe distal margin with slight granulation throughout its length (with the proximal most slightly more prominent). Profemoral interior lobe begins ca ⅓ of the way through the profemoral shaft length, arcs into a rounded lobe that at its widest point is ca 3× as wide as the greatest width of the profemoral shaft, and the distal margin is marker with two or three dulled, serrate teeth and the distal most end has a rounded projection (Fig. 35C). Mesofemoral interior and exterior lobes of similar widths. Both mesofemoral lobes are roundly triangular, with the interior lobe slightly weighted to the proximal ⅖ and the exterior lobe slightly more evenly weighted, but with the distal ½ slightly thicker than the proximal ½. The mesofemoral interior lobe has serration throughout the length (six or seven dulled, serrate teeth). The mesofemoral exterior lobe proximal ½ is straight and smooth, the distal ½ is marked with six serrate teeth. Metafemoral interior lobe narrow for most of the length, only broadening slightly on the distal ⅓. Metafemoral interior lobe with distinct serration throughout the length (five to seven teeth). Metafemoral exterior lobe is thin and smooth, hugging the metafemoral shaft and lacks dentition. Protibiae with interior and exterior lobes fully spanning the length. Protibial interior lobe at its widest ca 2× the width of the protibial shaft. Protibial interior lobe a rounded scalene triangle with the widest point on the distal ⅓. Protibial exterior lobe, gradually increasing in width from the proximal to the distal end, with the greatest width on the distal end, slightly wider than the protibial shaft width. Mesotibiae lacking interior lobes, exterior lobe well-developed into a rounded right triangle weighted to the distal end of the shaft with a maximum width ca 1½× the width of the mesotibial shaft. Metatibiae lacking interior lobes, but there is a well-developed metatibial exterior lobe that is nearly right angled on the distal end with a maximum width of ca 2× the metatibial shaft width.

***Measurements of holotype female* [mm].** Length of body (including cerci and head, excluding antennae) 84.5, length/width of head 7.6/6.0, antennae 3.8, pronotum 5.4, mesonotum 4.7, length of tegmina 50.6, greatest width of abdomen 48.9, profemora 18.5, mesofemora 8.4, metafemora 14.7, protibiae 8.0, mesotibiae 7.5, metatibiae 13.3.

***Measurements of paratype female* [mm].** Length of body (including cerci and head, excluding antennae) 78.9, length/width of head 7.7/6.2, antennae 4.5, pronotum 5.4, mesonotum 4.8, length of tegmina 44.1, greatest width of abdomen 40.0, profemora 15.6, mesofemora (missing from paratype), metafemora 15.2, protibiae 9.5, mesotibiae (missing from paratype), metatibiae 12.6.

##### Etymology.

Patronym, named after Gilles Delisle (Canada). Friend of the authors and enthusiastic entomologist. Gilles Delisle is a birdwing butterfly specialist and research associate of the Montreal Insectarium. His passion for birdwing butterflies allowed him to travel many times throughout Indonesia and Papua New Guinea. He donated his butterfly collection after many years of studies to the Montreal Insectarium, Canada.

##### Distribution.

At present only known from the localities of Mt. Meratus and Mt. Besar, Kalimantan, Indonesia.

##### Remarks.

When the specimen was preliminarily reviewed, three possible identifications were assumed. First, it was considered that this might represent the unknown female *Pulchriphylliumagnesagamaae* or *Pulchriphylliumfredkugani* (both species currently only known from males from northern Borneo). Or secondly, that this specimen might simply be a range expansion for *Pulchriphylliumbioculatum*, a species which has been confirmed via molecular analyses to occur on southwestern Borneo as well as peninsular Malaysia (Fig. 2; sample #16-221). Interestingly, despite being morphologically difficult to differentiate from other “bioculatum”-like species, upon phylogenetic analysis this sample did not cluster with any of the “bioculatum”-like species (Fig. 2). *Pulchriphylliumdelislei* sp. nov. instead represents yet another instance where the “bioculatum”-like morphology has been revealed to be non-monophyletic (Fig. 2).

## ﻿Discussion and conclusions

Due to the limited sampling of leaf insect specimens in collections and the challenges of species delimitation, often poorly known/rarely encountered species are misidentified as members of better-known clades. Additionally, the pronounced sexual dimorphism has sometimes led to males and females having been occasionally mistakenly identified as separate species. Here, we have described seven new phylliid species: *Phylliumiyadaon* sp. nov. from Mindoro Island, Philippines; *Phylliumsamarense* sp. nov. from Samar Island, Philippines; *Phylliumortizi* sp. nov. from Mindanao Island, Philippines; *Pulchriphylliumheracles* sp. nov. from Vietnam; *Pulchriphylliumdelislei* sp. nov. from South Kalimantan, Indonesia; *Pulchriphylliumbhaskarai* sp. nov. from Java, Indonesia; and *Pulchriphylliumanangu* sp. nov. from southwestern India. These species were uncovered within the phylogenetic tree inferred by Bank et al. (2021) and when coupled with additional sampling, the phylogenetic analyses helped to clarify their taxonomic status and to justify their description as new species herein (see tree in Fig. 2). Tremendous progress has been made in the identification of undescribed taxa as a majority of phylliid species now have associated DNA sequences, a necessary resource for future species descriptions, particularly to aid in the discovery of cryptic leaf insect species (Cumming et al. 2020b).

Molecular work has been especially useful in the differentiation within *Pulchriphyllium*. Historically, many species with “*bioculatum*”-like morphology (females that have tapered abdomens and males with rounded/ovoid abdomens) have been called forms or subspecies of *Pulchriphylliumbioculatum*. This previous classification of *Pulchriphylliumbioculatum* had the species occurring within a markedly wide geographic range, from the Seychelles to north India and Bangladesh, through mainland Asia to Thailand, peninsular Malaysia, and south into Sumatra. However, DNA sequencing data has revealed that what has previously been considered “*bioculatum*” does not form a monophyletic group, and instead the true *Pulchriphylliumbioculatum* (from West Malaysia and adjacent areas: Fig. 15A) has been recovered as sister taxon to *Pulchriphylliumabdulfatahi* and *Pulchriphylliumfredkugani* (both from Borneo), while the Indian and Sri Lankan “*bioculatum*”-like species are sister taxa and are not closely related to true *Pulchriphylliumbioculatum* (Fig. 2).

These newly described species not only help to expand our knowledge of phylliid diversity, but also help to highlight recent and yet to be undertaken areas of research. For example, investigation into the morphological and functional diversity of phylliid eggs is revealing interesting stories of abiotic interactions and biotic mimicry (O’Hanlon et al. 2020; Büscher et al. 2023). Several of the herein described species have egg morphologies which fit well within our growing body of knowledge (such as *Phylliumsamarense* eggs which morphologically are rather similar to congeners) while others present a layer of obscure novelty in their form (such as *Pulchriphylliumbhaskarai* with their rigid lateral tubes of unknown function). This is particularly interesting as many phylliid species uniformly mimic simple angiosperm leaves, resulting in adults being difficult to distinguish, with some species only easily differentiable as eggs or freshly hatched nymphs. While our understanding of phylliid egg diversity/functionality is beginning to take form, our understanding of the purpose of the diversity in freshly hatched nymph camouflage is still limited (Fig. 8). While several species freshly hatched nymphs bear a striking resemblance to some Mantodea or Hemiptera nymphs, others (such as some *Pulchriphyllium* spp.) appear to mimic small, dried leaves, as one might expect on the forest floor (Fig. 8; Cumming et al. 2021b). The reasons behind many freshly hatched nymph morphologies are still enigmatic and none have been tested for veracity, yet their diversity in shape and coloration suggests some evolutionary force must be influencing their often, species-specific forms. Hopefully as more phylliid species life histories are elucidated (such as through captive breeding) our understanding of freshly hatched nymph morphology will take form and thorough investigations can be conducted.

In addition to describing new species based upon freshly collected material, the extensive review of type specimens from historic, little-known taxa has allowed the designation of lectotype specimens and clarified the status of *Pulchriphylliumscythe* and *Pulchriphylliumcrurifolium* as distinct species to reflect their unique morphology and geographic isolation. Continued sampling and molecular sequencing will be crucial in resolving remaining uncertainties and identifying new species in the future.

## Supplementary Material

XML Treatment for Phyllium

XML Treatment for Phyllium samarense

XML Treatment for Phyllium iyadaon

XML Treatment for Phyllium ortizi

XML Treatment for Pulchriphyllium

XML Treatment for Pulchriphyllium scythe

XML Treatment for Pulchriphyllium crurifolium

XML Treatment for Phyllium dardanus

XML Treatment for Pulchriphyllium heracles

XML Treatment for Pulchriphyllium bhaskarai

XML Treatment for Pulchriphyllium anangu

XML Treatment for Pulchriphyllium delislei

## ﻿Additional information

### Author ORCIDs

Royce T. Cumming https://orcid.org/0000-0001-7930-1292

Stéphane Le Tirant https://orcid.org/0000-0001-7463-5621

Jackson B. Linde https://orcid.org/0000-0003-1480-0212

Megan E. Solan https://orcid.org/0000-0002-2298-5336

Evelyn Marie Foley https://orcid.org/0009-0007-9754-212X

Ramon Lavado https://orcid.org/0000-0002-2774-5104

Michael F. Whiting https://orcid.org/0000-0003-1157-888X

Sven Bradler https://orcid.org/0000-0001-9307-1032

Sarah Bank https://orcid.org/0000-0001-6952-1590

### Data availability

All of the data that support the findings of this study are available in the main text or Supplementary Information.

## Supplementary materials

Supplementary material 1 Examined specimens and paratype data with depositories Data type Specimen/observation data Explanation note Taxon information for each specimens examined including sex, type designation, sample ID, collection data, and links to observational data available online. https://binary.pensoft.net/file/887738This dataset is made available under the Open Database License (http://opendatacommons.org/licenses/odbl/1.0/). The Open Database License (ODbL) is a license agreement intended to allow users to freely share, modify, and use this Dataset while maintaining this same freedom for others, provided that the original source and author(s) are credited.Author: Royce T. Cumming

Supplementary material 2 All sampled specimens are listed with their collection data, deposition location, and GenBank accession numbers for the generated sequences. Data type Specimen data Explanation note Taxon information for each specimen used in the phylogenetic analysis including sex, type designation, sample ID, sampling site, and GenBank accession numbers. Newly generated molecular data in bold. https://binary.pensoft.net/file/887739This dataset is made available under the Open Database License (http://opendatacommons.org/licenses/odbl/1.0/). The Open Database License (ODbL) is a license agreement intended to allow users to freely share, modify, and use this Dataset while maintaining this same freedom for others, provided that the original source and author(s) are credited.Sarah Bank, Royce T. Cumming

Supplementary material 3 Multiple sequence alignment of concatenated dataset Data type phylogenetic data https://binary.pensoft.net/file/887740This dataset is made available under the Open Database License (http://opendatacommons.org/licenses/odbl/1.0/). The Open Database License (ODbL) is a license agreement intended to allow users to freely share, modify, and use this Dataset while maintaining this same freedom for others, provided that the original source and author(s) are credited.Author: Sarah Bank

Supplementary material 4 Full-tree figure with outgroups Data type Resulting tree based on re-analysis of the phylogenetic relationships of the 169 taxa from Bank et al. (2021) with the 15 newly added specimens included herein. Explanation note Fully recovered tree with outgroups. https://binary.pensoft.net/file/887741This dataset is made available under the Open Database License (http://opendatacommons.org/licenses/odbl/1.0/). The Open Database License (ODbL) is a license agreement intended to allow users to freely share, modify, and use this Dataset while maintaining this same freedom for others, provided that the original source and author(s) are credited.Author: Sarah Bank
