# Supplementary figures and images for: ﻿On seven undescribed leaf insect species revealed within the recent “Tree of Leaves” (Phasmatodea, Phylliidae)
Source: Zookeys. 2023 Aug 3;1173:145–229. doi: 10.3897/zookeys.1173.104413 (PMC10416092; doi:10.3897/zookeys.1173.104413)

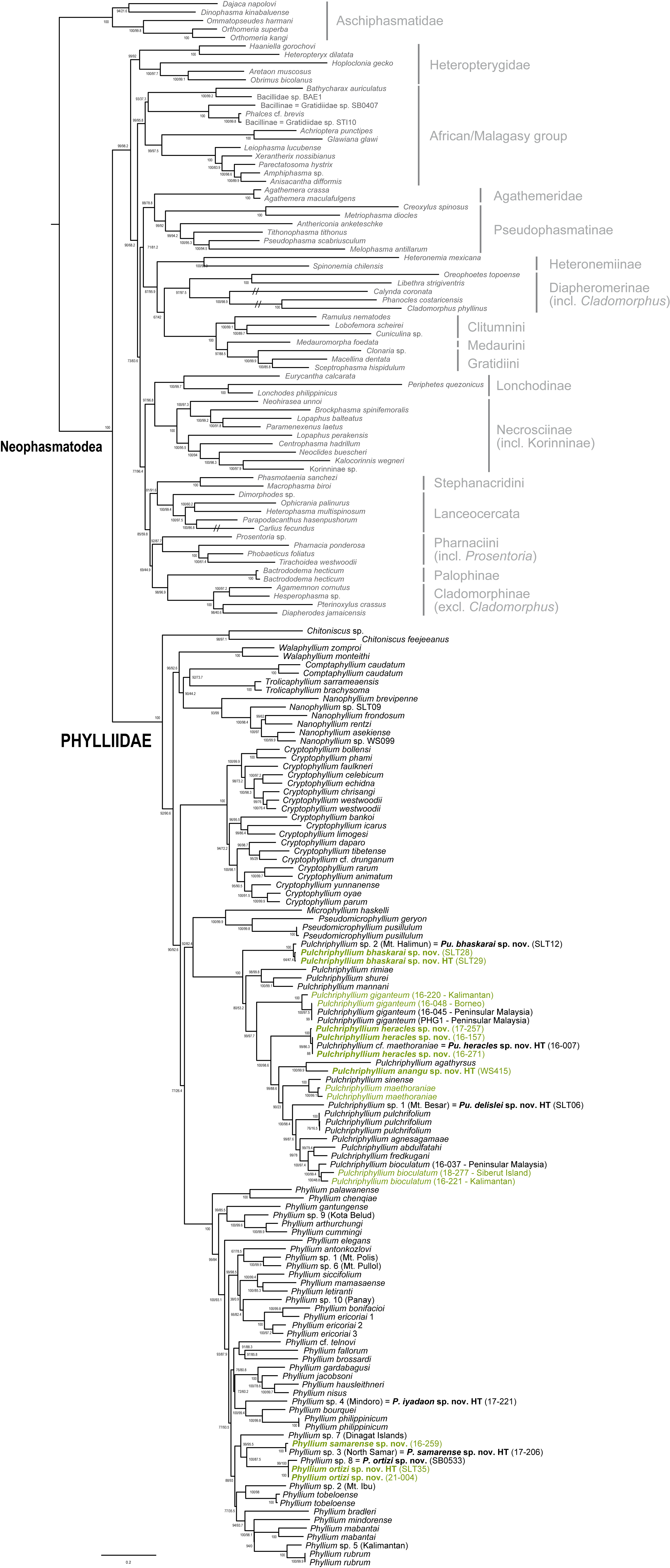

Supplement: Supplementary material 1 — Examined specimens and paratype data with depositories [file zookeys-1173-145_article-104413__-s001.png]
